# Faecal microbiota transplant to ERadicate gastrointestinal carriage of Antibiotic Resistant Organisms (FERARO): a prospective, randomised placebo-controlled feasibility trial

**DOI:** 10.1136/bmjopen-2020-038847

**Published:** 2020-05-25

**Authors:** Blair Merrick, Emily Robinson, Catey Bunce, Liz Allen, Karen Bisnauthsing, Chi Chi Izundu, Jordana Bell, Gregory Amos, Manu Shankar-Hari, Anna Goodman, Debbie L Shawcross, Simon D Goldenberg

**Affiliations:** 1 Centre for Clinical Infection and Diagnostics Research, Guy’s and Saint Thomas’ Hospitals NHS Trust, London, UK; 2 Department of Infectious Diseases, School of Immunology and Microbial Sciences, Faculty of Life Sciences and Medicine, King’s College London, London, UK; 3 School of Population Health and Environmental Sciences, King’s College London, London, UK; 4 Primary Care and Public Health Sciences, King’s College London, London, UK; 5 Pharmacy Department, Guy’s and Saint Thomas’ Hospitals NHS Trust, London, UK; 6 Early Clinical Development Centre of Excellence, IQVIA, Reading, UK; 7 Care of Guy’s and Saint Thomas’ NHS Trust, London, UK; 8 Department of Twin Research and Genetic Epidemiology, School of Life Course Sciences, Faculty of Life Sciences and Medicine, King’s College London, London, UK; 9 National Institute for Biological Standards and Control, Potters Bar, UK; 10 Intensive Care Unit, Guy’s and Saint Thomas’ Hospitals NHS Trust, London, UK; 11 Institute of Liver Studies, Inflammation Biology, School of Immunology and Microbial Sciences, Faculty of Life Sciences and Medicine, King’s College London, London, UK

**Keywords:** bacteriology, microbiology, infection control

## Abstract

**Introduction:**

Antimicrobial resistance is rising, largely due to the indiscriminate use of antimicrobials. The human gut is the largest reservoir of antibiotic resistant bacteria (ARB). Individuals colonised with ARB have the potential to spread these organisms both in the community and hospital settings. Infections with ARB such as extended spectrum beta-lactamase producing enterobacteriales (ESBL-E) and carbapenemase producing enterobacteriales (CPE) are more difficult to treat and are associated with an increased morbidity and mortality. Presently, there is no effective decolonisation strategy for these ARB. Faecal microbiota transplant (FMT) has emerged as a potential strategy for decolonisation of ARB from the human gut, however there is significant uncertainty about the feasibility, effectiveness and safety of using this approach.

**Methods and analysis:**

Prospective, randomised, patient-blinded, placebo-controlled feasibility trial of FMT to eradicate gastrointestinal carriage of ARB. Eighty patients with a recent history of invasive infection secondary to ESBL-E or CPE and persistent gastrointestinal carriage will be randomised 1:1 to receive encapsulated FMT or placebo. The primary outcome measure is consent rate (as a proportion of patients who fulfil inclusion/exclusion criteria); this will be used to determine if a substantive trial is feasible. Participants will be followed up at 1 week, 1 month, 3 months and 6 months and monitored for adverse events as well as gastrointestinal carriage rates of ARB after intervention.

**Ethics and dissemination:**

Research ethics approval was obtained by London—City and East Research Ethics Committee (ref 20/LO/0117). Trial results will be published in a peer-reviewed journal and presented at international conferences.

**Trial registration number:**

ISRCTN registration number 34 467 677 and EudraCT number 2019-001618-41.

**Strengths and limitations of this study**

- The randomised, placebo-controlled design will control for spontaneous loss of carriage of resistant organisms.
- Qualitative data from participant focus groups will inform and influence a potential future trial.
- This study will assess feasibility; however, it is not statistically powered to assess clinically efficacy, which will need to be evaluated in a substantive trial.
- Mechanistic outcomes using metagenomic, metabolomic and host immune analyses could provide insight into the mechanism of action of Faecal microbiota transplant in treatment responders.
- The lack of investigator blinding, and the single-centre design is a limitation.

## Introduction

Antimicrobial resistance (AMR) in enterobacteriales is increasing, fuelled by the indiscriminate use of antimicrobials and inadequate infection control practices. Of greatest concern are extended spectrum beta-lactamase producing (ESBL-E) and carbapenemase producing enterobacteriales (CPE). Rates of ESBL producing bacteria carriage in our local population are 9%, with the majority being CTX-M type.^1^ Rates of detection and infections caused by ESBL-E/CRE are increasing nationally and globally,^2 3^ resulting in a significant burden of attributable death and disability adjusted life years.^4^ Antimicrobial resistant bacteria (ARB) such as ESBL-E/CPE have the capacity to spread between individuals and between organisms through horizontal gene transfer. These organisms have been responsible for several large and prolonged outbreaks worldwide.^5–7^ As well as increased morbidity and mortality, infection with resistant organisms is associated with prolonged hospital stay and increased healthcare costs.^4 8 9^ Hospitalised patients are particularly at risk of acquiring these organisms due to the treatments and procedures they receive, their comorbidities and their high exposure to antimicrobials.^5^

The microbiota of the human gut is a complex ecosystem and the largest reservoir of ARB.^10 11^ A better understanding of the human microbiome has led to a new appreciation for the role indigenous microbes play in protecting us from invading exogenous pathogens. The role of the gut microbiota in defending the host against gastrointestinal pathogens was first described in a mouse model in which streptomycin administered orally to disrupt the gut microbiota resulted in increased rate of Salmonella enterica-related infections.^12^ Antimicrobials disrupt the balance of the delicate gut ecosystem, enabling colonisation by ESBL-E/CPE and other potential pathogens. This is most strikingly evident in patients suffering from Clostridioides difficile infection (CDI), and the remarkable success of modulating this with faecal microbiota transplantation (FMT).^13 14^

Attempts to control carriage of ARB in the gut using selective digestive decolonisation are controversial, have not been widely adopted and are not recommended by expert groups.^15^ Loss of ARB colonisation has been observed in a number of patients when using FMT to treat recurrent CDI.^16^ However, these reports are nearly all case series which are uncontrolled and do not account for spontaneous loss of carriage, which can occur in up to 50% of patients following hospital discharge.^17^

The only published randomised trial of FMT to eradicate gastrointestinal carriage of ESBL-E and CPE was conducted in four academic centres in Geneva, Paris, Utrecht and Tel Aviv.^18^ Patients were randomised in a 1:1 ratio to a 5-day course of colistin and neomycin followed by FMT or no intervention. The primary outcome measure was culture of ESBL-E/CPE from stool 35–48 days following randomisation, which was achieved for 41% (9/22) of patients in the intervention arm versus 29% (5/17) in the control arm. Although the OR for decolonisation success for FMT was 1.7 (95% CI: 0.4 to 6.4), this was not statistically significant, leaving the authors to conclude that the results do not support the routine use of FMT for decolonisation. Although the study was multicentre and included a control group to account for spontaneous loss of carriage, there are several limitations with the design and conduct, making it difficult to draw firm conclusions. First, although designed as a superiority trial with a sample size calculation of 32 in each group, only 39 (61%) patients in total were randomised (due to recruitment problems). Second, patients in the intervention arm received 5 days of colistin and neomycin in addition to FMT, whereas the controls received no intervention. Thus, it is impossible to determine whether the results were due to the antibiotics (likely to have a profound effect on the gut flora) versus FMT. Third, the methods of administration of FMT varied according to recruiting site; capsules were administered in two centres (16 patients), while two used nasogastric administration (six patients). The capsules (15 administered each day over 2 days) were produced from one donation derived from 15–30 g faeces. The nasogastric preparation was derived from 40 g. There is evidence in the context of recurrent CDI that FMT preparations made with less than 50 g faeces result in poorer outcomes than those made with more than this amount.^19^ Thus, a question exists over whether the patients were under dosed, and if repeated administrations (perhaps using different donors) might be more effective. Finally, the study was not placebo controlled or blinded, although the primary outcome of stool culture at 1 month is fairly objective, there is the possibility of introducing bias in an investigator who is aware of the allocation.

Due to the limitations of the above study, the lack of other rigorously conducted, well-controlled studies and the considerable doubt that sufficient patients would be willing to participate in research of this type, we designed a feasibility study to address some of the outstanding questions.

## Methods and analysis

### Primary objectives

The primary objective of this study is to determine the feasibility and acceptability of administering encapsulated FMT to participants colonised with ESBL-E/CPE. This will be used to determine if a substantive trial is feasible.

### Primary endpoints

The primary outcome measure is consent rate (as a proportion of patients who fulfil inclusion/exclusion criteria). The success criteria for the primary endpoint are stratified. If <15% is achieved, progression to a substantive trial will not be deemed feasible. If 15%–39%, progression to a substantive trial will be deemed feasible with protocol modifications and clearly defined stop/go criteria. An overall consent rate of >40% will be taken as indicating a substantive trial is feasible.

### Secondary objectives

The secondary objectives are to assess other feasibility aspects of conducting a substantive trial, to evaluate the safety and tolerability of FMT in this patient population and to provide early evidence of efficacy. These measures should inform a future trial, such as determining the primary (efficacy) outcome and sample size, if progression criteria are met. A full list of criteria for progression to a substantive trial is detailed in online supplementary table 1.

10.1136/bmjopen-2020-038847.supp1 Supplementary data

### Secondary feasibility endpoints

- Proportion of patients fulfilling inclusion/exclusion criteria.
- Proportion of patients receiving FMT/placebo (as a % of those consenting).
- Proportion of patients returning for follow-up visits (face-to-face visit at day 40).
- Proportion of patients providing follow-up stool samples (days 10, 40, 100 and 190).
- Ability to recruit sufficient healthy donors to manufacture all FMT doses to meet demands of this and a future substantive randomised controlled trial (RCT). Assessed by delay in dosing patients (measured in days).

### Additional feasibility assessments will include the following:

- Collection of data that may be used in estimating of costs/resources needed to provide FMT in the National Health Service (NHS).
- An embedded qualitative study to explore views and experiences of research participants.

### Secondary efficacy endpoints

- Gastrointestinal carriage of CRE/ESBL-E (detected/not detected) by stool culture over time (days 10, 40, 100 and 190).
- Gastrointestinal carriage of CRE/ESBL-E (detected/not detected) by multiplex PCR over time (days 10, 40, 100 and 190).

### Secondary safety and tolerability endpoints

- Proportion of patients experiencing reflux following administration of FMT.
- Proportion of patients suffering intolerable (resulting in withdrawal from the study) gastrointestinal side effects (including diarrhoea, constipation, abdominal pain, flatulence and bloating). This will be assessed by direct questioning and completion of a short patient questionnaire.
- Identification of unanticipated harms involved with administration of FMT.
- Occurrence of any adverse event/serious adverse event.

### Exploratory endpoints/outcomes

The following exploratory/mechanistic outcomes will be measured:

- Changes in the gut microbiome induced by capsulised FMT as measured by comparing between treatment groups change (relative to baseline) in the following.
  - The proportion and relative abundance of bacterial taxa over time (days 10, 40, 100 and 190).
  - The change in diversity of the microbiome over time (days 10, 40, 100 and 190) measured using Shannon and Simpson indices.
  - Antibiotic resistance genes carriage over time (days 10, 40, 100 and 190)
- Changes in the gut metabolome induced by capsulised FMT (using nuclear magnetic resonance spectroscopy). Measured at days 10, 40, 100 and 190.
- Host immune response (T and B cells) as measured by comparing participants prior to and day 40, as well as donors who will act as controls.

### Trial design

**Figure 1:**
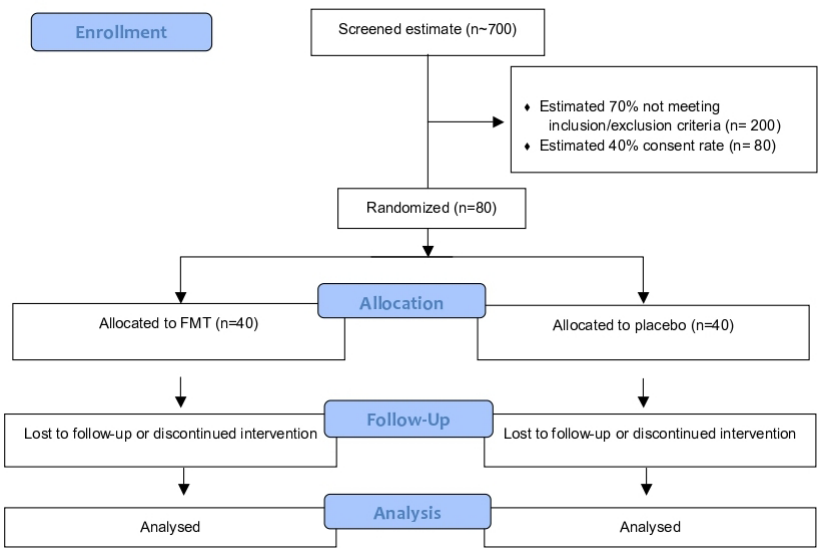
Consolidated Standards of Reporting Trials flow diagram. FMT, faecal microbiota transplant.

Randomised control participant-blinded, single-centre, feasibility trial with two parallel groups (FMT capsules and matched placebo). Eighty patients will be randomised 1:1 (40 will receive FMT capsules and 40 placebo) from eligible patients identified from Guy’s and St Thomas’ hospitals (figure 1 and box 1).

Box 1 **Participant inclusion/exclusion criteria**

**Inclusion criteria**

To be eligible for enrolment, a participant must meet all the following criteria before undergoing any study-related procedures:

- Adult patients (age 18 years or older at time of consent).
- Current/previous patient at Guy’s and St Thomas’ NHS Foundation Trust.
- Ability to understand the purpose, potential benefits and risks of the study and capable of giving informed consent. The participant must be able to provide written informed consent.
- Documented gastrointestinal carriage of ESBL-E or CPE (stool sample) in the 21 days prior to consent.
- Symptomatic infection with the same target organism of interest in preceding 6 months (this needs to be microbiologically confirmed but is not restricted to any particular body site for example, could be urinary tract infection, intra-abdominal infection, blood stream infection).

**Exclusion criteria**

- Pregnancy or planned pregnancy.
- Breastfeeding.
- Severe or life-threatening food allergy.
- Allergy or other contraindication to omeprazole, investigational medicinal product (IMP) or placebo ingredients.
- Treatment with systemic antibiotic on the day of and day prior to first IMP/placebo dosing to the end of the dosing period.
- Treatment with pre or probiotics in the 4 weeks prior to randomisation and for the duration of the study.
- Severe immunodeficiency.

- Systemic chemotherapy <30 days from baseline or planned chemotherapy within the upcoming 6 months.
- Known HIV infection with CD4 count <250 cells/uL.
- Known neutropenia with absolute neutrophils <1.0×10^9^.
- Prolonged treatment with corticosteroids (equivalent to prednisone >60 mg daily for >30 days) within 8 weeks of randomisation.

- Life expectancy <6 months.
- Swallowing disorder, oral-motor dyscoordination or likely inability/unwillingness to ingest study medication.
- Patients who have received another investigational drug or device within 4 months prior to randomisation.
- Any condition or circumstance, in the opinion of the investigator, that would compromise the safety of the patient or the quality of the study data.

### Patient and public involvement

Patients and the public have identified AMR as a research priority and were involved in identifying the research question and providing feedback on the grant application. A patient representative has been appointed to the Trial Steering Committee (TSC), and has advised on the design of the research, the protocol and all patient facing materials. The patient representative will also be involved in dissemination of the study findings. As the acceptability of FMT in this setting is a key research question, we will invite up to eight patients to participate in focus groups. The aim of the group will be to understand their experience in participation in the study and will focus on acceptability, barriers to participation and improvements that could be made to any resulting substantive trial.

### Patient population

Participants will be recruited from Guy’s and St Thomas’ NHS Foundation Trust, a 1200 bed academic centre in central London. It is anticipated that most patients will already be admitted to the hospital as part of standard of care treatment, thus, most activities will take place on the ward or clinical area that the patient is already located. Where this is not the case, participants will be invited to attend the infection clinical room on an outpatient basis.

### Consent

Informed consent (for both healthy donors and patient-recipients) will be obtained prior to any trial related activities, including screening for eligibility. Potential participants will be given the participant information sheet and allowed enough time to read thoroughly and discuss with others outside the study team (eg, family, friends and general practitioner) (see online supplementary table 2). Participants are free to withdraw from the trial at any time without giving reasons. Data and samples collected up to the point of withdrawal will only be used after withdrawal if the participant consented for this. Patients who lack capacity will not be enrolled in this study. Where a participant consents but later becomes incapacitated, the original consent given endures the loss of capacity, providing that the trial has not significantly altered.

### Randomisation

The randomisation schedule will be generated using a validated online randomisation programme, hosted by King’s Clinical Trials Unit. The method of randomisation will be block randomisation with randomly varying block sizes. As this is a single-centre study, randomisation does not need to be stratified. Participants will be allocated treatment as close as possible to receiving it.

### Study intervention

FMT for this trial will be prepared in a lyophilised, encapsulated form in accordance with Good Manufacturing Practice principles and under manufacturing authorisation for an IMP from the Medicines and Healthcare Products Regulatory Agency. Our centre has recently provided FMT for a Clinical Trial of an Investigaional Medicinal Product (CTIMP) for cirrhosis and this follows similar processes.^20^ Healthy donor inclusion and exclusion criteria and screening and eligibility questionnaire are described in online supplementary table 3 and 4.

The product contains 0.9% sodium chloride and 5% trehalose (cryoprotectant) as excipients. A minimum of 80 g faeces from each donor will be used to manufacture one batch of five capsules. Following lyophilisation, the material will be encapsulated in five size 0 delayed release methylcellulose capsules (DRcaps, Capsugel, Livingston, UK). Placebo capsules will contain microcrystalline cellulose. The capsules for the FMT and placebo will be identical in appearance. The capsules are coloured Swedish orange, resulting in an opaque appearance through which the contents cannot be seen.

FMT donors are carefully screened healthy volunteers with a body mass index between 18 and 30. Donors undergo questionnaire screening for risk factors and testing for a range of infectious agents as previously described and in accordance with national guidelines (see appendix A for full details).^21^ FMT material is traceable from donor to recipient. Aliquots of donor stool will be kept for 30 years to allow for future testing if required.

At baseline, participants will have their medication history recorded, including over the counter preparations and supplements as well as pre/probiotics. Vital signs, height and weight and baseline blood biochemistry and haematology will be collected. Additionally, a serum sample will be stored to allow future testing in the event of a possible transmission event. If female and of childbearing age, a urinary pregnancy test will be performed. An EQ-5D questionnaire will also be administered.

Encapsulated FMT (IMP) and placebo will be dispensed by study staff to trial participants over 3 consecutive days (or over 5 days if over a weekend). Patients will be fasted for 4 hours and be pretreated with omeprazole on the morning on the FMT (40 mg on first dosing day and 20 mg on the 2 subsequent dosing days).

It is anticipated that most patients will remain an inpatient for the duration of treatment. If they have been discharged in the interim, provision will be made for them to attend for treatment as an outpatient.

### Evaluations during and after treatment

**Figure 2:**
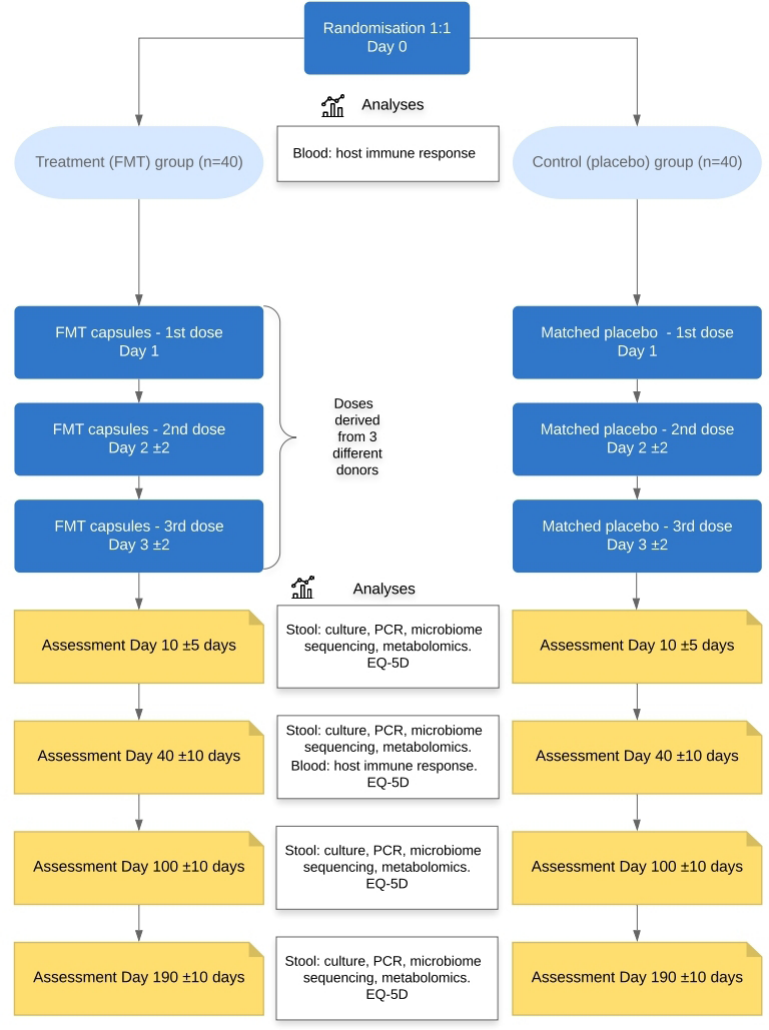
Intervention and follow-up. FMT, faecal microbiota transplant.

Follow-up events will be scheduled for all participants at 1 week, 1 month, 3 months and 6 months after end of therapy. If an outpatient, the visits at 1 week, 3 months and 6 months will be conducted by telephone, with the participant returning a stool sample by post. The follow-up at 1 month will be face to face and will include a blood sample for immune analyses. All visits will involve completion of an EQ-5D questionnaire (see figure 2 for additional details).

### Sample analyses

Stool samples will be analysed for the presence of ESBL-E/CPE using culture based (chromogenic agar with species identification using MALDI-ToF mass spectrometry and phenotypic antimicrobial susceptibility testing) and molecular techniques (multiplex PCR panel for 16 ESBL/CPE resistance genes).

### Follow-up

If a participant fails to present for follow-up assessment, all attempts to contact the participant and information received during contact attempts will be documented in the participant’s medical record. In any circumstance, every effort will be made to contact the participant and document outcome (ie, three documented contact attempts via phone calls, on separate occasions will be made to locate or contact the participant, and/or determine health status). Stool samples will be stored for further follow-on analysis, including metagenomics and metabolomics profiling.

### Qualitative study

A qualitative study of participant’s experiences will be undertaken and comprises a focus group interview with a minimum of eight participants. Ideally, the group will include at least two patients who were approached but did not agree to participate. The aim of these discussions is to identify facilitators and barriers to delivering the trial, and whether there are any aspects of the trial that should be changed. The interviews will be semistructured and recorded to aid writing up the study report. Objectives of the focus group will include identifying ways of increasing recruitment and retention; identifying ways of broadening participation in the trial to improve diversity of population; improving understanding of how participants join trials and experience of participation; measuring reasons for non-adherence to the trial medication; exploring stakeholders’ views of acceptability of the trial design; strengthening the ethical conduct of the trial, for example, informed consent procedures; addressing any local issues which may impact on the feasibility of a substantive trial.

## Statistical analysis

### Sample size

As this is a feasibility study, significance tests between or within groups will not be performed for the study’s primary and secondary endpoints, therefore a power calculation has not been performed. For feasibility and pilot studies, sample sizes between 24 and 50 have been recommended to estimate a chosen parameter.^22 23^ We have chosen a 1:1 treatment to placebo ratio, therefore a total sample size of 80 would be enough to estimate the SD of the outcome in 40 treated patients, allowing for some loss to follow-up. We will also be able to estimate our expected recruitment rate of 40% (95% CI: 33 to 47) if we approach around 200 eligible patients.

### Data synthesis, analysis and presentation

A statistical analysis plan will be written by the trial statistician and signed off prior to database lock. The study will be reported in accordance with the Consolidated Standards of Reporting Trials extension for pilot and feasibility studies.

The proportion of patients who accept the offer of randomisation will be reported with 95% CIs computed by the exact binomial method. No statistical tests for significant differences between treatment groups will be performed. In addition to summary statistics of the secondary outcomes, all harms and withdrawals will be reported with 95% CIs. Patients will be analysed in the groups to which they are randomised in accordance with intent to treat principals.

The protocol has been designed to place minimal burden on patients and case report forms are only capturing essential data. It is inevitable that there may be some missing data, which will be reported by treatment group with reasons for missingness described, where possible. Since this is a feasibility study, we do not plan to impute missing data.

### Statistical software

All statistical analysis will be conducted using Stata V.15.0 or above (StatCorp, Texas).

### Trial monitoring groups

#### Trial management group

Comprises the chief investigator (CI), trial statistician, trial staff and other lead clinical and non-clinical coinvestigators and coapplicants. The TMG are responsible for the day-to-day management of the trial and to ensure that all practical details of the trial are progressing and working well. The TMG will monitor all aspects of the conduct and progress of the trial, ensuring that the protocol is adhered to and take appropriate action to safeguard participants and the quality of the trial itself. The TMG will be responsible for drafting of the final report and submission for publication.

#### Trial Steering Committee

A TSC will be convened with membership nominated by the CI in partnership with the sponsor. The role of the TSC is to provide overall supervision for the trial on behalf of the sponsor and funder and to ensure that the project is conducted to the rigorous standards set out in the Department of Health and Social Care’s Research Governance Framework for Health and Social Care and the Guidelines for Good Clinical Practice. The committee Chair will be independent of the study. The committee will also comprise four other independent members (Consultant Microbiologists or Gastroenterologists), a patient/public representative and an independent statistician.

The TSC will take responsibility for monitoring data and making recommendations to the TMG on whether there are any ethical or safety reasons why the trial should not continue. A separate Data Monitoring and Ethics Committee will not be established as this is a single-centre feasibility trial with a relatively small number of patients using an established IMP with a relatively well described safety profile.

## Discussion

Several case reports and one RCT of ARB decolonisation using FMT are summarised in four systematic reviews.^16 24–26^ Most studies were case reports or case series which did not control for spontaneous loss of ARB carriage. This is important since it may be significant and may lead to overestimation of the effectiveness of FMT in achieving decolonisation. In a recent study conducted at Central Manchester Foundation Trust during 2016/2017, only 17.1% of patients who were previously known to be colonised with CRE had it detected on readmission to the hospital.^27^ Therefore, the use of a placebo in this trial is justified and crucial to control for spontaneous loss of carriage.

Capsule administration has been selected following consultation with patient groups. It is more acceptable and cost effective than other methods of administration such as via nasojejunal tube.

Although the underlying mechanism of action of FMT is not fully elucidated, the use of three different donors is justified as it likely increases the bacterial diversity in the administered IMP, with the hope that this will engraft in the recipient. The previous study using a single donor resulted in an OR for decolonisation success of 1.7 (95% CI 0.4 to 6.4). We hypothesise that using multiple donors at three dosing points will result in a higher rate of decolonisation. This is also based on experience of using FMT to treat patients with ulcerative colitis, where multiple donors are used in prolonged treatment intervals of up to 6 weeks.^28–31^

The overall aim of this programme of work (which would proceed to a future substantive RCT if feasible) is to eradicate or suppress ESBL-E/CRE without resorting to the use of antibiotics. If that can be achieved, then the risk of an invasive infection with ARB in these patients could be significantly reduced.

## Supplementary Material

Reviewer comments

Author's manuscript
