# Supplementary material for: Faecal microbiota transplant to ERadicate gastrointestinal carriage of Antibiotic Resistant Organisms (FERARO): a prospective, randomised placebo-controlled feasibility trial
Source: BMJ Open. 2020 May 25;10(5):e038847. doi: 10.1136/bmjopen-2020-038847 (PMC7252984; doi:10.1136/bmjopen-2020-038847)
Supplement: Supplementary data [file bmjopen-2020-038847supp001.pdf]

Supplementary file S1.

FERARO: A prospective, randomised placebo-controlled feasibility trial of **F**aecal microbiota Transplant to **ER**adicate gastrointestinal carriage of **A**ntibiotic **R**esistant **O**rganisms: study protocol for single-blinded trial

Table 1. Criteria for progression to a substantive trial

Criteria	Stop – substantive trial not feasible	Continue with protocol modifications, close monitoring and clearly defined stop/go points	Continue without modification – feasible as is
**Hard Feasibility Criteria<br>Used to determine progression to a substantive trial**			
Consent rate (% of patients who fulfil eligibility criteria)	<15%	15-39%	>40%
**Soft Feasibility Criteria<br>Taken into consideration in determining progression to a substantive trial**			
Proportion of patients fulfilling eligibility criteria	<10%	10-29%	>30%
% of patients receiving IMP/placebo (as % of those consenting) and compliant will all doses on all three days	<50%	50-75%	>75%
% of patients returning for follow up visit (Day 40)	<50%	50-79%	>80%
% of patients providing follow up stool samples (Days 10, 40, 100 and 190)	<50% returning two or more samples	50-79% patients returning two or more samples	>80% patients returning two or more samples
Ability to recruit sufficient healthy donors to manufacture all FMT	Delay in dosing patients >2 weeks	Delay in dosing patients up to 2 week2	No delay in patient dosing
**Soft Patient Tolerability Criteria<br>Taken into consideration in determining progression to a substantive trial**			
% of patients experiencing reflux	>51%	21-50%	<20 %

Merrick B, *et al. BMJ Open* 2020; 10:e038847. doi: 10.1136/bmjopen-2020-038847

following FMT administration			
Intolerable (resulting in withdrawal) side effects	>51%	21-50%	<20 %

Table 2. Participant Information Sheet (PIS)

A prospective, randomised placebo controlled feasibility trial of **F**aecal microbiota Transplant to **ER**adicate gastrointestinal carriage of **A**ntibiotic **R**esistant **O**rganisms

The FERARO Trial

Patient Information Sheet

We would like to invite you to take part in the FERARO trial.

- Before you decide whether you would like to take part in the trial, it's important for you to understand why the research is being done and what it would involve.
- Please take some time to read the information carefully, and discuss with your family, friends and doctor, as you wish.
- Ask us if anything is not clear, or you would like some more information

Important things that you need to know:

- Taking part is completely up to you and you can stop taking part at any time, without giving a reason. If you do not wish to take part, this will not affect the care you receive from your doctors or other health care professionals.
- You have been asked to take part in this study because a sample you have provided contains Antibiotic Resistant Bacteria (ARB). This study is testing the acceptability of a treatment that may be able to reduce the amount of ARB in the gut and the risk of infection.
- Faecal Microbiota Transplant (FMT) is a capsule made up of bacteria taken from a stool (poo) sample donated by healthy people. It could help to restore the balance of bacteria in the gut by reducing the amount of bacteria that are resistant to antibiotics.
- Faecal Microbiota Transplant (FMT) is a treatment used currently to treat patients with repeated Clostridioides difficile (C. diff) infection.
- The FERARO trial is looking to see if you consider this to be an acceptable treatment and if there are any side effects.

- You will be allocated to receive either FMT capsules or a placebo (dummy) capsules. You will not know which treatment you receive.
- You will be asked to take five capsules a day for three days in a row. This may be while you are already staying in hospital or as an outpatient.
- You will be asked to provide stool samples before and after receiving treatment to see if there are any changes in the bacteria present in your gut.
- You will be followed up by the study team for six months after completing the treatment.

If you have any questions about this study, please contact:

Dr Simon Goldenberg
Telephone (with message facility): 020 7188 8515
Email: simon.goldenberg@gstt.nhs.uk

What is the purpose of the study?

The human gut has trillions of good bacteria (germs or bugs) which are important to keep us healthy. In total these bugs are called the microbiota. The bugs are always evolving to beat antibiotics used to fight them and this is known as resistance. Resistance to antibiotics allows bugs to survive and spread.

Antibiotic resistant bacteria (ARB) usually live in the gut (or in the surrounding environment), where they do no harm. This is called colonisation. However, the ARB can appear and cause infection in other parts of the body that normally lack any bacteria, for example in the bladder or blood. When this happens, treatment with a more powerful type of antibiotic is usually needed. This is more likely to happen in people who are more prone to infection, including people with an underlying disease or injury, or people who are already admitted to hospital.

Antibiotic resistance is a growing and serious threat to worldwide health, and means that doctors may be limited in the types of treatments that they can offer to patients. Without effective antibiotics even simple infections could become deadly, making routine medical procedures too dangerous to perform. There is an urgent need to find new antibiotics, but this takes time and is very expensive.

Two particular groups of antibiotic resistant bacteria are known as CRE and ESBL (Carbapenem Resistant Enterobacteriales and Extended Spectrum Beta-lactamase producing bacteria).

Carbapenems and beta-lactams are some of the most powerful types of antibiotics. Some strains of bacteria make enzymes (chemicals), which allow them to destroy carbapenem and beta-lactam antibiotics which makes the bacteria resistant to the antibiotics.

Merrick B, *et al. BMJ Open* 2020; 10:e038847. doi: 10.1136/bmjopen-2020-038847

There is growing interest in non-antibiotic treatments like Faecal Microbiota Transplant (FMT) to deal with this problem.

FMT is the transfer of bacteria from the guts of healthy donors (taken from their poo / stools) into the gut of a patient. The aim is to restore a healthy balance of bacteria (reducing harmful ones and increasing good ones). It is currently used to treat patients with repeated Clostridioides difficile infection. This is an infection causing severe diarrhoea and stomach pain, normally after having antibiotics which have harmed the microbiota.

FMT is very effective and safe in treating this group of patients, with success rates of over 80%. Initial research shows that it may be helpful in other conditions.

Why have I been invited to take part?

You have been identified by a member of your healthcare team as a carrier of ARB. You have had an infection caused by ARB in the last 6 months. This might be a urine, bloodstream, chest or other type of infection and may have been on a previous visit to the hospital or from a sample that your General Practitioner sent to our lab.

Do I have to take part?

No, participation in the study is entirely voluntary. It is up to you whether or not you wish to take part. You will be given time to read the patient information sheet and ask any questions you wish about the study.

If you decide to take part you will be given this information sheet to keep and asked to sign a consent form. If you decide to take part you are still free to withdraw at any time and without giving a reason.

What will happen if I take part?

You will be asked to provide a stool sample to first check for the presence of the ESBL or CRE bacteria. If we don't find the bacteria you will not be able to continue in the study and any samples that you have already provided will be destroyed.

If ESBL or CRE bacteria are detected, you will be asked to provide a blood sample and female participants may be asked to provide a urine sample for a pregnancy test. Once we can confirm that it is safe for you to enter the study, you will be placed in one of two groups. This will be decided by chance. One group will receive FMT treatment, and the other will receive placebo (dummy) capsules. Both groups will be given five identical capsules to take each day for three days in a row. You will not know which group you are in until the end of the study.

Before taking the trial treatment, you will be asked to fast for 4 hours. You cannot eat any food during this time but you can drink water. You will also be asked to take a commonly prescribed medication called omeprazole. This works to reduce the amount of acid in the stomach. It will help protect the good bacteria in the capsules from being damaged.

Merrick B, *et al. BMJ Open* 2020; 10:e038847. doi: 10.1136/bmjopen-2020-038847

You will then take all 5 capsules with water or squash. A member of the study team will stay with you for a short time while you take the capsules and check for any side effects. You can eat and drink as normal shortly after taking the capsules. The capsules will only be given on weekdays. If you are already staying in hospital, you will receive the treatment during your stay. If you are allowed to go home during this time, we will ask you to return to the hospital to complete the treatment. If you are not currently admitted to hospital, you will be asked to visit the hospital as an outpatient to complete the treatment.

You will be contacted by the study team by telephone after completing the treatment. This will happen 1 week, 3 months and 6 months after receiving treatment. You will be asked to provide a stool sample using the collection kit provided, and send it back to us in the post (postage will be pre-paid). You will be asked to complete a quality of life questionnaire over the phone. We will ask you how you have been feeling and if you are taking any new medications.

We also ask that you return to St Thomas' Hospital for a visit about 1 month after taking the capsules and we will ask you to bring a stool sample at that visit. We are able to reimburse you for the travel costs of visits to the hospital needed for the study, up to a maximum of £20 per visit.

In total you will be asked to provide five stool samples.

	Assessment	Treatment Day 1	Treatment Day 2	Treatment Day 3	Follow Up 1 week, 3 months, 6 months	Follow Up 1 month
	During Hospital Stay or Visit 1	During Hospital Stay or Visit 2	During Hospital Stay or Visit 3	During Hospital Stay or Visit 4	Telephone Call	Hospital Visit
Consent form	X					
Blood sample	X					X
Stool sample	X				X	X
Pregnancy test (females)	X					
Eligibility Check	X					
Basic health check	X	X	X	X		
Quality of Life Questionnaire	X				X	X
Fasting for 4 hours		X	X	X		
Capsule administration		X	X	X		
Side effects and medication check		X	X	X	X	X

With your consent we will send a letter to your GP to let them know that you are taking part in the trial.

What will happen to any samples I provide?

Stool samples will be processed in the FMT laboratory at St Thomas's Hospital and divided up into smaller samples. Some of the sample will be analysed at St Thomas' Hospital and some will be sent to KCL for analysis. They will be labelled with a study ID number that links the samples to who you are. This link will be kept securely by the study team and will not be shared with anybody else. No directly identifiable information will be used to label your samples.

You will be asked to provide 14 ml of blood (equivalent to 3 teaspoons) before entering the study. This is to check that it is safe for you to enter the study. If any of these tests have already been done as part of your normal care in the previous 5 days, we will not need to repeat them.

Will my samples be used in future research?

We will ask for your consent to take a blood sample and to use the stool samples that you have already provided for use in future research in addition to the samples mentioned above. The consent for storage of samples for future research is optional and will not affect your participation in the study in any way. If you do consent to this, we will ask you for 40 ml (8 teaspoons) before receiving the study treatment. This will be taken at the same time as the initial blood sample, so doesn't require an extra needle. We will also ask for a further 40 ml (8 teaspoons) at the face to face visit about 1 month after taking the capsules.

What are the possible benefits and disadvantages of taking part?

This study is designed to look at the safety and tolerability of FMT treatment to see if a larger study would show an improvement in wellbeing for patients with ARB infections. There are few treatments available to patients with antimicrobial resistant infections.

We hope that the knowledge we would gain from this study will improve our understanding of the way in which FMT works, and the role of the treatment in antimicrobial resistance. FMT appears to be safe in the considerable numbers of patients who have received it for other reasons.

If you are in the group that receives FMT, it is possible that it will help to reduce the ARB in found in your gut and reduce the risk of further infections. However, we will not know this until we have completed this study and future studies. You may not directly benefit from taking part in this study, but the information gained may help to improve the treatment of people with your condition in the future.

If you decide to take part in the study, you may be asked to attend the hospital more frequently than you would if you choose not to take part. You will also receive more regular input from a study doctor and nurse to monitor how you are feeling after the treatment.

What is the drug that is being tested?

Merrick B, *et al. BMJ Open* 2020; 10:e038847. doi: 10.1136/bmjopen-2020-038847

FMT is the transfer of bacteria from the guts of healthy donors (taken from their poo) into the gut of a patient. FMT is produced by carefully selecting healthy individuals who have undergone an extensive health assessment and have been tested for a wide range of infections and other diseases. We follow National guidelines when selecting volunteer donors. The donor poo sample is processed in the laboratory to concentrate the healthy bacteria and remove most of the water (freeze drying) which leaves a small amount of powder. The powder is then packed into capsules which need to be swallowed.

FMT is currently used to treat patients with repeated Clostridioides difficile infection. In these patients, FMT is administered via a tube into the stomach or the lower bowel.

FMT is very effective and safe in treating this group of patients, with success rates of over 80%.

Some patients have experienced side effects, including belching, abdominal cramps and abdominal pain. Diarrhoea and constipation has also been reported.

We hope that producing FMT in capsules will help to minimise any side effects. We will monitor you closely while you are taking FMT and ask how you are feeling after receiving the treatment.

We would like you to report any unexpected symptoms or health events to the study team, even if you think it is not related to the treatment.

Omeprazole

Omeprazole reduces the amount of acid your stomach makes. It's a widely used treatment for indigestion, heartburn and acid reflux.

Most people who take omeprazole don't have any side effects, particularly if it is taken over a short period of time, as is the case here. If you do get a side effect, it's usually mild (such as abdominal pain, constipation, diarrhoea, dizziness and dry mouth) and will go away when you stop taking omeprazole.

How is FMT made?

After the healthy volunteers have been carefully screened and tested, the stool sample is brought to the FMT laboratory. Sterile salt water will be added to the stool and it will be filtered to remove any solid material and then be freeze-dried to remove water.

The resultant material is placed into capsules and stored frozen until it is required by the person who will receive it. Once a patient has been identified and consented to take part in the FERARO study, the capsules will be administered to the patient.

We will store a small amount of the stool sample to assess the types of bacteria present (microbiota analysis) so that we can compare to the stool samples provided by the patients that take the FMT.

These analyses will be performed at King's College, London for the blood samples. As part of a separate study the stool sample analysis will be performed at the National Institute for Biological Standards and Controls.

All samples will be completely anonymised. Patients who receive the FMT will not be given any information about who donated the samples.

Stool will be archived for thirty years for traceability, so that in the unlikely event of an infection occurring in the recipient the donor stool can be checked for infection also. The data will be anonymised and will only be accessible to members of the trials team. After this period it will be destroyed.

Who is organising and funding this study?

The doctor in charge of this study is Dr Simon Goldenberg. The study is funded by the National Institute of Health Research and is being sponsored by Guy's and St Thomas' NHS Foundation Trust.

Who has reviewed the study?

All research in the NHS is looked at by an independent group of people, called a Research Ethics Committee, to protect your interests. This study has been reviewed and approved by the London – City and East Research Ethics committee (reference 20/LO/0117.) It has also been reviewed by an independent review group and approved by the Health Research authority and the Medicines and Healthcare products Regulatory Agency.

What if something goes wrong?

If you have a concern about any aspect of this study, you should ask to speak to your study doctor who will do their best to answer your questions (contact details on page 2 of this information sheet).

If you remain unhappy and wish to complain formally, you can do this through the NHS Complaints procedure by contacting the Patient Advice Liaison Service (PALS) office.

Guy's and St Thomas' NHS Foundation Trust PALS
Contact number: 0207 188 8801 or pals@gstt.nhs.uk

Every care will be taken in the course of this study. However in the unlikely event that you are injured by taking part, compensation may be available.

In the event that something does go wrong and you are harmed during the research and this is due to someone's negligence then you may have grounds for a legal action for compensation against Guy's and St. Thomas' NHS Foundation Trust, but you may have to pay your legal costs.

How will we use information about you?

We will need to use information from you and from your medical records for this research project. This information will include your hospital number, name, date of birth, address and contact details. People will use this information to do the research or to check your records to make sure that the research is being done properly.

People who do not need to know who you are will not be able to see your name or contact details. Your data will have a code number instead. We will keep all information about you safe and secure.

Once we have finished the study, we will keep some of the data so we can check the results. We will write our reports in a way that no-one can work out that you took part in the study.

What are your choices about how your information is used?

You can stop being part of the study at any time, without giving a reason, but we will keep information about you that we already have.

If you agree to take part in this study, you will have the option to take part in future research using your data saved from this study.

Where can you find out more about how your information is used?

You can find out more about how we use your information at:
www.guysandstthomas.nhs.uk/research/patients/use-of-data.aspx
at www.hra.nhs.uk/information-about-patients/
by asking one of the research team or by sending an email to the Chief Investigator
simon.goldenberg@gstt.nhs.uk

What will happen to the results of the research study?

Once the data has been analysed we plan to publish the results in an international journal so that the information can benefit as many people as possible. We can provide you with a brief summary of the results of the study when available should you desire this. If you would like to be kept informed about the results of the study, please email the Chief Investigator using the details below.

Contact details:

Thank you for taking the time to read this information. If there is any other information you would like, please do not hesitate to contact the study team on the numbers below.

Study co-ordinator:	Dr Blair Merrick	Tel: 0207 188 7188 extension 53339
Research Nurse:	Karen Bisnauthsing	Tel: 0207 188 7188 extension 53339
Chief Investigator:	Dr Simon Goldenberg	Tel: 0207 188 8515

Table 3. Healthy donor inclusion and exclusion criteria

Inclusion criteria

- 18-60 years of age **AND**
- BMI 18-30

Exclusion criteria

- Received antimicrobials within past 3 months
- Known prior exposure to HIV and/or viral hepatitis
- Known previous or latent tuberculosis
- Risk factors for blood borne viruses within the previous 6 months
- Received live attenuated vaccine within past 6 months
- Underlying gastrointestinal condition/ or unexplained symptoms including acute diarrhoea in 2 weeks prior to donating
- Family history of any significant gastrointestinal condition
- History of atopy, systemic autoimmune condition, diabetes, neurological or psychiatric condition or known risk of prion disease. History of chronic pain syndrome including chronic fatigue and fibromyalgia, or malignancy.
- Taking any regular medications
- History of taking proton pump inhibitors or immunosuppressive medications within the past 3 months
- Ever received growth hormone, insulin from cows or clotting factor concentrates
- Received an experimental medicine or vaccine within the past 6 months
- Travelled to a tropical country within the past 6 months

Merrick B, *et al. BMJ Open* 2020; 10:e038847. doi: 10.1136/bmjopen-2020-038847

Table 4. Donor screening and eligibility questionnaire

Guy's and St Thomas' NHS
NHS Foundation Trust

Faecal Microbiota Transplant (FMT) –Donor programme

Donor name	
Donor date of birth	
Donor Hospital number	
Donor number	
Donor contact details – telephone / email	
Name of assessor	
Position	
Date of assessment	
Age	Exclude if <18 or >60
Gender	☐ Male ☐ Female
Ethnicity	☐ White - White British<br>☐ White - White Irish<br>☐ White - Other<br>☐ Mixed race – White and Black Caribbean<br>☐ Mixed race – White and Black African<br>☐ Mixed race – White and Asian<br>☐ Mixed race – Other<br>☐ Asian or Asian British – Indian<br>☐ Asian or Asian British – Bangladeshi<br>☐ Asian or Asian British – Pakistani<br>☐ Asian or Asian British – Other<br>☐ Black or Black British – Caribbean<br>☐ Black or Black British – African<br>☐ Black or Black British – Other<br>☐ Chinese<br>☐ Other
Height	cm
Weight	

	kg
BMI	Exclude if BMI ≤18 or ≥30
Has your weight changed by more than 5lb / 2kg in the past 6 months?	☐ Yes ☐ No<br><br>Detail:
Describe your diet (as many as apply):	☐ Omnivore<br>☐ Vegetarian<br>☐ Vegan<br>☐ Kosher<br>☐ Halal<br>☐ Raw food only<br>☐ Pescatarian<br>☐ No red meat<br>☐ Low carbohydrate<br>☐ Lactose free<br>☐ Gluten free<br>☐ Other
How many portions of fruit and vegetables do you consume per day?	☐ one or less<br>☐ two to three<br>☐ three to four<br>☐ five to six<br>☐ seven or more
How many servings of cow, sheep or goat's milk do you consume per day?	☐ one or less<br>☐ two to three<br>☐ three to four<br>☐ five to six<br>☐ seven or more
Alcohol – units/week	
Smoking/day	
Do you take any illicit drugs?	☐ Yes ☐ No<br><br>Exclude if YES
Normal bowel habit – average Bristol Stool Consistency	☐ 1<br>☐ 2<br>☐ 3<br>☐ 4<br>☐ 5<br>☐ 6<br>☐ 7<br><br>Exclude if 1, 6 or 7

Normal bowel habit – average frequency	☐ >2/day<br>☐ once to twice daily<br>☐ once / 2 days<br>☐ <once / 2 days<br><br>Exclude if active diarrhoea (>3 UBM/day for at least 2 consecutive days)
Have you ever been rejected as a blood donor/told not to donate?<br>If yes, why?	☐ Yes ☐ No<br><br>Exclude if YES
What is your country of birth?	
Have you ever resided in another country (other than UK) for >5 years?<br>If so which countries and when?	☐ Yes ☐ No
Do you currently have a profession that is associated with an increased risk of blood-borne transmissible diseases (e.g. daily contact with patients/inmates)?<br>If yes, what profession?	☐ Yes ☐ No<br><br>Exclude if health/social care worker with direct patient contact
Have you ever had a needle-stick or injury from a blood contaminated object from someone else?<br><br>If yes, when and how was this follow up?	☐ Yes ☐ No
Have you ever injected yourself or been injected with illegal or non-prescribed drugs including body building drugs or cosmetics (even if this was only once or a long time ago?)	☐ Yes ☐ No<br><br>Exclude if YES
Have you ever had a tattoo?<br>If yes, when and in which country was it performed?	☐ Yes ☐ No<br><br>Exclude if within past 6 months
Have you ever had a piercing?<br>If yes, when and in which country was it performed?	☐ Yes ☐ No<br><br>Exclude if within past 6 months
Have you ever had acupuncture?<br>If yes, when and in which country was it performed?	☐ Yes ☐ No<br><br>Exclude if within past 4 months
Have you ever had an operation or undergone clinical treatment in	☐ Yes ☐ No

a hospital with poor hygienic conditions?<br>If yes, when and in which country was it performed?	
Have you ever had a rare infectious disease (e.g. tuberculosis, malaria, trypanosomiasis)?<br>If yes, when and which disease?	☐ Yes ☐ No
Have you ever been vaccinated against Hepatitis A or B?<br>If yes, which?	☐ Yes ☐ No
In the last 12 months have you had sex with anyone who is HIV positive?	☐ Yes ☐ No<br>Exclude if Yes
In the last 12 months have you had sex with anyone with hepatitis B, hepatitis C or HTLV?	☐ Yes ☐ No<br>Exclude if Yes
In the last 12 months have you had sex with anyone who has ever been given money or drugs for sex?	☐ Yes ☐ No<br>Exclude if Yes
In the last 12 months have you had sex with anyone who has ever injected drugs?	☐ Yes ☐ No<br>Exclude if Yes
In the last 12 months have you had sex with anyone who may ever have had sex in parts of the world where HIV/AIDS is very common (this includes most countries in Africa)?	☐ Yes ☐ No<br>Exclude if Yes
**Male donors ONLY:**<br>In the last 12 months have you ever had oral or anal sex with a man, with or without a condom?	☐ Yes ☐ No<br>Exclude if Yes
**Female donors ONLY:**<br>In the last 12 months have you had sex with a man who has ever had oral or anal sex with another man, with or without a condom?	☐ Yes ☐ No<br>Exclude if Yes
Have you ever been treated for an intestinal infection?<br>If yes, which one and when?	☐ Yes ☐ No
Do you have any gastro-intestinal conditions:<br>Barretts Oesophagus<br>Coeliac disease<br>Diverticular disease	☐ Yes ☐ No<br>☐ Yes ☐ No<br>☐ Yes ☐ No<br>☐ Yes ☐ No

Merrick B, *et al. BMJ Open* 2020; 10:e038847. doi: 10.1136/bmjopen-2020-038847

Bariatric surgery<br>Gastric ulcer<br>Gasto-oesophageal reflux disease<br>Hepatitis<br>H. pylori infection<br>Crohns disease<br>Ulcerative colitis<br>Other inflammatory bowel diseases<br>Irritable Bowel Syndrome<br>Lactose intolerance<br>Liver disease<br>Pancreatitis<br>Gastrointestinal malignancy or polyps	☐ Yes ☐ No<br>☐ Yes ☐ No<br>☐ Yes ☐ No<br>☐ Yes ☐ No<br>☐ Yes ☐ No<br>☐ Yes ☐ No<br>☐ Yes ☐ No<br>☐ Yes ☐ No<br>☐ Yes ☐ No<br>☐ Yes ☐ No<br>☐ Yes ☐ No<br>☐ Yes ☐ No<br><br>Exclude if Inflammatory Bowel Disease, Irritable Bowel Syndrome, GI malignancy, Hepatitis
Is there any family history of inflammatory Bowel Disease or colorectal cancer?	☐ Yes ☐ No<br><br>Exclude if Yes
Have you taken any antibiotics in the last 3 months?	☐ Yes ☐ No<br><br>Exclude if Yes
Have you had a fever in the last 2 weeks?	☐ Yes ☐ No<br><br>Exclude if Yes
Have you received a live vaccination within the past 6 months?	☐ Yes ☐ No<br><br>Exclude if Yes
Have you ever been incarcerated in prison?	☐ Yes ☐ No<br><br>Exclude if in past 4 months
Have you ever been immunosuppressed (e.g. during treatment for cancer, or for a solid organ transplant)?<br>If yes, when and why?	☐ Yes ☐ No
Have you ever had major gastrointestinal surgery?<br>If yes, when and why?	☐ Yes ☐ No<br><br>Relative exclusion criteria
Have you ever suffered from metabolic syndrome or diabetes?	☐ Yes ☐ No<br><br>Exclude if Yes
Have you ever suffered from any autoimmune condition (e.g. rheumatoid), asthma or eczema?<br>If yes, which, and do you take any treatment?	☐ Yes ☐ No<br><br>Relative exclusion criteria
Have you ever had any chronic pain or fatigue syndromes e.g.	☐ Yes ☐ No

chronic fatigue syndrome, fibromyalgia?<br>If yes, which?	Exclude if Yes
Have you any history of CJD or other prion disease in your family? If yes, please specify<br>Patients should be considered to be at risk from genetic forms of CJD if they have or have had<br>1. Genetic testing, which has indicated they are at significant risk of developing CJD or other prion disease<br>2. A blood relative known to have a genetic mutation indicative of genetic CJD or other prion disease<br>3. Two or more blood relatives affected by CJD or other prion disease	☐ Yes ☐ No<br><br>Exclude if Yes
Have you ever received growth hormone or gonadotrophic treatment? If yes, please specify;<br>i) Whether the hormone was derived from human pituitary glands<br>ii) The year of the treatment<br>iii) Whether the treatment was received in the UK or in another country<br><br>Recipients of hormone derived from human pituitary glands e.g. growth hormone or gonadotrophin have been identified as potentially at risk of CJD. In the UK, the use of human growth hormone was stopped in 1985 but human-derived products may have been continued to be used in other countries. In the UK, the use of human-derived gonadotrophin was discontinued in 1973 but may have been continued in other countries after this time.	☐ Yes ☐ No<br><br>Exclude if Yes

Have you ever had surgery on your brain or spinal cord?<br>People who underwent intradural neurosurgical or spinal procedures before August 1992 may have received a graft of human-derived dura mater and should be treated as at increased risk, unless evidence can be provided that human-derived dura mater was not used. Patients who received a graft of human-derived dura mater between 1980 and August 1992 are at increased risk of both sporadic CJD and vCJD.	☐ Yes ☐ No<br><br>Exclude if Yes
Are you normally resident in the UK?<br>If No state country of usual residence	☐ Yes ☐ No
Which countries have you visited in the last 12 months and what was the duration of stay?	Relative exclusions apply to tropical countries
In the past 12 months have you been admitted to a hospital in a country other than the UK?<br><br>If yes state when and which countries	☐ Yes ☐ No
Are you taking any regular medications?<br><br>List all current medications:	☐ Yes ☐ No<br><br>Exclude if any regular prescribed drugs (except OCP)

