# Supplementary material for: Faecal microbiota transplant to ERadicate gastrointestinal carriage of Antibiotic Resistant Organisms (FERARO): a prospective, randomised placebo-controlled feasibility trial
Source: BMJ Open. 2020 May 25;10(5):e038847. doi: 10.1136/bmjopen-2020-038847 (PMC7252984; doi:10.1136/bmjopen-2020-038847)
Supplement: Author's manuscript [file bmjopen-2020-038847.draft_revisions.pdf]

BMJ Open

BMJ Open is committed to open peer review. As part of this commitment we make the peer review history of every article we publish publicly available.

When an article is published we post the peer reviewers' comments and the authors' responses online. We also post the versions of the paper that were used during peer review. These are the versions that the peer review comments apply to.

The versions of the paper that follow are the versions that were submitted during the peer review process. They are not the versions of record or the final published versions. They should not be cited or distributed as the published version of this manuscript.

BMJ Open is an open access journal and the full, final, typeset and author-corrected version of record of the manuscript is available on our site with no access controls, subscription charges or pay-per-view fees (http://bmjopen.bmj.com).

If you have any questions on BMJ Open's open peer review process please email info.bmjopen@bmj.com

BMJ Open

Protocol: A prospective, randomised placebo-controlled feasibility trial of Faecal microbiota Transplant to ERadicate gastrointestinal carriage of Antibiotic Resistant Organisms (FERARO)

Journal:	*BMJ Open*
Manuscript ID	bmjopen-2020-038847
Article Type:	Protocol
Date Submitted by the Author:	26-Mar-2020
Complete List of Authors:	Merrick, Blair; Guy's and Saint Thomas' Hospitals NHS Trust, Centre for Clinical Infection and Diagnostics Research; King's College London Faculty of Life Sciences and Medicine<br>Robinson, Emily; King's College London, School of Population Health and Environmental Sciences<br>Bunce, Catey; King's College London, Primary Care and Public Health Sciences<br>Allen, Liz; Guy's and Saint Thomas' Hospitals NHS Trust; IQVIA Reading<br>BIsnauthsing, Karen; Guy's and Saint Thomas' Hospitals NHS Trust, Centre for Clinical Infection and Diagnostics Research<br>Izundu, Chi Chi<br>Bell, Jordana; King's College London Faculty of Life Sciences and Medicine<br>Amos, Gregory; National Institute for Biological Standards and Control<br>Shankar-Hari, Manu; Guy's and Saint Thomas' Hospitals NHS Trust, Intensive Care Unit; King's College London Faculty of Life Sciences and Medicine, Division of Asthma, Allergy and Lung Biology<br>Goodman, Anna; Guy's and Saint Thomas' NHS Foundation Trust, Centre for Clinical Infection and Diagnostics Research; King's College London Faculty of Life Sciences and Medicine<br>Shawcross, Debbie ; King's College London Faculty of Life Sciences and Medicine, Hepatology<br>Goldenberg, Simon; Guy's and Saint Thomas' NHS Foundation Trust, Centre for Clinical Infection and Diagnostics Research; King's College London Faculty of Life Sciences and Medicine
Keywords:	BACTERIOLOGY, Microbiology < BASIC SCIENCES, Infection control < INFECTIOUS DISEASES

SCHOLARONE™
Manuscripts

I, the Submitting Author has the right to grant and does grant on behalf of all authors of the Work (as defined in the below author licence), an exclusive licence and/or a non-exclusive licence for contributions from authors who are: i) UK Crown employees; ii) where BMJ has agreed a CC-BY licence shall apply, and/or iii) in accordance with the terms applicable for US Federal Government officers or employees acting as part of their official duties; on a worldwide, perpetual, irrevocable, royalty-free basis to BMJ Publishing Group Ltd ("BMJ") its licensees and where the relevant Journal is co-owned by BMJ to the co-owners of the Journal, to publish the Work in this journal and any other BMJ products and to exploit all rights, as set out in our licence.

The Submitting Author accepts and understands that any supply made under these terms is made by BMJ to the Submitting Author unless you are acting as an employee on behalf of your employer or a postgraduate student of an affiliated institution which is paying any applicable article publishing charge ("APC") for Open Access articles. Where the Submitting Author wishes to make the Work available on an Open Access basis (and intends to pay the relevant APC), the terms of reuse of such Open Access shall be governed by a Creative Commons licence – details of these licences and which Creative Commons licence will apply to this Work are set out in our licence referred to above.

Other than as permitted in any relevant BMJ Author's Self Archiving Policies, I confirm this Work has not been accepted for publication elsewhere, is not being considered for publication elsewhere and does not duplicate material already published. I confirm all authors consent to publication of this Work and authorise the granting of this licence.

For peer review only

Protocol: A prospective, randomised placebo-controlled feasibility trial of **F**aecal microbiota Transplant to **ER**adicate gastrointestinal carriage of **A**ntibiotic **R**esistant **O**rganisms (FERARO)

Blair Merrick[1], Emily Robinson[2], Catey Bunce[2], Liz Allen[3, 4], Karen Bisnauthsing[1], Chi Chi Izundu[5], Jordana Bell[6], Gregory CA Amos[7], Manu Shankar-Hari[8], Anna Goodman[1], Debbie L Shawcross[9], Simon D Goldenberg[1] *

Author affiliations

[1]Centre for Clinical Infection and Diagnostics Research, King's College, London and Guy's & St Thomas' NHS Foundation Trust, London, UK
[2] School of Population Health and Environmental Sciences, King's College London, UK
[3] Early Clinical Development Centre of Excellence, IQVIA, Reading UK
[4] Department of Pharmacy, Guy's and St Thomas' NHS Foundation Trust, London, UK
[5] Patient and Public Representative, London, UK
[6] Department of Twin Research and Genetic Epidemiology, King's College, London, UK
[7] National Institute for Biological Standards and Control, Potters Bar, Hertfordshire, UK
[8] Department of Infectious Diseases, School of Immunology and Microbial Sciences, Faculty of Life Sciences and Medicine, King's College, London and Guy's and St Thomas' NHS Foundation Trust, London, UK
[9] Institute of Liver Studies, Inflammation Biology, School of Immunology and Microbial Sciences, Faculty of Life Sciences and Medicine, King's College London, London, UK
* Corresponding author

Blair.merrick@gstt.nhs.uk
Emily.robinson@kcl.ac.uk
Catey.bunce@kcl.ac.uk
Liz.allen@iqvia.com
Karen.bisnauthsing@gstt.nhs.uk
Chi.chi.izundu@bbc.co.uk
Jordana.bell@kcl.ac.uk
Gregory.amos@nibsc.org
Manu.shankar-hari@kcl.ac.uk
Anna.goodman@gstt.nhs.uk
Debbie.shawcross@kcl.ac.uk
Simon.goldenberg@gstt.nhs.uk

Keywords: antimicrobial resistance, extended spectrum beta-lactamases, carbapenemase producing enterobacteriales, microbiome, faecal microbiota transplantation

Word count: 3949

For peer review only

Abstract

Introduction: antimicrobial resistance (AMR) is rising, largely due to the indiscriminate use of antimicrobials. The human gut is the largest reservoir of antibiotic resistant bacteria (ARB). Individuals colonised with ARB have the potential to spread these organisms both in the community and hospital settings. Infections with ARB such as extended spectrum beta-lactamase producing enterobacteriales (ESBL-E) and carbapenemase producing enterobacteriales (CPE) are more difficult to treat and are associated with an increased morbidity and mortality. Presently there is no effective decolonisation strategy for these ARB. Faecal microbiota transplant (FMT) has emerged as a potential strategy for decolonisation of ARB from the human gut, however there is significant uncertainty about the feasibility, effectiveness and safety of using this approach.

Methods and analysis: prospective, randomised, patient-blinded, placebo-controlled feasibility trial of FMT to eradicate gastrointestinal carriage of ARB. Eighty patients with a recent history of invasive infection secondary to ESBL-E or CPE and persistent gastrointestinal carriage will be randomised 1:1 to receive encapsulated FMT or placebo. The primary outcome measure is consent rate (as a proportion of patients who fulfil inclusion/ exclusion criteria); this will be used to determine if a substantive trial is feasible. Participants will be followed up at 1 week, 1 month, 3 months and 6 months and monitored for adverse events as well as gastrointestinal carriage rates of ARB after intervention.

Ethics and dissemination: research ethics approval was obtained by London - City & East Research Ethics Committee (ref 20/LO/0117).

Trial registration number: ISRCTN registration number 34467677 and EudraCT number 2019-001618-41 protocol version 1.1. (dated 23/02/2020)

Strengths and limitations of this study

- This is one of the first trials to assess the feasibility of FMT as a possible treatment for antimicrobial resistance
- The randomised, placebo-controlled design will control for spontaneous loss of carriage of resistant organisms
- The use of a capsulized FMT preparation with a single face-to-face follow up visit, places a low burden on patients to participate
- Qualitative data from participant focus groups will inform and influence a potential future trial
- This study will assess feasibility; however it is not statistically powered to assess clinically efficacy, which will need to be evaluated in a substantive trial
- Mechanistic outcomes using metagenomic, metabolomic and host immune analyses could provide insight into the mechanism of action of FMT in treatment responders
- The lack of investigator blinding, and the single centre design is a limitation

For peer review only

Introduction

Antimicrobial resistance (AMR) in enterobacteriales is increasing, fuelled by the indiscriminate use of antimicrobials and inadequate infection control practices. Of greatest concern are extended spectrum beta-lactamase producing (ESBL-E) and carbapenemase producing enterobacteriales (CPE). Rates of ESBL producing bacteria carriage in our local population are 9%, with the majority being CTX-M type (1). Rates of detection and infections caused by ESBL-E/ CRE are increasing nationally and globally (2, 3), resulting in a significant burden of attributable death and disability adjusted life years (4). Antimicrobial resistant bacteria (ARB) such as ESBL-E/ CPE have the capacity to spread between individuals and between organisms through horizontal gene transfer. These organisms have been responsible for several large and prolonged outbreaks worldwide (5-7). As well as increased morbidity and mortality, infection with resistant organisms is associated with prolonged hospital stay and increased healthcare costs (4, 8, 9). Hospitalised patients are particularly at risk of acquiring these organisms due to the treatments and procedures they receive, their comorbidities, and their high exposure to antimicrobials (5).

The microbiota of the human gut is a complex ecosystem and the largest reservoir of ARB (10, 11). A better understanding of the human microbiome has led to a new appreciation for the role indigenous microbes play in protecting us from invading exogenous pathogens. The role of the gut microbiota in defending the host against gastrointestinal pathogens was first described in a mouse model in which streptomycin administered orally to disrupt the gut microbiota resulted in increased rate of Salmonella enterica-related infections (12). Antimicrobials disrupt the balance of the delicate gut ecosystem, enabling colonisation by ESBL-E/ CPE and other potential pathogens. This is most strikingly evident in patients suffering from Clostridioides difficile infection (CDI), and the remarkable success of modulating this with faecal microbiota transplantation (FMT) (13, 14).

Attempts to control carriage of ARB in the gut using selective digestive decolonisation (SDD) are controversial, have not been widely adopted, and is not recommended by expert groups (15). Loss of ARB colonisation has been observed in a number of patients when using FMT to treat recurrent CDI (16). However, these reports are nearly all case series which are uncontrolled and do not account for spontaneous loss of carriage, which can occur in up to 50% of patients following hospital discharge (17).

The only published randomised trial of FMT to eradicate gastrointestinal carriage of ESBL-E and CPE was conducted in four academic centres in Geneva, Paris, Utrecht and Tel Aviv (18). Patients were randomised in a 1:1 ratio to a five-day course of colistin and neomycin followed by FMT or no intervention. The primary outcome measure was culture of ESBL-E / CPE from stool 35-48 days following randomisation, which was achieved for 41% (9/22) of patients in the intervention arm vs. 29% (5/17) in the control arm. Although the odds ratio for decolonisation success for FMT was 1.7 (95% confidence interval 0.4-6.4), this was not statistically significant, leaving the authors to conclude that the results do not support the routine use of FMT for decolonisation. Although the study was multicentre and included a control group to account for spontaneous loss of carriage, there are several limitations with the design and conduct, making it difficult to draw firm conclusions. Firstly, although designed as a superiority trial with a sample size calculation of 32 in each group, only 39

For peer review only

(61%) patients in total were randomised (due to recruitment problems). Secondly, patients in the intervention arm received five days of colistin and neomycin in addition to FMT, whereas the controls received no intervention. Thus, it is impossible to determine whether the results were due to the antibiotics (likely to have a profound effect on the gut flora) vs. FMT. Thirdly, the methods of administration of FMT varied according to recruiting site; capsules were administered in two centres (16 patients), whilst two used nasogastric administration (6 patients). The capsules (15 administered each day over two days) were produced from one donation derived from 15-30g faeces. The nasogastric preparation was derived from 40g. There is evidence in the context of recurrent CDI that FMT preparations made with less than 50g faeces result in poorer outcomes than those made with more than this amount (19). Thus, a question exists over whether the patients were under dosed, and if repeated administrations (perhaps using different donors) might be more effective. Lastly, the study was not placebo controlled or blinded, although the primary outcome of stool culture at one month is fairly objective, there is the possibility of introducing bias in an investigator who is aware of the allocation.

Due to the limitations of the above study, the lack of other rigorously conducted, well controlled studies, and the considerable doubt that sufficient patients would be willing to participate in research of this type, we designed a feasibility study to address some of the outstanding questions.

Methods and analysis

Primary objectives

The primary objective of this study is to determine the feasibility and acceptability of administering encapsulated FMT to participants colonised with ESBL-E / CPE. This will be used to determine if a substantive trial is feasible.

Primary endpoints

The primary outcome measure is consent rate (as a proportion of patients who fulfil inclusion/ exclusion criteria). The success criteria for the primary endpoint are stratified. If <15% is achieved, progression to a substantive trial will not be deemed feasible. If 15-39%, progression to a substantive trial will be deemed feasible with protocol modifications and clearly defined stop/go criteria. An overall consent rate of >40% will be taken as indicating a substantive trial is feasible.

Secondary objectives

The secondary objectives are to assess other feasibility aspects of conducting a substantive trial, to evaluate the safety and tolerability of FMT in this patient population, and to provide early evidence of efficacy. These measures should inform a future trial, such as determining the primary (efficacy) outcome and sample size, if progression criteria are met. A full list of criteria for progression to a substantive trial are details in Table 1 of supplementary file.

Secondary feasibility endpoints

- Proportion of patients fulfilling inclusion / exclusion criteria
- Proportion of patients receiving FMT / placebo (as a % of those consenting)
- Proportion of patients returning for follow up visits (face to face visit at Day 40)

For peer review only - http://bmjopen.bmj.com/site/about/guidelines.xhtml

• Proportion of patients providing follow up stool samples (Days 10, 40, 100 and 190)
• Ability to recruit sufficient healthy donors to manufacture all FMT doses to meet demands of this and a future substantive RCT. Assessed by delay in dosing patients (measured in days)

Additional feasibility assessments will include:
• Collection of data that may be used in estimating of costs/resources needed to provide FMT in the NHS.
• An embedded qualitative study to explore views and experiences of research participants.

Secondary efficacy endpoints
• Gastrointestinal carriage of CRE / ESBL (detected / not detected) by stool culture over time (days 10, 40, 100 and 190)
• Gastrointestinal carriage of CRE / ESBL (detected / not detected) by multiplex PCR over time (days 10, 40, 100 and 190)

Secondary safety and tolerability endpoints
• proportion of patients experiencing reflux following administration of FMT
• Proportion of patients suffering intolerable (resulting in withdrawal from the study) gastrointestinal side effects (including diarrhoea, constipation, abdominal pain, flatulence and bloating). This will be assessed by direct questioning and completion of a short patient questionnaire.
• identification of unanticipated harms involved with administration of FMT.
• occurrence of any adverse event / serious adverse event

Exploratory endpoints/outcomes
The following exploratory / mechanistic outcomes will be measured:

• Changes in the gut microbiome induced by capsulized FMT as measured by comparing between treatment groups change (relative to baseline) in;

- the proportion and relative abundance of bacterial taxa over time (days 10, 40, 100 and 190)
- the change in diversity of the microbiome over time (days 10, 40, 100 and 190) measured using Shannon and Simpson indices
- antibiotic resistance genes carriage over time (days 10, 40, 100 and 190)

• Changes in the gut metabolome induced by capsulized FMT (using Nuclear Magnetic Resonance (NMR) spectroscopy). Measured at days 10, 40, 100 and 190.

• Host immune response T and B cell) as measured by comparing participants prior to and day 40, as well as donors who will act as controls.

Trial design
Randomised control participant-blinded, single-centre, feasibility trial with two parallel groups (FMT capsules and matched placebo). Eighty patients will be randomised 1:1 (40 will receive FMT capsules and 40 placebo) from eligible patients identified from Guy's and St Thomas' hospitals (figure 1 & table 1).

For peer review only - http://bmjopen.bmj.com/site/about/guidelines.xhtml

Patient and public involvement

Patients and the public have identified antimicrobial resistance as a research priority and were involved in identifying the research question and providing feedback on the grant application. A patient representative has been appointed to the Trial Steering Committee (TSC), and has advised on the design of the research, the protocol and all patient facing materials. The patient representative will also be involved in dissemination of the study findings. As the acceptability of FMT in this setting is a key research question we will invite up to eight patients to participate in focus groups. The aim of the group will be to understand their experience in participation in the study and will focus on acceptability, barriers to participation and improvements that could be made to any resulting substantive trial.

Patient population

Participants will be recruited from Guy's and St Thomas' NHS Foundation Trust, a 1200 bed academic centre in central London. It is anticipated that most patients will already be admitted to the hospital as part of standard of care treatment, thus, most activities will take place on the ward or clinical area that the patient is already located. Where this is not the case, participants will be invited to attend the infection clinical room on an outpatient basis.

Consent

Informed consent (for both healthy donors and patient-recipients) will be obtained prior to any trial related activities, including screening for eligibility. Potential participants will be given the participant information sheet (PIS) and allowed enough time to read thoroughly and discuss with others outside of the study team (e.g. family, friends, general practitioner) (see online supplementary file table 2). Participants are free to withdraw from the trial at any time without giving reasons. Data and samples collected up to the point of withdrawal will only be used after withdrawal if the participant consented for this. Patients who lack capacity will not be enrolled in this study. Where a participant consents but later becomes incapacitated, the original consent given endures the loss of capacity, providing that the trial has not significantly altered.

Randomisation

The randomisation schedule will be generated using a validated online randomisation programme, hosted by King's Clinical Trials Unit. The method of randomisation will be block randomisation with randomly varying block sizes. As this is a single centre study, randomisation does not need to be stratified. Participants will be allocated treatment as close as possible to receiving it.

Study intervention

FMT for this trial will be prepared in a lyophilised, encapsulated form in accordance with Good Manufacturing Practice (GMP) principles and under manufacturing authorisation for an Investigational Medicinal Product (IMP) from the Medicines and Healthcare Products Regulatory Agency (MHRA). Our centre has recently provided FMT for a CTIMP for cirrhosis and this follows similar processes (20). Healthy donor inclusion and exclusion criteria and screening and eligibility questionnaire are described in Tables 3 and 4 of the supplementary file.

For peer review only - http://bmjopen.bmj.com/site/about/guidelines.xhtml

The product contains 0.9% sodium chloride and 5% trehalose (cryoprotectant) as excipients. A minimum of 80g faeces from each donor will be used to manufacture one batch of five capsules. Following lyophilisation, the material will be encapsulated in five size 0 delayed release methylcellulose capsules (DRcaps™, Capsugel®, Livingston, UK). Placebo capsules will contain microcrystalline cellulose. The capsules for the FMT and placebo will be identical in appearance. The capsules are coloured Swedish orange, resulting in an opaque appearance through which the contents cannot be seen.

FMT donors are carefully screened healthy volunteers with a body mass index between 18-30. Donors undergo questionnaire screening for risk factors and testing for a range of infectious agents as previously described and in accordance with national guidelines (see appendix A for full details) (21). FMT material is traceable from donor to recipient. Aliquots of donor stool will be kept for 30 years to allow for future testing if required.

At baseline participants will have their medication history recorded, including over the counter preparations and supplements as well as pre/probiotics. Vital signs, height and weight, and baseline blood biochemistry and haematology will be collected. Additionally, a serum sample will be stored to allow future testing in the event of a possible transmission event. If female and of child-bearing age a urinary pregnancy test will be performed. An EQ-5D questionnaire will also be administered.

Encapsulated FMT (IMP) and placebo will be dispensed by study staff to trial participants over three consecutive days (or over five days if over a weekend). Patients will be fasted for 4 hours and be pre-treated with omeprazole on the morning on the FMT (40mg on first dosing day and 20mg on the two subsequent dosing days).
It is anticipated that most patients will remain an inpatient for the duration of treatment. If they have been discharged in the interim provision will be made for them to attend for treatment as an outpatient.

Evaluations during and after treatment
Follow up events will be scheduled for all participants at 1 week, 1 month, 3 months and 6 months after end of therapy. If an outpatient, the visits at 1 week, 3 months and 6 months will be conducted by telephone, with the participant returning a stool sample by post. The follow-up at 1 month will be face to face and will include a blood sample for immune analyses. All visits will involve completion of an EQ-5D questionnaire (see figure 2 for additional details).

Sample analyses
Stool samples will be analysed for the presence of ESBL-E/ CPE using culture based (chromogenic agar with species identification using MALDI-ToF mass spectrometry and phenotypic antimicrobial susceptibility testing) and molecular techniques (multiplex PCR panel for 16 ESBL/ CPE resistance genes).

Follow-up
If a participant fails to present for follow up assessment, all attempts to contact the participant and information received during contact attempts will be documented in the participant's medical record. In any circumstance, every effort will be made to contact the

For peer review only - http://bmjopen.bmj.com/site/about/guidelines.xhtml

participant and document outcome (i.e. three documented contact attempts via phone calls, on separate occasions will be made to locate or contact the participant, and/or determine health status). Stool samples will be stored for further follow-on analysis, including metagenomics and metabolomics profiling.

Qualitative study

A qualitative study of participant's experiences will be undertaken and comprises a focus group with a minimum of eight participants. Ideally the group will include at least two patients who were approached but did not agree to participate. The aim of these discussions is to identify facilitators and barriers to delivering the trial, and whether there are any aspects of the trial that should be changed.

Objectives of the focus group will be;

- Identifying ways of increasing recruitment and retention
- Identifying ways of broadening participation in the trial to improve diversity of population
- Improving understanding of how participants join trials and experience of participation
- Measuring reasons for non-adherence to the trial medication
- Exploring stakeholders' views of acceptability of the trial design
- Strengthening the ethical conduct of the trial, for example, informed consent procedures
- Addressing any local issues which may impact on the feasibility of a substantive trial
- Understanding how the trial affects different stakeholders, for example, workload

Statistical analysis

Sample size

As this is a feasibility study, significance tests between or within groups will not be performed for the study's primary and secondary endpoints, therefore a power calculation has not been performed. For feasibility and pilot studies, sample sizes between 24 and 50 have been recommended to estimate a chosen parameter (22, 23). We have chosen a 1:1 treatment to placebo ratio, therefore a total sample size of 80 would be enough to estimate the standard deviation of the outcome in 40 treated patients, allowing for some loss to follow-up. We will also be able to estimate our expected recruitment rate of 40% (95% CI: 33-47) if we approach around 200 eligible patients.

Data synthesis, analysis and presentation

A Statistical Analysis Plan will be written by the trial statistician and signed off prior to database lock. The study will be reported in accordance with the CONSORT extension for pilot and feasibility studies.

For peer review only

The proportion of patients who accept the offer of randomisation will be reported with 95% confidence intervals computed by the exact binomial method. No statistical tests for significant differences between treatment groups will be performed. In addition to summary statistics of the secondary outcomes, all harms and withdrawals will be reported with 95% confidence intervals. Patients will be analysed in the groups to which they are randomised in accordance with intent to treat principals.

The protocol has been designed to place minimal burden upon patients and case report forms are only capturing essential data. It is inevitable that there may be some missing data, which will be reported by treatment group with reasons for missingness described, where possible. Since this is a feasibility study, we do not plan to impute missing data.

Statistical software
All statistical analysis will be conducted using Stata version 15.0 or above (StatCorp, Texas).

Trial monitoring groups

Trial Management Group (TMG)
Comprises the chief investigator (CI), trial statistician, trial staff and other lead clinical and non-clinical co-investigators and co-applicants. The TMG are responsible for the day-to-day management of the trial and to ensure all practical details of the trial are progressing and working well. The TMG will monitor all aspects of the conduct and progress of the trial, ensuring that the protocol is adhered to and take appropriate action to safeguard participants and the quality of the trial itself. The TMG will be responsible for drafting of the final report and submission for publication.

Trial Steering Committee (TSC)
A Trial Steering Committee (TSC) will be convened with membership nominated by the CI in partnership with the sponsor. The role of the TSC is to provide overall supervision for the trial on behalf of the sponsor and funder and to ensure that the project is conducted to the rigorous standards set out in the Department of Health and Social Care's Research Governance Framework for Health and Social Care and the Guidelines for Good Clinical Practice. The committee Chair will be independent of the study. The committee will also comprise four other independent members (Consultant Microbiologists or Gastroenterologists) a patient/public representative, and an independent statistician.
The TSC will take responsibility for monitoring data and making recommendations to the TMG on whether there are any ethical or safety reasons why the trial should not continue. A separate Data Monitoring and Ethics Committee (DMEC) will not be established as this is a single centre feasibility trial with a relatively small number of patients using an established IMP with a relatively well described safety profile.

Ethics and dissemination
Research ethics approval was obtained by London - City & East Research Ethics Committee (ref. 20/LO/0117). Trial results will be published in a peer-reviewed journal and presented at international conferences.

<u>Discussion</u>

For peer review only

Several case reports and one randomised controlled trial of ARB decolonisation using FMT are summarised in four systematic reviews (16, 24-26). Most studies were case reports or case series which did not control for spontaneous loss of ARB carriage. This is important since it may be significant and may lead to overestimation of the effectiveness of FMT in achieving decolonisation. In a recent study conducted at Central Manchester Foundation Trust during 2016/17 only 17.1% of patients who were previously known to be colonised with CRE had it detected on readmission to the hospital (27). Therefore, the use of a placebo in this trial is justified and crucial to control for spontaneous loss of carriage.

Capsule administration has been selected following consultation with patient groups. It is more acceptable and cost effective than other methods of administration such as via nasojejunal tube.

Although the underlying mechanism of action of FMT is not fully elucidated, the use of three different donors is justified as it likely increases the bacterial diversity in the administered IMP, with the hope that this will engraft in the recipient. The previous study using a single donor resulted in an odds ratio for decolonisation success of 1.7 (95% CI 0.4-6.4). We hypothesize that using multiple donors at three dosing points will result in a higher rate of decolonisation. This is also based on experience of using FMT to treat patients with ulcerative colitis, where multiple donors are used in prolonged treatment intervals of up to six weeks (28-31).

The overall aim of this programme of work (which would proceed to a future substantive RCT if feasible) is to eradicate or suppress ESBL-E / CRE without resorting to the use of antibiotics. If that can be achieved, then the risk of an invasive infection with ARB in these patients could be significantly reduced.

Acknowledgements
EJR receives salary support from the NIHR Biomedical Research Centre based at Guy's and St Thomas' NHS Foundation Trust and King's College, London

Collaborators
Annapurna Vyakarnam, David Moyes, James Mason

Contributors
SG and BM conveived and designed the trial, drafting of the manuscript
EJR and CB designed the statistical aspects of the study.
MSH designed the exploratory immunology aspects of the study.
GCAA and JB designed exploratory microbiome aspects of the study.
DLS is Chair of the Trial Steering Committee and provided expert advice.
CCI provided input from the patient and public perspective.
All authors reviewed and approved the final manuscript.

Sponsor
The study sponsor is Guy's and St Thomas' NHS Foundation Trust, Great Maze Pond, London SE1 9RT. The sponsor had no role in the design of this study and will not have any role during its execution, analyses, interpretation of the data, or decision to submit results.

Funding

For peer review only

This independent research is supported by the National Institute for Health Research (NIHR) under its Research for Patient Benefit (RfPB) Programme (grant reference number PB-PG-0418-20007). The funder had no role in the design of this study and will not have any role during its execution, analyses, interpretation of the data, or decision to submit results.

Competing interests

SDG has received personal fees from Astellas, Enterobiotix, Menarini, MSD, Pfizer and Shionogi.
DLS has undertaken paid consultancy for Norgine Ltd, Shionogi and Kaleido Biosciences and paid lectures for Falk Pharma, Norgine Ltd and Alfa Sigma
All other authors report no competing interests.

Patient consent for publication

Not required.

Ethics approval

Research ethics approval was given by London - City & East Research Ethics Committee (ref. 20/LO/0117).

For peer review only

References

1. J. A. Otter, A. Natale, R. Batra, O. Tosas Auguet, E. Dyakova, S. D. Goldenberg, et al. Individual- and community-level risk factors for ESBL Enterobacteriaceae colonization identified by universal admission screening in London. Clin Microbiol Infect. 2019;25(10):1259-65.
2. Public Health England. English Surveillance Programme for Antimicrobial Utilisation and Resistance (ESPAUR) Report. 2019.
3. V. Gupta, G. Ye, M. Olesky, K. Lawrence, J. Murray, K. Yu. Trends in resistant Enterobacteriaceae and Acinetobacter species in hospitalized patients in the United States: 2013-2017. BMC Infect Dis. 2019;19(1):742.
4. A. Cassini, L. D. Hogberg, D. Plachouras, A. Quattrocchi, A. Hoxha, G. S. Simonsen, et al. Attributable deaths and disability-adjusted life-years caused by infections with antibiotic-resistant bacteria in the EU and the European Economic Area in 2015: a population-level modelling analysis. Lancet Infect Dis. 2019;19(1):56-66.
5. K. van Loon, A. F. Voor In 't Holt, M. C. Vos. A Systematic Review and Meta-analyses of the Clinical Epidemiology of Carbapenem-Resistant Enterobacteriaceae. Antimicrob Agents Chemother. 2018;62(1).
6. L. Tavoschi, S. Forni, A. Porretta, L. Righi, F. Pieralli, F. Menichetti, et al. Prolonged outbreak of New Delhi metallo-beta-lactamase-producing carbapenem-resistant Enterobacterales (NDM-CRE), Tuscany, Italy, 2018 to 2019. Euro Surveill. 2020;25(6).
7. V. Decraene, H. T. T. Phan, R. George, D. H. Wyllie, O. Akinremi, Z. Aiken, et al. A Large, Refractory Nosocomial Outbreak of Klebsiella pneumoniae Carbapenemase-Producing Escherichia coli Demonstrates Carbapenemase Gene Outbreaks Involving Sink Sites Require Novel Approaches to Infection Control. Antimicrob Agents Chemother. 2018;62(12).
8. H. Suzuki, E. N. Perencevich, D. J. Livorsi, B. Alexander, B. F. Beck, K. K. Richardson, et al. Attributable mortality due to fluoroquinolone and extended-spectrum cephalosporin resistance in hospital-onset Escherichia coli and Klebsiella spp bacteremia: A matched cohort study in 129 Veterans Health Administration medical centers. Infect Control Hosp Epidemiol. 2019;40(8):928-31.
9. C. E. French, C. Coope, L. Conway, J. P. Higgins, J. McCulloch, G. Okoli, et al. Control of carbapenemase-producing Enterobacteriaceae outbreaks in acute settings: an evidence review. J Hosp Infect. 2017;95(1):3-45.
10. C. J. Donskey. The role of the intestinal tract as a reservoir and source for transmission of nosocomial pathogens. Clin Infect Dis. 2004;39(2):219-26.
11. J. Carlet. The gut is the epicentre of antibiotic resistance. Antimicrob Resist Infect Control. 2012;1(1):39.
12. M. Bohnhoff, C. P. Miller, W. R. Martin. Resistance of the Mouse's Intestinal Tract to Experimental Salmonella Infection. Factors Which Interfere with the Initiation of Infection by Oral Inoculation. J Exp Med. 1964;120:805-16.
13. E. van Nood, M. G. Dijkgraaf, J. J. Keller. Duodenal infusion of feces for recurrent Clostridium difficile. N Engl J Med. 2013;368(22):2145.
14. P. Moayyedi, Y. Yuan, H. Baharith, A. C. Ford. Faecal microbiota transplantation for Clostridium difficile-associated diarrhoea: a systematic review of randomised controlled trials. Med J Aust. 2017;207(4):166-72.

15. E. Tacconelli, F. Mazzaferri, A. M. de Smet, D. Bragantini, P. Eggimann, B. D. Huttner, et al. ESCMID-EUCIC clinical guidelines on decolonization of multidrug-resistant Gram-negative bacteria carriers. Clin Microbiol Infect. 2019;25(7):807-17.
16. S. Saha, R. Tariq, P. K. Tosh, D. S. Pardi, S. Khanna. Faecal microbiota transplantation for eradicating carriage of multidrug-resistant organisms: a systematic review. Clin Microbiol Infect. 2019;25(8):958-63.
17. J. P. Vink, J. A. Otter, J. D. Edgeworth. Carbapenemase-producing Enterobacteriaceae - once positive always positive? Curr Opin Gastroenterol. 2020;36(1):9-16.
18. B. D. Huttner, V. de Lastours, M. Wassenberg, N. Maharshak, A. Mauris, T. Galperine, et al. A 5-day course of oral antibiotics followed by faecal transplantation to eradicate carriage of multidrug-resistant Enterobacteriaceae: a randomized clinical trial. Clin Microbiol Infect. 2019;25(7):830-8.
19. E. Gough, H. Shaikh, A. R. Manges. Systematic review of intestinal microbiota transplantation (fecal bacteriotherapy) for recurrent Clostridium difficile infection. Clin Infect Dis. 2011;53(10):994-1002.
20. C. A. Woodhouse, V. C. Patel, S. Goldenberg, A. Sanchez-Fueyo, L. China, A. O'Brien, et al. PROFIT, a PROspective, randomised placebo controlled feasibility trial of Faecal mIcrobiota Transplantation in cirrhosis: study protocol for a single-blinded trial. BMJ Open. 2019;9(2):e023518.
21. S. D. Goldenberg, R. Batra, I. Beales, J. L. Digby-Bell, P. M. Irving, L. Kellingray, et al. Comparison of Different Strategies for Providing Fecal Microbiota Transplantation to Treat Patients with Recurrent Clostridium difficile Infection in Two English Hospitals: A Review. Infect Dis Ther. 2018;7(1):71-86.
22. S. A. Julious. Two-sided confidence intervals for the single proportion: comparison of seven methods by Robert G. Newcombe, Statistics in Medicine 1998; 17:857-872. Stat Med. 2005;24(21):3383-4.
23. J. Sim, M. Lewis. The size of a pilot study for a clinical trial should be calculated in relation to considerations of precision and efficiency. J Clin Epidemiol. 2012;65(3):301-8.
24. M. H. Woodworth, M. K. Hayden, V. B. Young, J. H. Kwon. The Role of Fecal Microbiota Transplantation in Reducing Intestinal Colonization With Antibiotic-Resistant Organisms: The Current Landscape and Future Directions. Open Forum Infect Dis. 2019;6(7).
25. V. Tavoukjian. Faecal microbiota transplantation for the decolonization of antibiotic-resistant bacteria in the gut: a systematic review and meta-analysis. J Hosp Infect. 2019;102(2):174-88.
26. B. Davido, R. Batista, A. Dinh, P. de Truchis, E. M. Terveer, B. Roberts, et al. Fifty shades of graft: How to improve the efficacy of faecal microbiota transplantation for decolonization of antibiotic-resistant bacteria. Int J Antimicrob Agents. 2019;53(5):553-6.
27. A. Tucker, R. George, W. Welfare, P. Cleary, J. Cawthorne, A. Dodgson. Screening for carbapenemase-producing Enterobacteriaceae in previous carriers readmitted to hospital: evaluation of a change in screening policy. J Hosp Infect. 2019;103(2):156-9.
28. P. Moayyedi, M. G. Surette, P. T. Kim, J. Libertucci, M. Wolfe, C. Onischi, et al. Fecal Microbiota Transplantation Induces Remission in Patients With Active Ulcerative Colitis in a Randomized Controlled Trial. Gastroenterology. 2015;149(1):102-9 e6.
29. N. G. Rossen, S. Fuentes, M. J. van der Spek, J. G. Tijssen, J. H. Hartman, A. Duflou, et al. Findings From a Randomized Controlled Trial of Fecal Transplantation for Patients With Ulcerative Colitis. Gastroenterology. 2015;149(1):110-8 e4.

For peer review only - http://bmjopen.bmj.com/site/about/guidelines.xhtml

30. S. Paramsothy, M. A. Kamm, N. O. Kaakoush, A. J. Walsh, J. van den Bogaerde, D. Samuel, et al. Multidonor intensive faecal microbiota transplantation for active ulcerative colitis: a randomised placebo-controlled trial. Lancet. 2017;389(10075):1218-28.
31. S. P. Costello, O. Waters, R. V. Bryant, R. Katsikeros, J. Makanyanga, M. Schoeman, et al. Short Duration, Low Intensity, Pooled Fecal Microbiota Transplantation Induces Remission in Patients with Mild-Moderately Active Ulcerative Colitis: A Randomised Controlled Trial. Gastroenterology. 2017;152(5):S198-S9.

For peer review only

Figure 1: CONSORT flow diagram

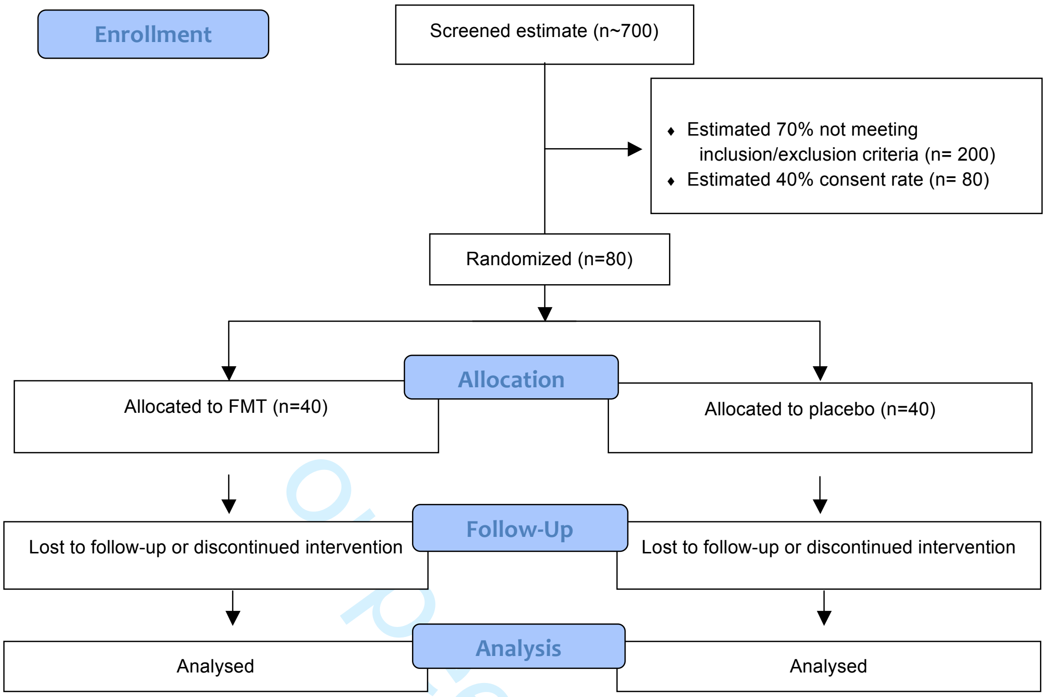

For peer review only

Figure 2: Intervention and follow-up

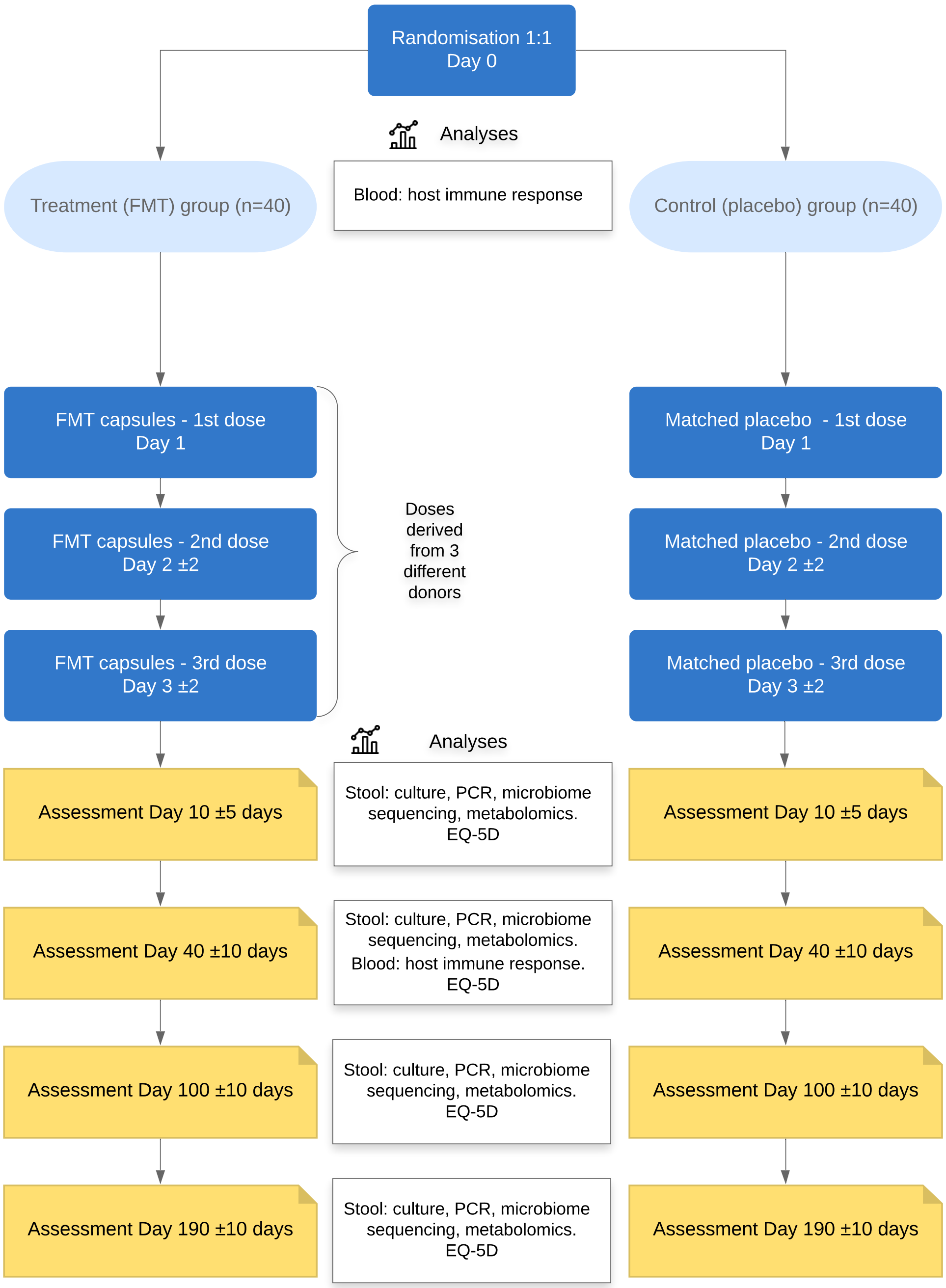

Table 1: Participant inclusion/ exclusion criteria

Inclusion criteria

To be eligible for enrolment, a participant must meet all the following criteria before undergoing any study-related procedures:

- Adult patients (age 18 years or older at time of consent) **AND**
- Current/ previous patient at Guy's and St Thomas' NHS Foundation Trust **AND**
- Ability to understand the purpose, potential benefits and risks of the study and capable of giving informed consent. The participant must be able to provide written informed consent **AND**
- Documented gastrointestinal carriage of ESBL-E or CPE (stool sample) in the 21 days prior to consent **AND**
- Symptomatic infection with the same target organism of interest in preceding 6 months (this needs to be microbiologically confirmed but is not restricted to any particular body site e.g. could be urinary tract infection, intra-abdominal infection, blood stream infection etc).

Exclusion criteria

- Pregnancy or planned pregnancy
- Breastfeeding
- Severe or life-threatening food allergy
- Allergy or other contraindication to omeprazole, IMP or placebo ingredients
- Treatment with systemic antibiotic on the day of and day prior to 1st IMP/placebo dosing to the end of the dosing period
- Treatment with pre or probiotics in the 4 weeks prior to randomisation and for the duration of the study
- Severe immunodeficiency:
  - systemic chemotherapy <30 days from baseline or planned chemotherapy within the upcoming 6 months
  - Known HIV infection with CD4 count <250 cells/uL
  - Known neutropenia with absolute neutrophils $<1.0x10^9$
  - Prolonged treatment with corticosteroids (equivalent to prednisone >60mg daily for > 30 days) within 8 weeks of randomisation
- Life expectancy <6 months
- Swallowing disorder, oral-motor dyscoordination or likely inability/unwillingness to ingest study medication
- Patients who have received another investigational drug or device within 4 months prior to randomisation
- Any condition or circumstance, in the opinion of the investigator, that would compromise the safety of the patient or the quality of the study data

For peer review only

Supplementary file S1.

FERARO: A prospective, randomised placebo-controlled feasibility trial of **F**aecal microbiota Transplant to **ER**adicate gastrointestinal carriage of **A**ntibiotic **R**esistant **O**rganisms: study protocol for single-blinded trial

Table 1. Criteria for progression to a substantive trial

Criteria	Stop – substantive trial not feasible	Continue with protocol modifications, close monitoring and clearly defined stop/go points	Continue without modification – feasible as is
**Hard Feasibility Criteria<br>Used to determine progression to a substantive trial**			
Consent rate (% of patients who fulfil eligibility criteria)	<15%	15-39%	>40%
**Soft Feasibility Criteria<br>Taken into consideration in determining progression to a substantive trial**			
Proportion of patients fulfilling eligibility criteria	<10%	10-29%	>30%
% of patients receiving IMP/placebo (as % of those consenting) and compliant will all doses on all three days	<50%	50-75%	>75%
% of patients returning for follow up visit (Day 40)	<50%	50-79%	>80%
% of patients providing follow up stool samples (Days 10, 40, 100 and 190)	<50% returning two or more samples	50-79% patients returning two or more samples	>80% patients returning two or more samples
Ability to recruit sufficient healthy donors to manufacture all FMT	Delay in dosing patients >2 weeks	Delay in dosing patients up to 2 week2	No delay in patient dosing
**Soft Patient Tolerability Criteria<br>Taken into consideration in determining progression to a substantive trial**			
% of patients experiencing reflux	>51%	21-50%	<20 %

For peer review only - http://bmjopen.bmj.com/site/about/guidelines.xhtml

following FMT administration			
Intolerable (resulting in withdrawal) side effects	>51%	21-50%	<20 %

For peer review only

Table 2. Participant Information Sheet (PIS)

A prospective, randomised placebo controlled feasibility trial of Faecal microbiota Transplant to ERadicate gastrointestinal carriage of Antibiotic Resistant Organisms

The FERARO Trial

Patient Information Sheet

We would like to invite you to take part in the FERARO trial.

- Before you decide whether you would like to take part in the trial, it's important for you to understand why the research is being done and what it would involve.
- Please take some time to read the information carefully, and discuss with your family, friends and doctor, as you wish.
- Ask us if anything is not clear, or you would like some more information

Important things that you need to know:

- Taking part is completely up to you and you can stop taking part at any time, without giving a reason. If you do not wish to take part, this will not affect the care you receive from your doctors or other health care professionals.
- You have been asked to take part in this study because a sample you have provided contains Antibiotic Resistant Bacteria (ARB). This study is testing the acceptability of a treatment that may be able to reduce the amount of ARB in the gut and the risk of infection.
- Faecal Microbiota Transplant (FMT) is a capsule made up of bacteria taken from a stool (poo) sample donated by healthy people. It could help to restore the balance of bacteria in the gut by reducing the amount of bacteria that are resistant to antibiotics.
- Faecal Microbiota Transplant (FMT) is a treatment used currently to treat patients with repeated Clostridioides difficile (C. diff) infection.
- The FERARO trial is looking to see if you consider this to be an acceptable treatment and if there are any side effects.

For peer review only

- You will be allocated to receive either FMT capsules or a placebo (dummy) capsules. You will not know which treatment you receive.
- You will be asked to take five capsules a day for three days in a row. This may be while you are already staying in hospital or as an outpatient.
- You will be asked to provide stool samples before and after receiving treatment to see if there are any changes in the bacteria present in your gut.
- You will be followed up by the study team for six months after completing the treatment.

If you have any questions about this study, please contact:

Dr Simon Goldenberg
Telephone (with message facility): 020 7188 8515
Email: simon.goldenberg@gstt.nhs.uk

What is the purpose of the study?

The human gut has trillions of good bacteria (germs or bugs) which are important to keep us healthy. In total these bugs are called the microbiota. The bugs are always evolving to beat antibiotics used to fight them and this is known as resistance. Resistance to antibiotics allows bugs to survive and spread.

Antibiotic resistant bacteria (ARB) usually live in the gut (or in the surrounding environment), where they do no harm. This is called colonisation. However, the ARB can appear and cause infection in other parts of the body that normally lack any bacteria, for example in the bladder or blood. When this happens, treatment with a more powerful type of antibiotic is usually needed. This is more likely to happen in people who are more prone to infection, including people with an underlying disease or injury, or people who are already admitted to hospital.

Antibiotic resistance is a growing and serious threat to worldwide health, and means that doctors may be limited in the types of treatments that they can offer to patients. Without effective antibiotics even simple infections could become deadly, making routine medical procedures too dangerous to perform. There is an urgent need to find new antibiotics, but this takes time and is very expensive.

Two particular groups of antibiotic resistant bacteria are known as CRE and ESBL (Carbapenem Resistant Enterobacteriales and Extended Spectrum Beta-lactamase producing bacteria).

Carbapenems and beta-lactams are some of the most powerful types of antibiotics. Some strains of bacteria make enzymes (chemicals), which allow them to destroy carbapenem and beta-lactam antibiotics which makes the bacteria resistant to the antibiotics.

For peer review only - http://bmjopen.bmj.com/site/about/guidelines.xhtml

There is growing interest in non-antibiotic treatments like Faecal Microbiota Transplant (FMT) to deal with this problem.

FMT is the transfer of bacteria from the guts of healthy donors (taken from their poo / stools) into the gut of a patient. The aim is to restore a healthy balance of bacteria (reducing harmful ones and increasing good ones). It is currently used to treat patients with repeated Clostridioides difficile infection. This is an infection causing severe diarrhoea and stomach pain, normally after having antibiotics which have harmed the microbiota.

FMT is very effective and safe in treating this group of patients, with success rates of over 80%. Initial research shows that it may be helpful in other conditions.

Why have I been invited to take part?

You have been identified by a member of your healthcare team as a carrier of ARB. You have had an infection caused by ARB in the last 6 months. This might be a urine, bloodstream, chest or other type of infection and may have been on a previous visit to the hospital or from a sample that your General Practitioner sent to our lab.

Do I have to take part?

No, participation in the study is entirely voluntary. It is up to you whether or not you wish to take part. You will be given time to read the patient information sheet and ask any questions you wish about the study.

If you decide to take part you will be given this information sheet to keep and asked to sign a consent form. If you decide to take part you are still free to withdraw at any time and without giving a reason.

What will happen if I take part?

You will be asked to provide a stool sample to first check for the presence of the ESBL or CRE bacteria. If we don't find the bacteria you will not be able to continue in the study and any samples that you have already provided will be destroyed.

If ESBL or CRE bacteria are detected, you will be asked to provide a blood sample and female participants may be asked to provide a urine sample for a pregnancy test. Once we can confirm that it is safe for you to enter the study, you will be placed in one of two groups. This will be decided by chance. One group will receive FMT treatment, and the other will receive placebo (dummy) capsules. Both groups will be given five identical capsules to take each day for three days in a row. You will not know which group you are in until the end of the study.

Before taking the trial treatment, you will be asked to fast for 4 hours. You cannot eat any food during this time but you can drink water. You will also be asked to take a commonly prescribed medication called omeprazole. This works to reduce the amount of acid in the stomach. It will help protect the good bacteria in the capsules from being damaged.

For peer review only - http://bmjopen.bmj.com/site/about/guidelines.xhtml

You will then take all 5 capsules with water or squash. A member of the study team will stay with you for a short time while you take the capsules and check for any side effects. You can eat and drink as normal shortly after taking the capsules. The capsules will only be given on weekdays. If you are already staying in hospital, you will receive the treatment during your stay. If you are allowed to go home during this time, we will ask you to return to the hospital to complete the treatment. If you are not currently admitted to hospital, you will be asked to visit the hospital as an outpatient to complete the treatment.

You will be contacted by the study team by telephone after completing the treatment. This will happen 1 week, 3 months and 6 months after receiving treatment. You will be asked to provide a stool sample using the collection kit provided, and send it back to us in the post (postage will be pre-paid). You will be asked to complete a quality of life questionnaire over the phone. We will ask you how you have been feeling and if you are taking any new medications.

We also ask that you return to St Thomas' Hospital for a visit about 1 month after taking the capsules and we will ask you to bring a stool sample at that visit. We are able to reimburse you for the travel costs of visits to the hospital needed for the study, up to a maximum of £20 per visit.

In total you will be asked to provide five stool samples.

	Assessment	Treatment Day 1	Treatment Day 2	Treatment Day 3	Follow Up 1 week, 3 months, 6 months	Follow Up 1 month
	During Hospital Stay or Visit 1	During Hospital Stay or Visit 2	During Hospital Stay or Visit 3	During Hospital Stay or Visit 4	Telephone Call	Hospital Visit
Consent form	X					
Blood sample	X					X
Stool sample	X				X	X
Pregnancy test (females)	X					
Eligibility Check	X					
Basic health check	X	X	X	X		
Quality of Life Questionnaire	X				X	X
Fasting for 4 hours		X	X	X		
Capsule administration		X	X	X		
Side effects and medication check		X	X	X	X	X

For peer review only - http://bmjopen.bmj.com/site/about/guidelines.xhtml

With your consent we will send a letter to your GP to let them know that you are taking part in the trial.

What will happen to any samples I provide?

Stool samples will be processed in the FMT laboratory at St Thomas's Hospital and divided up into smaller samples. Some of the sample will be analysed at St Thomas' Hospital and some will be sent to KCL for analysis. They will be labelled with a study ID number that links the samples to who you are. This link will be kept securely by the study team and will not be shared with anybody else. No directly identifiable information will be used to label your samples.

You will be asked to provide 14 ml of blood (equivalent to 3 teaspoons) before entering the study. This is to check that it is safe for you to enter the study. If any of these tests have already been done as part of your normal care in the previous 5 days, we will not need to repeat them.

Will my samples be used in future research?

We will ask for your consent to take a blood sample and to use the stool samples that you have already provided for use in future research in addition to the samples mentioned above. The consent for storage of samples for future research is optional and will not affect your participation in the study in any way. If you do consent to this, we will ask you for 40 ml (8 teaspoons) before receiving the study treatment. This will be taken at the same time as the initial blood sample, so doesn't require an extra needle. We will also ask for a further 40 ml (8 teaspoons) at the face to face visit about 1 month after taking the capsules.

What are the possible benefits and disadvantages of taking part?

This study is designed to look at the safety and tolerability of FMT treatment to see if a larger study would show an improvement in wellbeing for patients with ARB infections. There are few treatments available to patients with antimicrobial resistant infections.

We hope that the knowledge we would gain from this study will improve our understanding of the way in which FMT works, and the role of the treatment in antimicrobial resistance. FMT appears to be safe in the considerable numbers of patients who have received it for other reasons.

If you are in the group that receives FMT, it is possible that it will help to reduce the ARB in found in your gut and reduce the risk of further infections. However, we will not know this until we have completed this study and future studies. You may not directly benefit from taking part in this study, but the information gained may help to improve the treatment of people with your condition in the future.

If you decide to take part in the study, you may be asked to attend the hospital more frequently than you would if you choose not to take part. You will also receive more regular input from a study doctor and nurse to monitor how you are feeling after the treatment.

What is the drug that is being tested?

For peer review only - http://bmjopen.bmj.com/site/about/guidelines.xhtml

FMT is the transfer of bacteria from the guts of healthy donors (taken from their poo) into the gut of a patient. FMT is produced by carefully selecting healthy individuals who have undergone an extensive health assessment and have been tested for a wide range of infections and other diseases. We follow National guidelines when selecting volunteer donors. The donor poo sample is processed in the laboratory to concentrate the healthy bacteria and remove most of the water (freeze drying) which leaves a small amount of powder. The powder is then packed into capsules which need to be swallowed.

FMT is currently used to treat patients with repeated Clostridioides difficile infection. In these patients, FMT is administered via a tube into the stomach or the lower bowel.

FMT is very effective and safe in treating this group of patients, with success rates of over 80%.

Some patients have experienced side effects, including belching, abdominal cramps and abdominal pain. Diarrhoea and constipation has also been reported.

We hope that producing FMT in capsules will help to minimise any side effects. We will monitor you closely while you are taking FMT and ask how you are feeling after receiving the treatment.

We would like you to report any unexpected symptoms or health events to the study team, even if you think it is not related to the treatment.

Omeprazole

Omeprazole reduces the amount of acid your stomach makes. It's a widely used treatment for indigestion, heartburn and acid reflux.

Most people who take omeprazole don't have any side effects, particularly if it is taken over a short period of time, as is the case here. If you do get a side effect, it's usually mild (such as abdominal pain, constipation, diarrhoea, dizziness and dry mouth) and will go away when you stop taking omeprazole.

How is FMT made?

After the healthy volunteers have been carefully screened and tested, the stool sample is brought to the FMT laboratory. Sterile salt water will be added to the stool and it will be filtered to remove any solid material and then be freeze-dried to remove water.

The resultant material is placed into capsules and stored frozen until it is required by the person who will receive it. Once a patient has been identified and consented to take part in the FERARO study, the capsules will be administered to the patient.

We will store a small amount of the stool sample to assess the types of bacteria present (microbiota analysis) so that we can compare to the stool samples provided by the patients that take the FMT.

For peer review only

These analyses will be performed at King's College, London for the blood samples. As part of a separate study the stool sample analysis will be performed at the National Institute for Biological Standards and Controls.

All samples will be completely anonymised. Patients who receive the FMT will not be given any information about who donated the samples.

Stool will be archived for thirty years for traceability, so that in the unlikely event of an infection occurring in the recipient the donor stool can be checked for infection also. The data will be anonymised and will only be accessible to members of the trials team. After this period it will be destroyed.

Who is organising and funding this study?

The doctor in charge of this study is Dr Simon Goldenberg. The study is funded by the National Institute of Health Research and is being sponsored by Guy's and St Thomas' NHS Foundation Trust.

Who has reviewed the study?

All research in the NHS is looked at by an independent group of people, called a Research Ethics Committee, to protect your interests. This study has been reviewed and approved by the London – City and East Research Ethics committee (reference 20/LO/0117.) It has also been reviewed by an independent review group and approved by the Health Research authority and the Medicines and Healthcare products Regulatory Agency.

What if something goes wrong?

If you have a concern about any aspect of this study, you should ask to speak to your study doctor who will do their best to answer your questions (contact details on page 2 of this information sheet).

If you remain unhappy and wish to complain formally, you can do this through the NHS Complaints procedure by contacting the Patient Advice Liaison Service (PALS) office.

Guy's and St Thomas' NHS Foundation Trust PALS
Contact number: 0207 188 8801 or pals@gstt.nhs.uk

Every care will be taken in the course of this study. However in the unlikely event that you are injured by taking part, compensation may be available.

In the event that something does go wrong and you are harmed during the research and this is due to someone's negligence then you may have grounds for a legal action for compensation against Guy's and St. Thomas' NHS Foundation Trust, but you may have to pay your legal costs.

For peer review only - http://bmjopen.bmj.com/site/about/guidelines.xhtml

How will we use information about you?

We will need to use information from you and from your medical records for this research project. This information will include your hospital number, name, date of birth, address and contact details. People will use this information to do the research or to check your records to make sure that the research is being done properly.

People who do not need to know who you are will not be able to see your name or contact details. Your data will have a code number instead. We will keep all information about you safe and secure.

Once we have finished the study, we will keep some of the data so we can check the results. We will write our reports in a way that no-one can work out that you took part in the study.

What are your choices about how your information is used?

You can stop being part of the study at any time, without giving a reason, but we will keep information about you that we already have.

If you agree to take part in this study, you will have the option to take part in future research using your data saved from this study.

Where can you find out more about how your information is used?

You can find out more about how we use your information at:
www.guysandstthomas.nhs.uk/research/patients/use-of-data.aspx
at www.hra.nhs.uk/information-about-patients/
by asking one of the research team or by sending an email to the Chief Investigator
simon.goldenberg@gstt.nhs.uk

What will happen to the results of the research study?

Once the data has been analysed we plan to publish the results in an international journal so that the information can benefit as many people as possible. We can provide you with a brief summary of the results of the study when available should you desire this. If you would like to be kept informed about the results of the study, please email the Chief Investigator using the details below.

Contact details:

Thank you for taking the time to read this information. If there is any other information you would like, please do not hesitate to contact the study team on the numbers below.

Study co-ordinator:	Dr Blair Merrick	Tel: 0207 188 7188 extension 53339
Research Nurse:	Karen Bisnauthsing	Tel: 0207 188 7188 extension 53339
Chief Investigator:	Dr Simon Goldenberg	Tel: 0207 188 8515

For peer review only - http://bmjopen.bmj.com/site/about/guidelines.xhtml

Table 3. Healthy donor inclusion and exclusion criteria

**Inclusion criteria**<br>• 18-60 years of age **AND**<br>• BMI 18-30
**Exclusion criteria**<br>• Received antimicrobials within past 3 months<br>• Known prior exposure to HIV and/or viral hepatitis<br>• Known previous or latent tuberculosis<br>• Risk factors for blood borne viruses within the previous 6 months<br>• Received live attenuated vaccine within past 6 months<br>• Underlying gastrointestinal condition/ or unexplained symptoms including acute diarrhoea in 2 weeks prior to donating<br>• Family history of any significant gastrointestinal condition<br>• History of atopy, systemic autoimmune condition, diabetes, neurological or psychiatric condition or known risk of prion disease. History of chronic pain syndrome including chronic fatigue and fibromyalgia, or malignancy.<br>• Taking any regular medications<br>• History of taking proton pump inhibitors or immunosuppressive medications within the past 3 months<br>• Ever received growth hormone, insulin from cows or clotting factor concentrates<br>• Received an experimental medicine or vaccine within the past 6 months<br>• Travelled to a tropical country within the past 6 months

For peer review only

Guy's and St Thomas' NHS
NHS Foundation Trust

Table 4. Donor screening and eligibility questionnaire

Faecal Microbiota Transplant (FMT) –Donor programme

Donor name	
Donor date of birth	
Donor Hospital number	
Donor number	
Donor contact details – telephone / email	
Name of assessor	
Position	
Date of assessment	

Age	Exclude if <18 or >60
Gender	☐ Male ☐ Female
Ethnicity	☐ White - White British<br>☐ White - White Irish<br>☐ White - Other<br>☐ Mixed race – White and Black Caribbean<br>☐ Mixed race – White and Black African<br>☐ Mixed race – White and Asian<br>☐ Mixed race – Other<br>☐ Asian or Asian British – Indian<br>☐ Asian or Asian British – Bangladeshi<br>☐ Asian or Asian British – Pakistani<br>☐ Asian or Asian British – Other<br>☐ Black or Black British – Caribbean<br>☐ Black or Black British – African<br>☐ Black or Black British – Other<br>☐ Chinese<br>☐ Other
Height	cm
Weight	

For peer review only

	kg
BMI	Exclude if BMI ≤18 or ≥30
Has your weight changed by more than 5lb / 2kg in the past 6 months?	☐ Yes ☐ No<br><br>Detail:
Describe your diet (as many as apply):	☐ Omnivore<br>☐ Vegetarian<br>☐ Vegan<br>☐ Kosher<br>☐ Halal<br>☐ Raw food only<br>☐ Pescatarian<br>☐ No red meat<br>☐ Low carbohydrate<br>☐ Lactose free<br>☐ Gluten free<br>☐ Other
How many portions of fruit and vegetables do you consume per day?	☐ one or less<br>☐ two to three<br>☐ three to four<br>☐ five to six<br>☐ seven or more
How many servings of cow, sheep or goat's milk do you consume per day?	☐ one or less<br>☐ two to three<br>☐ three to four<br>☐ five to six<br>☐ seven or more
Alcohol – units/week	
Smoking/day	
Do you take any illicit drugs?	☐ Yes ☐ No<br><br>Exclude if YES
Normal bowel habit – average Bristol Stool Consistency	☐ 1<br>☐ 2<br>☐ 3<br>☐ 4<br>☐ 5<br>☐ 6<br>☐ 7<br><br>Exclude if 1, 6 or 7

For peer review only - http://bmjopen.bmj.com/site/about/guidelines.xhtml

Normal bowel habit – average frequency	☐ >2/day<br>☐ once to twice daily<br>☐ once / 2 days<br>☐ <once / 2 days<br><br>Exclude if active diarrhoea (>3 UBM/day for at least 2 consecutive days)
Have you ever been rejected as a blood donor/told not to donate?<br>If yes, why?	☐ Yes ☐ No<br><br>Exclude if YES
What is your country of birth?	
Have you ever resided in another country (other than UK) for >5 years?<br>If so which countries and when?	☐ Yes ☐ No
Do you currently have a profession that is associated with an increased risk of blood-borne transmissible diseases (e.g. daily contact with patients/inmates)?<br>If yes, what profession?	☐ Yes ☐ No<br><br>Exclude if health/social care worker with direct patient contact
Have you ever had a needle-stick or injury from a blood contaminated object from someone else?<br><br>If yes, when and how was this follow up?	☐ Yes ☐ No
Have you ever injected yourself or been injected with illegal or non-prescribed drugs including body building drugs or cosmetics (even if this was only once or a long time ago?)	☐ Yes ☐ No<br><br>Exclude if YES
Have you ever had a tattoo?<br>If yes, when and in which country was it performed?	☐ Yes ☐ No<br><br>Exclude if within past 6 months
Have you ever had a piercing?<br>If yes, when and in which country was it performed?	☐ Yes ☐ No<br><br>Exclude if within past 6 months
Have you ever had acupuncture?<br>If yes, when and in which country was it performed?	☐ Yes ☐ No<br><br>Exclude if within past 4 months
Have you ever had an operation or undergone clinical treatment in	☐ Yes ☐ No

For peer review only - http://bmjopen.bmj.com/site/about/guidelines.xhtml

a hospital with poor hygienic conditions?<br>If yes, when and in which country was it performed?	
Have you ever had a rare infectious disease (e.g. tuberculosis, malaria, trypanosomiasis)?<br>If yes, when and which disease?	☐ Yes ☐ No
Have you ever been vaccinated against Hepatitis A or B?<br>If yes, which?	☐ Yes ☐ No
In the last 12 months have you had sex with anyone who is HIV positive?	☐ Yes ☐ No<br>Exclude if Yes
In the last 12 months have you had sex with anyone with hepatitis B, hepatitis C or HTLV?	☐ Yes ☐ No<br>Exclude if Yes
In the last 12 months have you had sex with anyone who has ever been given money or drugs for sex?	☐ Yes ☐ No<br>Exclude if Yes
In the last 12 months have you had sex with anyone who has ever injected drugs?	☐ Yes ☐ No<br>Exclude if Yes
In the last 12 months have you had sex with anyone who may ever have had sex in parts of the world where HIV/AIDS is very common (this includes most countries in Africa)?	☐ Yes ☐ No<br>Exclude if Yes
**Male donors ONLY:**<br>In the last 12 months have you ever had oral or anal sex with a man, with or without a condom?	☐ Yes ☐ No<br>Exclude if Yes
**Female donors ONLY:**<br>In the last 12 months have you had sex with a man who has ever had oral or anal sex with another man, with or without a condom?	☐ Yes ☐ No<br>Exclude if Yes
Have you ever been treated for an intestinal infection?<br>If yes, which one and when?	☐ Yes ☐ No
Do you have any gastro-intestinal conditions:<br>Barretts Oesophagus<br>Coeliac disease<br>Diverticular disease	 <br>☐ Yes ☐ No<br>☐ Yes ☐ No<br>☐ Yes ☐ No<br>☐ Yes ☐ No

For peer review only

Bariatric surgery<br>Gastric ulcer<br>Gasto-oesophageal reflux disease<br>Hepatitis<br>H. pylori infection<br>Crohns disease<br>Ulcerative colitis<br>Other inflammatory bowel diseases<br>Irritable Bowel Syndrome<br>Lactose intolerance<br>Liver disease<br>Pancreatitis<br>Gastrointestinal malignancy or polyps	☐ Yes ☐ No<br>☐ Yes ☐ No<br>☐ Yes ☐ No<br>☐ Yes ☐ No<br>☐ Yes ☐ No<br>☐ Yes ☐ No<br>☐ Yes ☐ No<br>☐ Yes ☐ No<br>☐ Yes ☐ No<br>☐ Yes ☐ No<br>☐ Yes ☐ No<br>☐ Yes ☐ No<br><br>Exclude if Inflammatory Bowel Disease, Irritable Bowel Syndrome, GI malignancy, Hepatitis
Is there any family history of inflammatory Bowel Disease or colorectal cancer?	☐ Yes ☐ No<br><br>Exclude if Yes
Have you taken any antibiotics in the last 3 months?	☐ Yes ☐ No<br><br>Exclude if Yes
Have you had a fever in the last 2 weeks?	☐ Yes ☐ No<br><br>Exclude if Yes
Have you received a live vaccination within the past 6 months?	☐ Yes ☐ No<br><br>Exclude if Yes
Have you ever been incarcerated in prison?	☐ Yes ☐ No<br><br>Exclude if in past 4 months
Have you ever been immunosuppressed (e.g. during treatment for cancer, or for a solid organ transplant)?<br>If yes, when and why?	☐ Yes ☐ No
Have you ever had major gastrointestinal surgery?<br>If yes, when and why?	☐ Yes ☐ No<br><br>Relative exclusion criteria
Have you ever suffered from metabolic syndrome or diabetes?	☐ Yes ☐ No<br><br>Exclude if Yes
Have you ever suffered from any autoimmune condition (e.g. rheumatoid), asthma or eczema?<br>If yes, which, and do you take any treatment?	☐ Yes ☐ No<br><br>Relative exclusion criteria
Have you ever had any chronic pain or fatigue syndromes e.g.	☐ Yes ☐ No

For peer review only

chronic fatigue syndrome, fibromyalgia?<br>If yes, which?	Exclude if Yes
Have you any history of CJD or other prion disease in your family? If yes, please specify<br>Patients should be considered to be at risk from genetic forms of CJD if they have or have had<br>1. Genetic testing, which has indicated they are at significant risk of developing CJD or other prion disease<br>2. A blood relative known to have a genetic mutation indicative of genetic CJD or other prion disease<br>3. Two or more blood relatives affected by CJD or other prion disease	☐ Yes ☐ No<br><br>Exclude if Yes
Have you ever received growth hormone or gonadotrophic treatment? If yes, please specify;<br>i) Whether the hormone was derived from human pituitary glands<br>ii) The year of the treatment<br>iii) Whether the treatment was received in the UK or in another country<br><br>Recipients of hormone derived from human pituitary glands e.g. growth hormone or gonadotrophin have been identified as potentially at risk of CJD. In the UK, the use of human growth hormone was stopped in 1985 but human-derived products may have been continued to be used in other countries. In the UK, the use of human-derived gonadotrophin was discontinued in 1973 but may have been continued in other countries after this time.	☐ Yes ☐ No<br><br>Exclude if Yes

For peer review only - http://bmjopen.bmj.com/site/about/guidelines.xhtml

Have you ever had surgery on your brain or spinal cord?<br>People who underwent intradural neurosurgical or spinal procedures before August 1992 may have received a graft of human-derived dura mater and should be treated as at increased risk, unless evidence can be provided that human-derived dura mater was not used. Patients who received a graft of human-derived dura mater between 1980 and August 1992 are at increased risk of both sporadic CJD and vCJD.	☐ Yes ☐ No<br><br>Exclude if Yes
Are you normally resident in the UK?<br>If No state country of usual residence	☐ Yes ☐ No
Which countries have you visited in the last 12 months and what was the duration of stay?	Relative exclusions apply to tropical countries
In the past 12 months have you been admitted to a hospital in a country other than the UK?<br><br>If yes state when and which countries	☐ Yes ☐ No
Are you taking any regular medications?<br><br>List all current medications:	☐ Yes ☐ No<br><br>Exclude if any regular prescribed drugs (except OCP)

For peer review only

For peer review only

Reporting checklist for protocol of a clinical trial.

Based on the SPIRIT guidelines.

Instructions to authors

Complete this checklist by entering the page numbers from your manuscript where readers will find each of the items listed below.

Your article may not currently address all the items on the checklist. Please modify your text to include the missing information. If you are certain that an item does not apply, please write "n/a" and provide a short explanation.

Upload your completed checklist as an extra file when you submit to a journal.

In your methods section, say that you used the SPIRITreporting guidelines, and cite them as:

Chan A-W, Tetzlaff JM, Altman DG, Laupacis A, Gøtzsche PC, Krleža-Jerić K, Hróbjartsson A, Mann H, Dickersin K, Berlin J, Doré C, Parulekar W, Summerskill W, Groves T, Schulz K, Sox H, Rockhold FW, Rennie D, Moher D. SPIRIT 2013 Statement: Defining standard protocol items for clinical trials. Ann Intern Med. 2013;158(3):200-207

		Reporting Item	Page Number
**Administrative information**			
Title	#1	Descriptive title identifying the study design, population, interventions, and, if applicable, trial acronym	1
Trial registration	#2a	Trial identifier and registry name. If not yet registered, name of intended registry	2
Trial registration: data set	#2b	All items from the World Health Organization Trial Registration Data Set	4
Protocol version	#3	Date and version identifier	2
Funding	#4	Sources and types of financial, material, and other support	13
Roles and responsibilities: contributorship	#5a	Names, affiliations, and roles of protocol contributors	13

For peer review only

Roles and responsibilities: sponsor contact information	#5b	Name and contact information for the trial sponsor	13
Roles and responsibilities: sponsor and funder	#5c	Role of study sponsor and funders, if any, in study design; collection, management, analysis, and interpretation of data; writing of the report; and the decision to submit the report for publication, including whether they will have ultimate authority over any of these activities	13, 14
Roles and responsibilities: committees	#5d	Composition, roles, and responsibilities of the coordinating centre, steering committee, endpoint adjudication committee, data management team, and other individuals or groups overseeing the trial, if applicable (see Item 21a for data monitoring committee)	12
**Introduction**			
Background and rationale	#6a	Description of research question and justification for undertaking the trial, including summary of relevant studies (published and unpublished) examining benefits and harms for each intervention	3
Background and rationale: choice of comparators	#6b	Explanation for choice of comparators	4
Objectives	#7	Specific objectives or hypotheses	4
Trial design	#8	Description of trial design including type of trial (eg, parallel group, crossover, factorial, single group), allocation ratio, and framework (eg, superiority, equivalence, non-inferiority, exploratory)	5
**Methods: Participants, interventions, and outcomes**			
Study setting	#9	Description of study settings (eg, community clinic, academic hospital) and list of countries where data will be collected. Reference to where list of study sites can be obtained	7

For peer review only

Eligibility criteria	#10	Inclusion and exclusion criteria for participants. If applicable, eligibility criteria for study centres and individuals who will perform the interventions (eg, surgeons, psychotherapists)	6
Interventions: description	#11a	Interventions for each group with sufficient detail to allow replication, including how and when they will be administered	8
Interventions: modifications	#11b	Criteria for discontinuing or modifying allocated interventions for a given trial participant (eg, drug dose change in response to harms, participant request, or improving / worsening disease)	8
Interventions: adherance	#11c	Strategies to improve adherence to intervention protocols, and any procedures for monitoring adherence (eg, drug tablet return; laboratory tests)	8
Interventions: concomitant care	#11d	Relevant concomitant care and interventions that are permitted or prohibited during the trial	8
Outcomes	#12	Primary, secondary, and other outcomes, including the specific measurement variable (eg, systolic blood pressure), analysis metric (eg, change from baseline, final value, time to event), method of aggregation (eg, median, proportion), and time point for each outcome. Explanation of the clinical relevance of chosen efficacy and harm outcomes is strongly recommended	9
Participant timeline	#13	Time schedule of enrolment, interventions (including any run-ins and washouts), assessments, and visits for participants. A schematic diagram is highly recommended (see Figure)	9
Sample size	#14	Estimated number of participants needed to achieve study objectives and how it was determined, including clinical and statistical assumptions supporting any sample size calculations	11
Recruitment	#15	Strategies for achieving adequate participant enrolment to reach target sample size	7
**Methods: Assignment of interventions (for controlled trials)**			
Allocation: sequence generation	#16a	Method of generating the allocation sequence (eg, computer-generated random numbers), and list of any factors for stratification. To reduce predictability of a random sequence, details of any planned restriction (eg, blocking) should be	8

For peer review only

		provided in a separate document that is unavailable to those who enrol participants or assign interventions	
Allocation concealment mechanism	#16b	Mechanism of implementing the allocation sequence (eg, central telephone; sequentially numbered, opaque, sealed envelopes), describing any steps to conceal the sequence until interventions are assigned	8
Allocation: implementation	#16c	Who will generate the allocation sequence, who will enrol participants, and who will assign participants to interventions	8
Blinding (masking)	#17a	Who will be blinded after assignment to interventions (eg, trial participants, care providers, outcome assessors, data analysts), and how	8
Blinding (masking): emergency unblinding	#17b	If blinded, circumstances under which unblinding is permissible, and procedure for revealing a participant's allocated intervention during the trial	8
**Methods: Data collection, management, and analysis**			
Data collection plan	#18a	Plans for assessment and collection of outcome, baseline, and other trial data, including any related processes to promote data quality (eg, duplicate measurements, training of assessors) and a description of study instruments (eg, questionnaires, laboratory tests) along with their reliability and validity, if known. Reference to where data collection forms can be found, if not in the protocol	12
Data collection plan: retention	#18b	Plans to promote participant retention and complete follow-up, including list of any outcome data to be collected for participants who discontinue or deviate from intervention protocols	12
Data management	#19	Plans for data entry, coding, security, and storage, including any related processes to promote data quality (eg, double data entry; range checks for data values). Reference to where details of data management procedures can be found, if not in the protocol	12
Statistics: outcomes	#20a	Statistical methods for analysing primary and secondary outcomes. Reference to where other details of the statistical analysis plan can be found, if not in the protocol	12

For peer review only

Statistics: additional analyses	#20b	Methods for any additional analyses (eg, subgroup and adjusted analyses)	12
Statistics: analysis population and missing data	#20c	Definition of analysis population relating to protocol non-adherence (eg, as randomised analysis), and any statistical methods to handle missing data (eg, multiple imputation)	12
**Methods: Monitoring**			
Data monitoring: formal committee	#21a	Composition of data monitoring committee (DMC); summary of its role and reporting structure; statement of whether it is independent from the sponsor and competing interests; and reference to where further details about its charter can be found, if not in the protocol. Alternatively, an explanation of why a DMC is not needed	12
Data monitoring: interim analysis	#21b	Description of any interim analyses and stopping guidelines, including who will have access to these interim results and make the final decision to terminate the trial	12
Harms	#22	Plans for collecting, assessing, reporting, and managing solicited and spontaneously reported adverse events and other unintended effects of trial interventions or trial conduct	12
Auditing	#23	Frequency and procedures for auditing trial conduct, if any, and whether the process will be independent from investigators and the sponsor	12
**Ethics and dissemination**			
Research ethics approval	#24	Plans for seeking research ethics committee / institutional review board (REC / IRB) approval	13
Protocol amendments	#25	Plans for communicating important protocol modifications (eg, changes to eligibility criteria, outcomes, analyses) to relevant parties (eg, investigators, REC / IRBs, trial participants, trial registries, journals, regulators)	13
Consent or assent	#26a	Who will obtain informed consent or assent from potential trial participants or authorised surrogates, and how (see Item 32)	7

For peer review only

Consent or assent: ancillary studies	#26b	Additional consent provisions for collection and use of participant data and biological specimens in ancillary studies, if applicable	7
Confidentiality	#27	How personal information about potential and enrolled participants will be collected, shared, and maintained in order to protect confidentiality before, during, and after the trial	7
Declaration of interests	#28	Financial and other competing interests for principal investigators for the overall trial and each study site	14
Data access	#29	Statement of who will have access to the final trial dataset, and disclosure of contractual agreements that limit such access for investigators	12
Ancillary and post trial care	#30	Provisions, if any, for ancillary and post-trial care, and for compensation to those who suffer harm from trial participation	12
Dissemination policy: trial results	#31a	Plans for investigators and sponsor to communicate trial results to participants, healthcare professionals, the public, and other relevant groups (eg, via publication, reporting in results databases, or other data sharing arrangements), including any publication restrictions	12
Dissemination policy: authorship	#31b	Authorship eligibility guidelines and any intended use of professional writers	12
Dissemination policy: reproducible research	#31c	Plans, if any, for granting public access to the full protocol, participant-level dataset, and statistical code	12
**Appendices**			
Informed consent materials	#32	Model consent form and other related documentation given to participants and authorised surrogates	3
Biological specimens	#33	Plans for collection, laboratory evaluation, and storage of biological specimens for genetic or molecular analysis in the current trial and for future use in ancillary studies, if applicable	9

The SPIRIT checklist is distributed under the terms of the Creative Commons Attribution License CC-BY-ND 3.0. This checklist was completed on 26. March 2020 using https://www.goodreports.org/, a tool made by the EQUATOR Network in collaboration with Penelope.ai

For peer review only

BMJ Open

Protocol: A prospective, randomised placebo-controlled feasibility trial of Faecal microbiota Transplant to ERadicate gastrointestinal carriage of Antibiotic Resistant Organisms (FERARO)

Journal:	*BMJ Open*
Manuscript ID	bmjopen-2020-038847.R1
Article Type:	Protocol
Date Submitted by the Author:	03-Apr-2020

Complete List of Authors:	Merrick, Blair; Guy's and Saint Thomas' Hospitals NHS Trust, Centre for Clinical Infection and Diagnostics Research; King's College London Faculty of Life Sciences and Medicine<br>Robinson, Emily; King's College London, School of Population Health and Environmental Sciences<br>Bunce, Catey; King's College London, Primary Care and Public Health Sciences<br>Allen, Liz; Guy's and Saint Thomas' Hospitals NHS Trust; IQVIA Reading<br>BIsnauthsing, Karen; Guy's and Saint Thomas' Hospitals NHS Trust, Centre for Clinical Infection and Diagnostics Research<br>Izundu, Chi Chi<br>Bell, Jordana; King's College London Faculty of Life Sciences and Medicine<br>Amos, Gregory; National Institute for Biological Standards and Control<br>Shankar-Hari, Manu; Guy's and Saint Thomas' Hospitals NHS Trust, Intensive Care Unit; King's College London Faculty of Life Sciences and Medicine, Division of Asthma, Allergy and Lung Biology<br>Goodman, Anna; Guy's and Saint Thomas' NHS Foundation Trust, Centre for Clinical Infection and Diagnostics Research; King's College London Faculty of Life Sciences and Medicine<br>Shawcross, Debbie ; King's College London Faculty of Life Sciences and Medicine, Hepatology<br>Goldenberg, Simon; Guy's and Saint Thomas' NHS Foundation Trust, Centre for Clinical Infection and Diagnostics Research; King's College London Faculty of Life Sciences and Medicine

**Primary Subject Heading**:	Infectious diseases
Secondary Subject Heading:	Gastroenterology and hepatology
Keywords:	BACTERIOLOGY, Microbiology < BASIC SCIENCES, Infection control < INFECTIOUS DISEASES

For peer review only - http://bmjopen.bmj.com/site/about/guidelines.xhtml

I, the Submitting Author has the right to grant and does grant on behalf of all authors of the Work (as defined in the below author licence), an exclusive licence and/or a non-exclusive licence for contributions from authors who are: i) UK Crown employees; ii) where BMJ has agreed a CC-BY licence shall apply, and/or iii) in accordance with the terms applicable for US Federal Government officers or employees acting as part of their official duties; on a worldwide, perpetual, irrevocable, royalty-free basis to BMJ Publishing Group Ltd ("BMJ") its licensees and where the relevant Journal is co-owned by BMJ to the co-owners of the Journal, to publish the Work in this journal and any other BMJ products and to exploit all rights, as set out in our licence.

The Submitting Author accepts and understands that any supply made under these terms is made by BMJ to the Submitting Author unless you are acting as an employee on behalf of your employer or a postgraduate student of an affiliated institution which is paying any applicable article publishing charge ("APC") for Open Access articles. Where the Submitting Author wishes to make the Work available on an Open Access basis (and intends to pay the relevant APC), the terms of reuse of such Open Access shall be governed by a Creative Commons licence – details of these licences and which Creative Commons licence will apply to this Work are set out in our licence referred to above.

Other than as permitted in any relevant BMJ Author's Self Archiving Policies, I confirm this Work has not been accepted for publication elsewhere, is not being considered for publication elsewhere and does not duplicate material already published. I confirm all authors consent to publication of this Work and authorise the granting of this licence.

For peer review only

Protocol: A prospective, randomised placebo-controlled feasibility trial of **F**aecal microbiota Transplant to **ER**adicate gastrointestinal carriage of **A**ntibiotic **R**esistant **O**rganisms (FERARO)

Blair Merrick[1], Emily Robinson[2], Catey Bunce[2], Liz Allen[3, 4], Karen Bisnauthsing[1], Chi Chi Izundu[5], Jordana Bell[6], Gregory CA Amos[7], Manu Shankar-Hari[8], Anna Goodman[1], Debbie L Shawcross[9], Simon D Goldenberg[1 *]

Author affiliations

[1]Centre for Clinical Infection and Diagnostics Research, King's College, London and Guy's & St Thomas' NHS Foundation Trust, London, UK
[2] School of Population Health and Environmental Sciences, King's College London, UK
[3] Early Clinical Development Centre of Excellence, IQVIA, Reading UK
[4] Department of Pharmacy, Guy's and St Thomas' NHS Foundation Trust, London, UK
[5] Patient and Public Representative, London, UK
[6] Department of Twin Research and Genetic Epidemiology, King's College, London, UK
[7] National Institute for Biological Standards and Control, Potters Bar, Hertfordshire, UK
[8] Department of Infectious Diseases, School of Immunology and Microbial Sciences, Faculty of Life Sciences and Medicine, King's College, London and Guy's and St Thomas' NHS Foundation Trust, London, UK
[9] Institute of Liver Studies, Inflammation Biology, School of Immunology and Microbial Sciences, Faculty of Life Sciences and Medicine, King's College London, London, UK
* Corresponding author

Blair.merrick@gstt.nhs.uk
Emily.robinson@kcl.ac.uk
Catey.bunce@kcl.ac.uk
Liz.allen@iqvia.com
Karen.bisnauthsing@gstt.nhs.uk
Chi.chi.izundu@bbc.co.uk
Jordana.bell@kcl.ac.uk
Gregory.amos@nibsc.org
Manu.shankar-hari@kcl.ac.uk
Anna.goodman@gstt.nhs.uk
Debbie.shawcross@kcl.ac.uk
Simon.goldenberg@gstt.nhs.uk

Keywords: antimicrobial resistance, extended spectrum beta-lactamases, carbapenemase producing enterobacteriales, microbiome, faecal microbiota transplantation

Word count: 4142

For peer review only

Abstract

Introduction: antimicrobial resistance (AMR) is rising, largely due to the indiscriminate use of antimicrobials. The human gut is the largest reservoir of antibiotic resistant bacteria (ARB). Individuals colonised with ARB have the potential to spread these organisms both in the community and hospital settings. Infections with ARB such as extended spectrum beta-lactamase producing enterobacteriales (ESBL-E) and carbapenemase producing enterobacteriales (CPE) are more difficult to treat and are associated with an increased morbidity and mortality. Presently there is no effective decolonisation strategy for these ARB. Faecal microbiota transplant (FMT) has emerged as a potential strategy for decolonisation of ARB from the human gut, however there is significant uncertainty about the feasibility, effectiveness and safety of using this approach.
Methods and analysis: prospective, randomised, patient-blinded, placebo-controlled feasibility trial of FMT to eradicate gastrointestinal carriage of ARB. Eighty patients with a recent history of invasive infection secondary to ESBL-E or CPE and persistent gastrointestinal carriage will be randomised 1:1 to receive encapsulated FMT or placebo. The primary outcome measure is consent rate (as a proportion of patients who fulfil inclusion/ exclusion criteria); this will be used to determine if a substantive trial is feasible. Participants will be followed up at 1 week, 1 month, 3 months and 6 months and monitored for adverse events as well as gastrointestinal carriage rates of ARB after intervention.
Ethics and dissemination: research ethics approval was obtained by London - City & East Research Ethics Committee (ref 20/LO/0117). Trial results will be published in a peer-reviewed journal and presented at international conferences.
Trial registration number: ISRCTN registration number 34467677 and EudraCT number 2019-001618-41 protocol version 1.1. (dated 23/02/2020)

For peer review only

Strengths and limitations of this study

- The randomised, placebo-controlled design will control for spontaneous loss of carriage of resistant organisms
- Qualitative data from participant focus groups will inform and influence a potential future trial
- This study will assess feasibility; however it is not statistically powered to assess clinically efficacy, which will need to be evaluated in a substantive trial
- Mechanistic outcomes using metagenomic, metabolomic and host immune analyses could provide insight into the mechanism of action of FMT in treatment responders
- The lack of investigator blinding, and the single centre design is a limitation

Introduction

Antimicrobial resistance (AMR) in enterobacteriales is increasing, fuelled by the indiscriminate use of antimicrobials and inadequate infection control practices. Of greatest

For peer review only - http://bmjopen.bmj.com/site/about/guidelines.xhtml

concern are extended spectrum beta-lactamase producing (ESBL-E) and carbapenemase producing enterobacteriales (CPE). Rates of ESBL producing bacteria carriage in our local population are 9%, with the majority being CTX-M type (1). Rates of detection and infections caused by ESBL-E/ CRE are increasing nationally and globally (2, 3), resulting in a significant burden of attributable death and disability adjusted life years (4). Antimicrobial resistant bacteria (ARB) such as ESBL-E/ CPE have the capacity to spread between individuals and between organisms through horizontal gene transfer. These organisms have been responsible for several large and prolonged outbreaks worldwide (5-7). As well as increased morbidity and mortality, infection with resistant organisms is associated with prolonged hospital stay and increased healthcare costs (4, 8, 9). Hospitalised patients are particularly at risk of acquiring these organisms due to the treatments and procedures they receive, their comorbidities, and their high exposure to antimicrobials (5).

The microbiota of the human gut is a complex ecosystem and the largest reservoir of ARB (10, 11). A better understanding of the human microbiome has led to a new appreciation for the role indigenous microbes play in protecting us from invading exogenous pathogens. The role of the gut microbiota in defending the host against gastrointestinal pathogens was first described in a mouse model in which streptomycin administered orally to disrupt the gut microbiota resulted in increased rate of Salmonella enterica-related infections (12). Antimicrobials disrupt the balance of the delicate gut ecosystem, enabling colonisation by ESBL-E/ CPE and other potential pathogens. This is most strikingly evident in patients suffering from Clostridioides difficile infection (CDI), and the remarkable success of modulating this with faecal microbiota transplantation (FMT) (13, 14).

Attempts to control carriage of ARB in the gut using selective digestive decolonisation (SDD) are controversial, have not been widely adopted, and is not recommended by expert groups (15). Loss of ARB colonisation has been observed in a number of patients when using FMT to treat recurrent CDI (16). However, these reports are nearly all case series which are uncontrolled and do not account for spontaneous loss of carriage, which can occur in up to 50% of patients following hospital discharge (17).

The only published randomised trial of FMT to eradicate gastrointestinal carriage of ESBL-E and CPE was conducted in four academic centres in Geneva, Paris, Utrecht and Tel Aviv (18). Patients were randomised in a 1:1 ratio to a five-day course of colistin and neomycin followed by FMT or no intervention. The primary outcome measure was culture of ESBL-E / CPE from stool 35-48 days following randomisation, which was achieved for 41% (9/22) of patients in the intervention arm vs. 29% (5/17) in the control arm. Although the odds ratio for decolonisation success for FMT was 1.7 (95% confidence interval 0.4-6.4), this was not statistically significant, leaving the authors to conclude that the results do not support the routine use of FMT for decolonisation. Although the study was multicentre and included a control group to account for spontaneous loss of carriage, there are several limitations with the design and conduct, making it difficult to draw firm conclusions. Firstly, although designed as a superiority trial with a sample size calculation of 32 in each group, only 39 (61%) patients in total were randomised (due to recruitment problems). Secondly, patients in the intervention arm received five days of colistin and neomycin in addition to FMT, whereas the controls received no intervention. Thus, it is impossible to determine whether the results were due to the antibiotics (likely to have a profound effect on the gut flora) vs.

For peer review only - http://bmjopen.bmj.com/site/about/guidelines.xhtml

FMT. Thirdly, the methods of administration of FMT varied according to recruiting site; capsules were administered in two centres (16 patients), whilst two used nasogastric administration (6 patients). The capsules (15 administered each day over two days) were produced from one donation derived from 15-30g faeces. The nasogastric preparation was derived from 40g. There is evidence in the context of recurrent CDI that FMT preparations made with less than 50g faeces result in poorer outcomes than those made with more than this amount (19). Thus, a question exists over whether the patients were under dosed, and if repeated administrations (perhaps using different donors) might be more effective. Lastly, the study was not placebo controlled or blinded, although the primary outcome of stool culture at one month is fairly objective, there is the possibility of introducing bias in an investigator who is aware of the allocation.

Due to the limitations of the above study, the lack of other rigorously conducted, well controlled studies, and the considerable doubt that sufficient patients would be willing to participate in research of this type, we designed a feasibility study to address some of the outstanding questions.

Methods and analysis

Primary objectives

The primary objective of this study is to determine the feasibility and acceptability of administering encapsulated FMT to participants colonised with ESBL-E / CPE. This will be used to determine if a substantive trial is feasible.

Primary endpoints

The primary outcome measure is consent rate (as a proportion of patients who fulfil inclusion/ exclusion criteria). The success criteria for the primary endpoint are stratified. If <15% is achieved, progression to a substantive trial will not be deemed feasible. If 15-39%, progression to a substantive trial will be deemed feasible with protocol modifications and clearly defined stop/go criteria. An overall consent rate of >40% will be taken as indicating a substantive trial is feasible.

Secondary objectives

The secondary objectives are to assess other feasibility aspects of conducting a substantive trial, to evaluate the safety and tolerability of FMT in this patient population, and to provide early evidence of efficacy. These measures should inform a future trial, such as determining the primary (efficacy) outcome and sample size, if progression criteria are met. A full list of criteria for progression to a substantive trial are details in Table 1 of supplementary file.

Secondary feasibility endpoints

- Proportion of patients fulfilling inclusion / exclusion criteria
- Proportion of patients receiving FMT / placebo (as a % of those consenting)
- Proportion of patients returning for follow up visits (face to face visit at Day 40)
- Proportion of patients providing follow up stool samples (Days 10, 40, 100 and 190)
- Ability to recruit sufficient healthy donors to manufacture all FMT doses to meet demands of this and a future substantive RCT. Assessed by delay in dosing patients (measured in days)

For peer review only - http://bmjopen.bmj.com/site/about/guidelines.xhtml

Additional feasibility assessments will include:
• Collection of data that may be used in estimating of costs/resources needed to provide FMT in the NHS.
• An embedded qualitative study to explore views and experiences of research participants.

Secondary efficacy endpoints
• Gastrointestinal carriage of CRE / ESBL (detected / not detected) by stool culture over time (days 10, 40, 100 and 190)
• Gastrointestinal carriage of CRE / ESBL (detected / not detected) by multiplex PCR over time (days 10, 40, 100 and 190)

Secondary safety and tolerability endpoints
• proportion of patients experiencing reflux following administration of FMT
• Proportion of patients suffering intolerable (resulting in withdrawal from the study) gastrointestinal side effects (including diarrhoea, constipation, abdominal pain, flatulence and bloating). This will be assessed by direct questioning and completion of a short patient questionnaire.
• identification of unanticipated harms involved with administration of FMT.
• occurrence of any adverse event / serious adverse event

Exploratory endpoints/outcomes
The following exploratory / mechanistic outcomes will be measured:

• Changes in the gut microbiome induced by capsulized FMT as measured by comparing between treatment groups change (relative to baseline) in;
- the proportion and relative abundance of bacterial taxa over time (days 10, 40, 100 and 190)
- the change in diversity of the microbiome over time (days 10, 40, 100 and 190) measured using Shannon and Simpson indices
- antibiotic resistance genes carriage over time (days 10, 40, 100 and 190)

• Changes in the gut metabolome induced by capsulized FMT (using Nuclear Magnetic Resonance (NMR) spectroscopy). Measured at days 10, 40, 100 and 190.

• Host immune response T and B cell) as measured by comparing participants prior to and day 40, as well as donors who will act as controls.

Trial design
Randomised control participant-blinded, single-centre, feasibility trial with two parallel groups (FMT capsules and matched placebo). Eighty patients will be randomised 1:1 (40 will receive FMT capsules and 40 placebo) from eligible patients identified from Guy's and St Thomas' hospitals (figure 1 & table 1).

For peer review only - http://bmjopen.bmj.com/site/about/guidelines.xhtml

Table 1: Participant inclusion/exclusion criteria

Inclusion criteria

To be eligible for enrolment, a participant must meet all the following criteria before undergoing any study-related procedures:

- Adult patients (age 18 years or older at time of consent) **AND**
- Current/ previous patient at Guy's and St Thomas' NHS Foundation Trust **AND**
- Ability to understand the purpose, potential benefits and risks of the study and capable of giving informed consent. The participant must be able to provide written informed consent **AND**
- Documented gastrointestinal carriage of ESBL-E or CPE (stool sample) in the 21 days prior to consent **AND**
- Symptomatic infection with the same target organism of interest in preceding 6 months (this needs to be microbiologically confirmed but is not restricted to any particular body site e.g. could be urinary tract infection, intra-abdominal infection, blood stream infection etc).

Exclusion criteria

- Pregnancy or planned pregnancy
- Breastfeeding
- Severe or life-threatening food allergy
- Allergy or other contraindication to omeprazole, IMP or placebo ingredients
- Treatment with systemic antibiotic on the day of and day prior to 1st IMP/placebo dosing to the end of the dosing period
- Treatment with pre or probiotics in the 4 weeks prior to randomisation and for the duration of the study
- Severe immunodeficiency:
  - systemic chemotherapy <30 days from baseline or planned chemotherapy within the upcoming 6 months
  - Known HIV infection with CD4 count <250 cells/uL
  - Known neutropenia with absolute neutrophils <$1.0x10^9$
  - Prolonged treatment with corticosteroids (equivalent to prednisone >60mg daily for > 30 days) within 8 weeks of randomisation
- Life expectancy <6 months
- Swallowing disorder, oral-motor dyscoordination or likely inability/unwillingness to ingest study medication
- Patients who have received another investigational drug or device within 4 months prior to randomisation
- Any condition or circumstance, in the opinion of the investigator, that would compromise the safety of the patient or the quality of the study data

Patient and public involvement

Patients and the public have identified antimicrobial resistance as a research priority and were involved in identifying the research question and providing feedback on the grant application.

For peer review only

A patient representative has been appointed to the Trial Steering Committee (TSC), and has advised on the design of the research, the protocol and all patient facing materials. The patient representative will also be involved in dissemination of the study findings. As the acceptability of FMT in this setting is a key research question we will invite up to eight patients to participate in focus groups. The aim of the group will be to understand their experience in participation in the study and will focus on acceptability, barriers to participation and improvements that could be made to any resulting substantive trial.

Patient population

Participants will be recruited from Guy's and St Thomas' NHS Foundation Trust, a 1200 bed academic centre in central London. It is anticipated that most patients will already be admitted to the hospital as part of standard of care treatment, thus, most activities will take place on the ward or clinical area that the patient is already located. Where this is not the case, participants will be invited to attend the infection clinical room on an outpatient basis.

Consent

Informed consent (for both healthy donors and patient-recipients) will be obtained prior to any trial related activities, including screening for eligibility. Potential participants will be given the participant information sheet (PIS) and allowed enough time to read thoroughly and discuss with others outside of the study team (e.g. family, friends, general practitioner) (see online supplementary file table 2). Participants are free to withdraw from the trial at any time without giving reasons. Data and samples collected up to the point of withdrawal will only be used after withdrawal if the participant consented for this. Patients who lack capacity will not be enrolled in this study. Where a participant consents but later becomes incapacitated, the original consent given endures the loss of capacity, providing that the trial has not significantly altered.

Randomisation

The randomisation schedule will be generated using a validated online randomisation programme, hosted by King's Clinical Trials Unit. The method of randomisation will be block randomisation with randomly varying block sizes. As this is a single centre study, randomisation does not need to be stratified. Participants will be allocated treatment as close as possible to receiving it.

Study intervention

FMT for this trial will be prepared in a lyophilised, encapsulated form in accordance with Good Manufacturing Practice (GMP) principles and under manufacturing authorisation for an Investigational Medicinal Product (IMP) from the Medicines and Healthcare Products Regulatory Agency (MHRA). Our centre has recently provided FMT for a CTIMP for cirrhosis and this follows similar processes (20). Healthy donor inclusion and exclusion criteria and screening and eligibility questionnaire are described in Tables 3 and 4 of the supplementary file.

The product contains 0.9% sodium chloride and 5% trehalose (cryoprotectant) as excipients. A minimum of 80g faeces from each donor will be used to manufacture one batch of five capsules. Following lyophilisation, the material will be encapsulated in five size 0 delayed release methylcellulose capsules (DRcaps™, Capsugel®, Livingston, UK). Placebo capsules

For peer review only - http://bmjopen.bmj.com/site/about/guidelines.xhtml

will contain microcrystalline cellulose. The capsules for the FMT and placebo will be identical in appearance. The capsules are coloured Swedish orange, resulting in an opaque appearance through which the contents cannot be seen.

FMT donors are carefully screened healthy volunteers with a body mass index between 18-30. Donors undergo questionnaire screening for risk factors and testing for a range of infectious agents as previously described and in accordance with national guidelines (see appendix A for full details) (21). FMT material is traceable from donor to recipient. Aliquots of donor stool will be kept for 30 years to allow for future testing if required.

At baseline participants will have their medication history recorded, including over the counter preparations and supplements as well as pre/probiotics. Vital signs, height and weight, and baseline blood biochemistry and haematology will be collected. Additionally, a serum sample will be stored to allow future testing in the event of a possible transmission event. If female and of child-bearing age a urinary pregnancy test will be performed. An EQ-5D questionnaire will also be administered.

Encapsulated FMT (IMP) and placebo will be dispensed by study staff to trial participants over three consecutive days (or over five days if over a weekend). Patients will be fasted for 4 hours and be pre-treated with omeprazole on the morning on the FMT (40mg on first dosing day and 20mg on the two subsequent dosing days).
It is anticipated that most patients will remain an inpatient for the duration of treatment. If they have been discharged in the interim provision will be made for them to attend for treatment as an outpatient.

Evaluations during and after treatment
Follow up events will be scheduled for all participants at 1 week, 1 month, 3 months and 6 months after end of therapy. If an outpatient, the visits at 1 week, 3 months and 6 months will be conducted by telephone, with the participant returning a stool sample by post. The follow-up at 1 month will be face to face and will include a blood sample for immune analyses. All visits will involve completion of an EQ-5D questionnaire (see figure 2 for additional details).

Sample analyses
Stool samples will be analysed for the presence of ESBL-E/ CPE using culture based (chromogenic agar with species identification using MALDI-ToF mass spectrometry and phenotypic antimicrobial susceptibility testing) and molecular techniques (multiplex PCR panel for 16 ESBL/ CPE resistance genes).

Follow-up
If a participant fails to present for follow up assessment, all attempts to contact the participant and information received during contact attempts will be documented in the participant's medical record. In any circumstance, every effort will be made to contact the participant and document outcome (i.e. three documented contact attempts via phone calls, on separate occasions will be made to locate or contact the participant, and/or determine health status). Stool samples will be stored for further follow-on analysis, including metagenomics and metabolomics profiling.

For peer review only - http://bmjopen.bmj.com/site/about/guidelines.xhtml

Qualitative study

A qualitative study of participant's experiences will be undertaken and comprises a focus group interview with a minimum of eight participants. Ideally the group will include at least two patients who were approached but did not agree to participate. The aim of these discussions is to identify facilitators and barriers to delivering the trial, and whether there are any aspects of the trial that should be changed. The interviews will be semi-structured and recorded to aid writing up the study report. Objectives of the focus group will include; identifying ways of increasing recruitment and retention; identifying ways of broadening participation in the trial to improve diversity of population; improving understanding of how participants join trials and experience of participation; measuring reasons for non-adherence to the trial medication; exploring stakeholders' views of acceptability of the trial design; strengthening the ethical conduct of the trial, for example, informed consent procedures; addressing any local issues which may impact on the feasibility of a substantive trial.

Statistical analysis

Sample size

As this is a feasibility study, significance tests between or within groups will not be performed for the study's primary and secondary endpoints, therefore a power calculation has not been performed. For feasibility and pilot studies, sample sizes between 24 and 50 have been recommended to estimate a chosen parameter (22, 23). We have chosen a 1:1 treatment to placebo ratio, therefore a total sample size of 80 would be enough to estimate the standard deviation of the outcome in 40 treated patients, allowing for some loss to follow-up. We will also be able to estimate our expected recruitment rate of 40% (95% CI: 33-47) if we approach around 200 eligible patients.

Data synthesis, analysis and presentation

A Statistical Analysis Plan will be written by the trial statistician and signed off prior to database lock. The study will be reported in accordance with the CONSORT extension for pilot and feasibility studies.

The proportion of patients who accept the offer of randomisation will be reported with 95% confidence intervals computed by the exact binomial method. No statistical tests for significant differences between treatment groups will be performed. In addition to summary statistics of the secondary outcomes, all harms and withdrawals will be reported with 95% confidence intervals. Patients will be analysed in the groups to which they are randomised in accordance with intent to treat principals.

The protocol has been designed to place minimal burden upon patients and case report forms are only capturing essential data. It is inevitable that there may be some missing data, which will be reported by treatment group with reasons for missingness described, where possible. Since this is a feasibility study, we do not plan to impute missing data.

Statistical software

For peer review only

All statistical analysis will be conducted using Stata version 15.0 or above (StatCorp, Texas).

Trial monitoring groups

Trial Management Group (TMG)
Comprises the chief investigator (CI), trial statistician, trial staff and other lead clinical and non-clinical co-investigators and co-applicants. The TMG are responsible for the day-to-day management of the trial and to ensure all practical details of the trial are progressing and working well. The TMG will monitor all aspects of the conduct and progress of the trial, ensuring that the protocol is adhered to and take appropriate action to safeguard participants and the quality of the trial itself. The TMG will be responsible for drafting of the final report and submission for publication.

Trial Steering Committee (TSC)
A Trial Steering Committee (TSC) will be convened with membership nominated by the CI in partnership with the sponsor. The role of the TSC is to provide overall supervision for the trial on behalf of the sponsor and funder and to ensure that the project is conducted to the rigorous standards set out in the Department of Health and Social Care's Research Governance Framework for Health and Social Care and the Guidelines for Good Clinical Practice. The committee Chair will be independent of the study. The committee will also comprise four other independent members (Consultant Microbiologists or Gastroenterologists) a patient/public representative, and an independent statistician.
The TSC will take responsibility for monitoring data and making recommendations to the TMG on whether there are any ethical or safety reasons why the trial should not continue. A separate Data Monitoring and Ethics Committee (DMEC) will not be established as this is a single centre feasibility trial with a relatively small number of patients using an established IMP with a relatively well described safety profile.

Ethics and dissemination
Research ethics approval was obtained by London - City & East Research Ethics Committee (ref. 20/LO/0117). Trial results will be published in a peer-reviewed journal and presented at international conferences.

Discussion

Several case reports and one randomised controlled trial of ARB decolonisation using FMT are summarised in four systematic reviews (16, 24-26). Most studies were case reports or case series which did not control for spontaneous loss of ARB carriage. This is important since it may be significant and may lead to overestimation of the effectiveness of FMT in achieving decolonisation. In a recent study conducted at Central Manchester Foundation Trust during 2016/17 only 17.1% of patients who were previously known to be colonised with CRE had it detected on readmission to the hospital (27). Therefore, the use of a placebo in this trial is justified and crucial to control for spontaneous loss of carriage.

Capsule administration has been selected following consultation with patient groups. It is more acceptable and cost effective than other methods of administration such as via nasojejunal tube.

For peer review only

Although the underlying mechanism of action of FMT is not fully elucidated, the use of three different donors is justified as it likely increases the bacterial diversity in the administered IMP, with the hope that this will engraft in the recipient. The previous study using a single donor resulted in an odds ratio for decolonisation success of 1.7 (95% CI 0.4-6.4). We hypothesize that using multiple donors at three dosing points will result in a higher rate of decolonisation. This is also based on experience of using FMT to treat patients with ulcerative colitis, where multiple donors are used in prolonged treatment intervals of up to six weeks (28-31).

The overall aim of this programme of work (which would proceed to a future substantive RCT if feasible) is to eradicate or suppress ESBL-E / CRE without resorting to the use of antibiotics. If that can be achieved, then the risk of an invasive infection with ARB in these patients could be significantly reduced.

Acknowledgements
EJR receives salary support from the NIHR Biomedical Research Centre based at Guy's and St Thomas' NHS Foundation Trust and King's College, London

Collaborators
Annapurna Vyakarnam, David Moyes, James Mason

Contributors
SDG and BM conceived and designed the trial, and drafted the manuscript.
EJR and CB designed the statistical aspects of the study.
MSH designed the exploratory immunology aspects of the study.
GCAA and JB designed exploratory microbiome aspects of the study.
LA provided expert advice on regulatory and compliance aspects of the study.
DLS is Chair of the Trial Steering Committee and provided expert advice.
CCI provided input from the patient and public perspective.
KB provided advice on sample collection and reviewed all participant materials.
AG provided general expert advice on the design and conduct of the study.
All authors reviewed and approved the final manuscript.

Sponsor
The study sponsor is Guy's and St Thomas' NHS Foundation Trust, Great Maze Pond, London SE1 9RT. The sponsor had no role in the design of this study and will not have any role during its execution, analyses, interpretation of the data, or decision to submit results.

Funding
This independent research is supported by the National Institute for Health Research (NIHR) under its Research for Patient Benefit (RfPB) Programme (grant reference number PB-PG-0418-20007). The funder had no role in the design of this study and will not have any role during its execution, analyses, interpretation of the data, or decision to submit results.

Competing interests
SDG has received personal fees from Astellas, Enterobiotix, Menarini, MSD, Pfizer and Shionogi.
DLS has undertaken paid consultancy for Norgine Ltd, Shionogi and Kaleido Biosciences and paid lectures for Falk Pharma, Norgine Ltd and Alfa Sigma
All other authors report no competing interests.

Patient consent for publication
Not required.

For peer review only

Ethics approval
Research ethics approval was given by London - City & East Research Ethics Committee (ref. 20/LO/0117).

For peer review only

References

1. J. A. Otter, A. Natale, R. Batra, O. Tosas Auguet, E. Dyakova, S. D. Goldenberg, et al. Individual- and community-level risk factors for ESBL Enterobacteriaceae colonization identified by universal admission screening in London. Clin Microbiol Infect. 2019;25(10):1259-65.
2. Public Health England. English Surveillance Programme for Antimicrobial Utilisation and Resistance (ESPAUR) Report. 2019.
3. V. Gupta, G. Ye, M. Olesky, K. Lawrence, J. Murray, K. Yu. Trends in resistant Enterobacteriaceae and Acinetobacter species in hospitalized patients in the United States: 2013-2017. BMC Infect Dis. 2019;19(1):742.
4. A. Cassini, L. D. Hogberg, D. Plachouras, A. Quattrocchi, A. Hoxha, G. S. Simonsen, et al. Attributable deaths and disability-adjusted life-years caused by infections with antibiotic-resistant bacteria in the EU and the European Economic Area in 2015: a population-level modelling analysis. Lancet Infect Dis. 2019;19(1):56-66.
5. K. van Loon, A. F. Voor In 't Holt, M. C. Vos. A Systematic Review and Meta-analyses of the Clinical Epidemiology of Carbapenem-Resistant Enterobacteriaceae. Antimicrob Agents Chemother. 2018;62(1).
6. L. Tavoschi, S. Forni, A. Porretta, L. Righi, F. Pieralli, F. Menichetti, et al. Prolonged outbreak of New Delhi metallo-beta-lactamase-producing carbapenem-resistant Enterobacterales (NDM-CRE), Tuscany, Italy, 2018 to 2019. Euro Surveill. 2020;25(6).
7. V. Decraene, H. T. T. Phan, R. George, D. H. Wyllie, O. Akinremi, Z. Aiken, et al. A Large, Refractory Nosocomial Outbreak of Klebsiella pneumoniae Carbapenemase-Producing Escherichia coli Demonstrates Carbapenemase Gene Outbreaks Involving Sink Sites Require Novel Approaches to Infection Control. Antimicrob Agents Chemother. 2018;62(12).
8. H. Suzuki, E. N. Perencevich, D. J. Livorsi, B. Alexander, B. F. Beck, K. K. Richardson, et al. Attributable mortality due to fluoroquinolone and extended-spectrum cephalosporin resistance in hospital-onset Escherichia coli and Klebsiella spp bacteremia: A matched cohort study in 129 Veterans Health Administration medical centers. Infect Control Hosp Epidemiol. 2019;40(8):928-31.
9. C. E. French, C. Coope, L. Conway, J. P. Higgins, J. McCulloch, G. Okoli, et al. Control of carbapenemase-producing Enterobacteriaceae outbreaks in acute settings: an evidence review. J Hosp Infect. 2017;95(1):3-45.
10. C. J. Donskey. The role of the intestinal tract as a reservoir and source for transmission of nosocomial pathogens. Clin Infect Dis. 2004;39(2):219-26.
11. J. Carlet. The gut is the epicentre of antibiotic resistance. Antimicrob Resist Infect Control. 2012;1(1):39.
12. M. Bohnhoff, C. P. Miller, W. R. Martin. Resistance of the Mouse's Intestinal Tract to Experimental Salmonella Infection. Factors Which Interfere with the Initiation of Infection by Oral Inoculation. J Exp Med. 1964;120:805-16.
13. E. van Nood, M. G. Dijkgraaf, J. J. Keller. Duodenal infusion of feces for recurrent Clostridium difficile. N Engl J Med. 2013;368(22):2145.
14. P. Moayyedi, Y. Yuan, H. Baharith, A. C. Ford. Faecal microbiota transplantation for Clostridium difficile-associated diarrhoea: a systematic review of randomised controlled trials. Med J Aust. 2017;207(4):166-72.

For peer review only - http://bmjopen.bmj.com/site/about/guidelines.xhtml

15. E. Tacconelli, F. Mazzaferri, A. M. de Smet, D. Bragantini, P. Eggimann, B. D. Huttner, et al. ESCMID-EUCIC clinical guidelines on decolonization of multidrug-resistant Gram-negative bacteria carriers. Clin Microbiol Infect. 2019;25(7):807-17.
16. S. Saha, R. Tariq, P. K. Tosh, D. S. Pardi, S. Khanna. Faecal microbiota transplantation for eradicating carriage of multidrug-resistant organisms: a systematic review. Clin Microbiol Infect. 2019;25(8):958-63.
17. J. P. Vink, J. A. Otter, J. D. Edgeworth. Carbapenemase-producing Enterobacteriaceae - once positive always positive? Curr Opin Gastroenterol. 2020;36(1):9-16.
18. B. D. Huttner, V. de Lastours, M. Wassenberg, N. Maharshak, A. Mauris, T. Galperine, et al. A 5-day course of oral antibiotics followed by faecal transplantation to eradicate carriage of multidrug-resistant Enterobacteriaceae: a randomized clinical trial. Clin Microbiol Infect. 2019;25(7):830-8.
19. E. Gough, H. Shaikh, A. R. Manges. Systematic review of intestinal microbiota transplantation (fecal bacteriotherapy) for recurrent Clostridium difficile infection. Clin Infect Dis. 2011;53(10):994-1002.
20. C. A. Woodhouse, V. C. Patel, S. Goldenberg, A. Sanchez-Fueyo, L. China, A. O'Brien, et al. PROFIT, a PROspective, randomised placebo controlled feasibility trial of Faecal mIcrobiota Transplantation in cirrhosis: study protocol for a single-blinded trial. BMJ Open. 2019;9(2):e023518.
21. S. D. Goldenberg, R. Batra, I. Beales, J. L. Digby-Bell, P. M. Irving, L. Kellingray, et al. Comparison of Different Strategies for Providing Fecal Microbiota Transplantation to Treat Patients with Recurrent Clostridium difficile Infection in Two English Hospitals: A Review. Infect Dis Ther. 2018;7(1):71-86.
22. S. A. Julious. Two-sided confidence intervals for the single proportion: comparison of seven methods by Robert G. Newcombe, Statistics in Medicine 1998; 17:857-872. Stat Med. 2005;24(21):3383-4.
23. J. Sim, M. Lewis. The size of a pilot study for a clinical trial should be calculated in relation to considerations of precision and efficiency. J Clin Epidemiol. 2012;65(3):301-8.
24. M. H. Woodworth, M. K. Hayden, V. B. Young, J. H. Kwon. The Role of Fecal Microbiota Transplantation in Reducing Intestinal Colonization With Antibiotic-Resistant Organisms: The Current Landscape and Future Directions. Open Forum Infect Dis. 2019;6(7).
25. V. Tavoukjian. Faecal microbiota transplantation for the decolonization of antibiotic-resistant bacteria in the gut: a systematic review and meta-analysis. J Hosp Infect. 2019;102(2):174-88.
26. B. Davido, R. Batista, A. Dinh, P. de Truchis, E. M. Terveer, B. Roberts, et al. Fifty shades of graft: How to improve the efficacy of faecal microbiota transplantation for decolonization of antibiotic-resistant bacteria. Int J Antimicrob Agents. 2019;53(5):553-6.
27. A. Tucker, R. George, W. Welfare, P. Cleary, J. Cawthorne, A. Dodgson. Screening for carbapenemase-producing Enterobacteriaceae in previous carriers readmitted to hospital: evaluation of a change in screening policy. J Hosp Infect. 2019;103(2):156-9.
28. P. Moayyedi, M. G. Surette, P. T. Kim, J. Libertucci, M. Wolfe, C. Onischi, et al. Fecal Microbiota Transplantation Induces Remission in Patients With Active Ulcerative Colitis in a Randomized Controlled Trial. Gastroenterology. 2015;149(1):102-9 e6.
29. N. G. Rossen, S. Fuentes, M. J. van der Spek, J. G. Tijssen, J. H. Hartman, A. Duflou, et al. Findings From a Randomized Controlled Trial of Fecal Transplantation for Patients With Ulcerative Colitis. Gastroenterology. 2015;149(1):110-8 e4.

For peer review only

30. S. Paramsothy, M. A. Kamm, N. O. Kaakoush, A. J. Walsh, J. van den Bogaerde, D. Samuel, et al. Multidonor intensive faecal microbiota transplantation for active ulcerative colitis: a randomised placebo-controlled trial. Lancet. 2017;389(10075):1218-28.
31. S. P. Costello, O. Waters, R. V. Bryant, R. Katsikeros, J. Makanyanga, M. Schoeman, et al. Short Duration, Low Intensity, Pooled Fecal Microbiota Transplantation Induces Remission in Patients with Mild-Moderately Active Ulcerative Colitis: A Randomised Controlled Trial. Gastroenterology. 2017;152(5):S198-S9.

Figure legends:

Table 1. Participant inclusion/exclusion criteria
Figure 1. CONSORT flow diagram
Figure 2. Intervention and follow up

For peer review only

Figure 1: CONSORT flow diagram

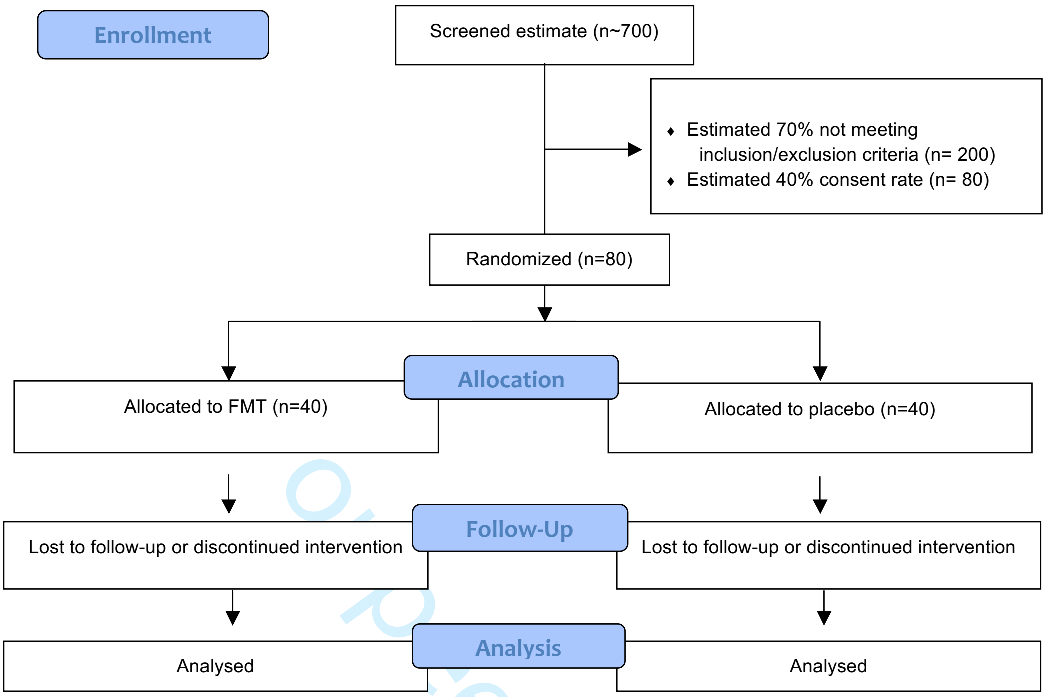

For peer review only

Figure 2: Intervention and follow-up

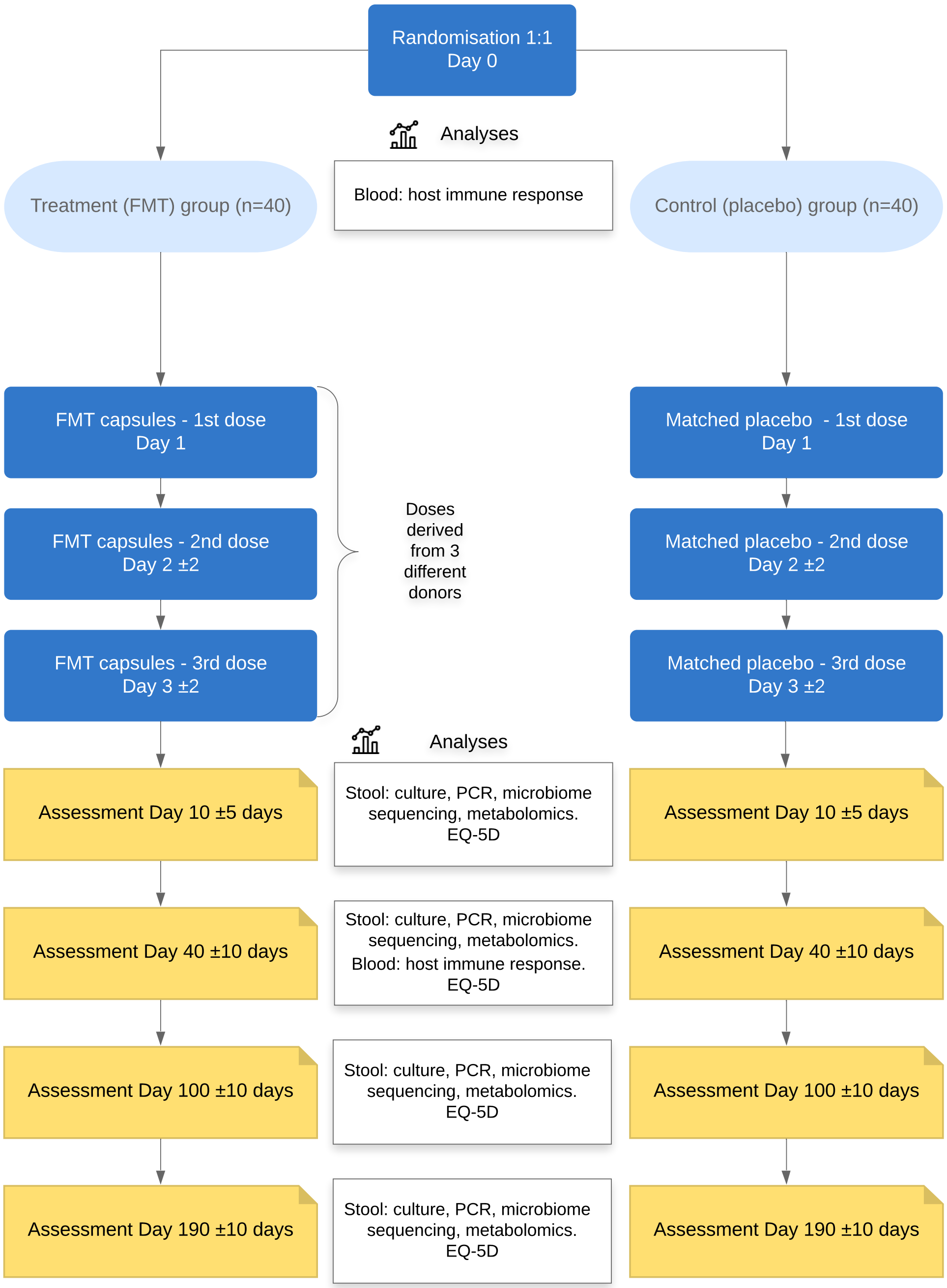

Supplementary file S1.

FERARO: A prospective, randomised placebo-controlled feasibility trial of **F**aecal microbiota Transplant to **ER**adicate gastrointestinal carriage of **A**ntibiotic **R**esistant **O**rganisms: study protocol for single-blinded trial

Table 1. Criteria for progression to a substantive trial

Criteria	Stop – substantive trial not feasible	Continue with protocol modifications, close monitoring and clearly defined stop/go points	Continue without modification – feasible as is
**Hard Feasibility Criteria<br>Used to determine progression to a substantive trial**			
Consent rate (% of patients who fulfil eligibility criteria)	<15%	15-39%	>40%
**Soft Feasibility Criteria<br>Taken into consideration in determining progression to a substantive trial**			
Proportion of patients fulfilling eligibility criteria	<10%	10-29%	>30%
% of patients receiving IMP/placebo (as % of those consenting) and compliant will all doses on all three days	<50%	50-75%	>75%
% of patients returning for follow up visit (Day 40)	<50%	50-79%	>80%
% of patients providing follow up stool samples (Days 10, 40, 100 and 190)	<50% returning two or more samples	50-79% patients returning two or more samples	>80% patients returning two or more samples
Ability to recruit sufficient healthy donors to manufacture all FMT	Delay in dosing patients >2 weeks	Delay in dosing patients up to 2 week2	No delay in patient dosing
**Soft Patient Tolerability Criteria<br>Taken into consideration in determining progression to a substantive trial**			
% of patients experiencing reflux	>51%	21-50%	<20 %

For peer review only - http://bmjopen.bmj.com/site/about/guidelines.xhtml

following FMT administration			
Intolerable (resulting in withdrawal) side effects	>51%	21-50%	<20 %

For peer review only

Table 2. Participant Information Sheet (PIS)

A prospective, randomised placebo controlled feasibility trial of **F**aecal microbiota Transplant to **ER**adicate gastrointestinal carriage of **A**ntibiotic **R**esistant **O**rganisms

The FERARO Trial

Patient Information Sheet

We would like to invite you to take part in the FERARO trial.

- Before you decide whether you would like to take part in the trial, it's important for you to understand why the research is being done and what it would involve.
- Please take some time to read the information carefully, and discuss with your family, friends and doctor, as you wish.
- Ask us if anything is not clear, or you would like some more information

Important things that you need to know:

- Taking part is completely up to you and you can stop taking part at any time, without giving a reason. If you do not wish to take part, this will not affect the care you receive from your doctors or other health care professionals.
- You have been asked to take part in this study because a sample you have provided contains Antibiotic Resistant Bacteria (ARB). This study is testing the acceptability of a treatment that may be able to reduce the amount of ARB in the gut and the risk of infection.
- Faecal Microbiota Transplant (FMT) is a capsule made up of bacteria taken from a stool (poo) sample donated by healthy people. It could help to restore the balance of bacteria in the gut by reducing the amount of bacteria that are resistant to antibiotics.
- Faecal Microbiota Transplant (FMT) is a treatment used currently to treat patients with repeated Clostridioides difficile (C. diff) infection.
- The FERARO trial is looking to see if you consider this to be an acceptable treatment and if there are any side effects.

For peer review only - http://bmjopen.bmj.com/site/about/guidelines.xhtml

- You will be allocated to receive either FMT capsules or a placebo (dummy) capsules. You will not know which treatment you receive.
- You will be asked to take five capsules a day for three days in a row. This may be while you are already staying in hospital or as an outpatient.
- You will be asked to provide stool samples before and after receiving treatment to see if there are any changes in the bacteria present in your gut.
- You will be followed up by the study team for six months after completing the treatment.

If you have any questions about this study, please contact:

Dr Simon Goldenberg
Telephone (with message facility): 020 7188 8515
Email: simon.goldenberg@gstt.nhs.uk

What is the purpose of the study?

The human gut has trillions of good bacteria (germs or bugs) which are important to keep us healthy. In total these bugs are called the microbiota. The bugs are always evolving to beat antibiotics used to fight them and this is known as resistance. Resistance to antibiotics allows bugs to survive and spread.

Antibiotic resistant bacteria (ARB) usually live in the gut (or in the surrounding environment), where they do no harm. This is called colonisation. However, the ARB can appear and cause infection in other parts of the body that normally lack any bacteria, for example in the bladder or blood. When this happens, treatment with a more powerful type of antibiotic is usually needed. This is more likely to happen in people who are more prone to infection, including people with an underlying disease or injury, or people who are already admitted to hospital.

Antibiotic resistance is a growing and serious threat to worldwide health, and means that doctors may be limited in the types of treatments that they can offer to patients. Without effective antibiotics even simple infections could become deadly, making routine medical procedures too dangerous to perform. There is an urgent need to find new antibiotics, but this takes time and is very expensive.

Two particular groups of antibiotic resistant bacteria are known as CRE and ESBL (Carbapenem Resistant Enterobacteriales and Extended Spectrum Beta-lactamase producing bacteria).

Carbapenems and beta-lactams are some of the most powerful types of antibiotics. Some strains of bacteria make enzymes (chemicals), which allow them to destroy carbapenem and beta-lactam antibiotics which makes the bacteria resistant to the antibiotics.

For peer review only - http://bmjopen.bmj.com/site/about/guidelines.xhtml

There is growing interest in non-antibiotic treatments like Faecal Microbiota Transplant (FMT) to deal with this problem.

FMT is the transfer of bacteria from the guts of healthy donors (taken from their poo / stools) into the gut of a patient. The aim is to restore a healthy balance of bacteria (reducing harmful ones and increasing good ones). It is currently used to treat patients with repeated Clostridioides difficile infection. This is an infection causing severe diarrhoea and stomach pain, normally after having antibiotics which have harmed the microbiota.

FMT is very effective and safe in treating this group of patients, with success rates of over 80%. Initial research shows that it may be helpful in other conditions.

Why have I been invited to take part?

You have been identified by a member of your healthcare team as a carrier of ARB. You have had an infection caused by ARB in the last 6 months. This might be a urine, bloodstream, chest or other type of infection and may have been on a previous visit to the hospital or from a sample that your General Practitioner sent to our lab.

Do I have to take part?

No, participation in the study is entirely voluntary. It is up to you whether or not you wish to take part. You will be given time to read the patient information sheet and ask any questions you wish about the study.

If you decide to take part you will be given this information sheet to keep and asked to sign a consent form. If you decide to take part you are still free to withdraw at any time and without giving a reason.

What will happen if I take part?

You will be asked to provide a stool sample to first check for the presence of the ESBL or CRE bacteria. If we don't find the bacteria you will not be able to continue in the study and any samples that you have already provided will be destroyed.

If ESBL or CRE bacteria are detected, you will be asked to provide a blood sample and female participants may be asked to provide a urine sample for a pregnancy test. Once we can confirm that it is safe for you to enter the study, you will be placed in one of two groups. This will be decided by chance. One group will receive FMT treatment, and the other will receive placebo (dummy) capsules. Both groups will be given five identical capsules to take each day for three days in a row. You will not know which group you are in until the end of the study.

Before taking the trial treatment, you will be asked to fast for 4 hours. You cannot eat any food during this time but you can drink water. You will also be asked to take a commonly prescribed medication called omeprazole. This works to reduce the amount of acid in the stomach. It will help protect the good bacteria in the capsules from being damaged.

For peer review only - http://bmjopen.bmj.com/site/about/guidelines.xhtml

You will then take all 5 capsules with water or squash. A member of the study team will stay with you for a short time while you take the capsules and check for any side effects. You can eat and drink as normal shortly after taking the capsules. The capsules will only be given on weekdays. If you are already staying in hospital, you will receive the treatment during your stay. If you are allowed to go home during this time, we will ask you to return to the hospital to complete the treatment. If you are not currently admitted to hospital, you will be asked to visit the hospital as an outpatient to complete the treatment.

You will be contacted by the study team by telephone after completing the treatment. This will happen 1 week, 3 months and 6 months after receiving treatment. You will be asked to provide a stool sample using the collection kit provided, and send it back to us in the post (postage will be pre-paid). You will be asked to complete a quality of life questionnaire over the phone. We will ask you how you have been feeling and if you are taking any new medications.

We also ask that you return to St Thomas' Hospital for a visit about 1 month after taking the capsules and we will ask you to bring a stool sample at that visit. We are able to reimburse you for the travel costs of visits to the hospital needed for the study, up to a maximum of £20 per visit.

In total you will be asked to provide five stool samples.

	Assessment	Treatment Day 1	Treatment Day 2	Treatment Day 3	Follow Up 1 week, 3 months, 6 months	Follow Up 1 month
	During Hospital Stay or Visit 1	During Hospital Stay or Visit 2	During Hospital Stay or Visit 3	During Hospital Stay or Visit 4	Telephone Call	Hospital Visit
Consent form	X					
Blood sample	X					X
Stool sample	X				X	X
Pregnancy test (females)	X					
Eligibility Check	X					
Basic health check	X	X	X	X		
Quality of Life Questionnaire	X				X	X
Fasting for 4 hours		X	X	X		
Capsule administration		X	X	X		
Side effects and medication check		X	X	X	X	X

For peer review only

With your consent we will send a letter to your GP to let them know that you are taking part in the trial.

What will happen to any samples I provide?

Stool samples will be processed in the FMT laboratory at St Thomas's Hospital and divided up into smaller samples. Some of the sample will be analysed at St Thomas' Hospital and some will be sent to KCL for analysis. They will be labelled with a study ID number that links the samples to who you are. This link will be kept securely by the study team and will not be shared with anybody else. No directly identifiable information will be used to label your samples.

You will be asked to provide 14 ml of blood (equivalent to 3 teaspoons) before entering the study. This is to check that it is safe for you to enter the study. If any of these tests have already been done as part of your normal care in the previous 5 days, we will not need to repeat them.

Will my samples be used in future research?

We will ask for your consent to take a blood sample and to use the stool samples that you have already provided for use in future research in addition to the samples mentioned above. The consent for storage of samples for future research is optional and will not affect your participation in the study in any way. If you do consent to this, we will ask you for 40 ml (8 teaspoons) before receiving the study treatment. This will be taken at the same time as the initial blood sample, so doesn't require an extra needle. We will also ask for a further 40 ml (8 teaspoons) at the face to face visit about 1 month after taking the capsules.

What are the possible benefits and disadvantages of taking part?

This study is designed to look at the safety and tolerability of FMT treatment to see if a larger study would show an improvement in wellbeing for patients with ARB infections. There are few treatments available to patients with antimicrobial resistant infections.

We hope that the knowledge we would gain from this study will improve our understanding of the way in which FMT works, and the role of the treatment in antimicrobial resistance. FMT appears to be safe in the considerable numbers of patients who have received it for other reasons.

If you are in the group that receives FMT, it is possible that it will help to reduce the ARB in found in your gut and reduce the risk of further infections. However, we will not know this until we have completed this study and future studies. You may not directly benefit from taking part in this study, but the information gained may help to improve the treatment of people with your condition in the future.

If you decide to take part in the study, you may be asked to attend the hospital more frequently than you would if you choose not to take part. You will also receive more regular input from a study doctor and nurse to monitor how you are feeling after the treatment.

What is the drug that is being tested?

For peer review only - http://bmjopen.bmj.com/site/about/guidelines.xhtml

FMT is the transfer of bacteria from the guts of healthy donors (taken from their poo) into the gut of a patient. FMT is produced by carefully selecting healthy individuals who have undergone an extensive health assessment and have been tested for a wide range of infections and other diseases. We follow National guidelines when selecting volunteer donors. The donor poo sample is processed in the laboratory to concentrate the healthy bacteria and remove most of the water (freeze drying) which leaves a small amount of powder. The powder is then packed into capsules which need to be swallowed.

FMT is currently used to treat patients with repeated Clostridioides difficile infection. In these patients, FMT is administered via a tube into the stomach or the lower bowel.

FMT is very effective and safe in treating this group of patients, with success rates of over 80%.

Some patients have experienced side effects, including belching, abdominal cramps and abdominal pain. Diarrhoea and constipation has also been reported.

We hope that producing FMT in capsules will help to minimise any side effects. We will monitor you closely while you are taking FMT and ask how you are feeling after receiving the treatment.

We would like you to report any unexpected symptoms or health events to the study team, even if you think it is not related to the treatment.

Omeprazole

Omeprazole reduces the amount of acid your stomach makes. It's a widely used treatment for indigestion, heartburn and acid reflux.

Most people who take omeprazole don't have any side effects, particularly if it is taken over a short period of time, as is the case here. If you do get a side effect, it's usually mild (such as abdominal pain, constipation, diarrhoea, dizziness and dry mouth) and will go away when you stop taking omeprazole.

How is FMT made?

After the healthy volunteers have been carefully screened and tested, the stool sample is brought to the FMT laboratory. Sterile salt water will be added to the stool and it will be filtered to remove any solid material and then be freeze-dried to remove water.

The resultant material is placed into capsules and stored frozen until it is required by the person who will receive it. Once a patient has been identified and consented to take part in the FERARO study, the capsules will be administered to the patient.

We will store a small amount of the stool sample to assess the types of bacteria present (microbiota analysis) so that we can compare to the stool samples provided by the patients that take the FMT.

For peer review only

These analyses will be performed at King's College, London for the blood samples. As part of a separate study the stool sample analysis will be performed at the National Institute for Biological Standards and Controls.

All samples will be completely anonymised. Patients who receive the FMT will not be given any information about who donated the samples.

Stool will be archived for thirty years for traceability, so that in the unlikely event of an infection occurring in the recipient the donor stool can be checked for infection also. The data will be anonymised and will only be accessible to members of the trials team. After this period it will be destroyed.

Who is organising and funding this study?

The doctor in charge of this study is Dr Simon Goldenberg. The study is funded by the National Institute of Health Research and is being sponsored by Guy's and St Thomas' NHS Foundation Trust.

Who has reviewed the study?

All research in the NHS is looked at by an independent group of people, called a Research Ethics Committee, to protect your interests. This study has been reviewed and approved by the London – City and East Research Ethics committee (reference 20/LO/0117.) It has also been reviewed by an independent review group and approved by the Health Research authority and the Medicines and Healthcare products Regulatory Agency.

What if something goes wrong?

If you have a concern about any aspect of this study, you should ask to speak to your study doctor who will do their best to answer your questions (contact details on page 2 of this information sheet).

If you remain unhappy and wish to complain formally, you can do this through the NHS Complaints procedure by contacting the Patient Advice Liaison Service (PALS) office.

Guy's and St Thomas' NHS Foundation Trust PALS
Contact number: 0207 188 8801 or pals@gstt.nhs.uk

Every care will be taken in the course of this study. However in the unlikely event that you are injured by taking part, compensation may be available.

In the event that something does go wrong and you are harmed during the research and this is due to someone's negligence then you may have grounds for a legal action for compensation against Guy's and St. Thomas' NHS Foundation Trust, but you may have to pay your legal costs.

For peer review only - http://bmjopen.bmj.com/site/about/guidelines.xhtml

How will we use information about you?

We will need to use information from you and from your medical records for this research project. This information will include your hospital number, name, date of birth, address and contact details. People will use this information to do the research or to check your records to make sure that the research is being done properly.

People who do not need to know who you are will not be able to see your name or contact details. Your data will have a code number instead. We will keep all information about you safe and secure.

Once we have finished the study, we will keep some of the data so we can check the results. We will write our reports in a way that no-one can work out that you took part in the study.

What are your choices about how your information is used?

You can stop being part of the study at any time, without giving a reason, but we will keep information about you that we already have.

If you agree to take part in this study, you will have the option to take part in future research using your data saved from this study.

Where can you find out more about how your information is used?

You can find out more about how we use your information at:
www.guysandstthomas.nhs.uk/research/patients/use-of-data.aspx
at www.hra.nhs.uk/information-about-patients/
by asking one of the research team or by sending an email to the Chief Investigator
simon.goldenberg@gstt.nhs.uk

What will happen to the results of the research study?

Once the data has been analysed we plan to publish the results in an international journal so that the information can benefit as many people as possible. We can provide you with a brief summary of the results of the study when available should you desire this. If you would like to be kept informed about the results of the study, please email the Chief Investigator using the details below.

Contact details:

Thank you for taking the time to read this information. If there is any other information you would like, please do not hesitate to contact the study team on the numbers below.

Study co-ordinator:	Dr Blair Merrick	Tel: 0207 188 7188 extension 53339
Research Nurse:	Karen Bisnauthsing	Tel: 0207 188 7188 extension 53339
Chief Investigator:	Dr Simon Goldenberg	Tel: 0207 188 8515

For peer review only - http://bmjopen.bmj.com/site/about/guidelines.xhtml

Table 3. Healthy donor inclusion and exclusion criteria

Inclusion criteria

- 18-60 years of age **AND**
- BMI 18-30

Exclusion criteria

- Received antimicrobials within past 3 months
- Known prior exposure to HIV and/or viral hepatitis
- Known previous or latent tuberculosis
- Risk factors for blood borne viruses within the previous 6 months
- Received live attenuated vaccine within past 6 months
- Underlying gastrointestinal condition/ or unexplained symptoms including acute diarrhoea in 2 weeks prior to donating
- Family history of any significant gastrointestinal condition
- History of atopy, systemic autoimmune condition, diabetes, neurological or psychiatric condition or known risk of prion disease. History of chronic pain syndrome including chronic fatigue and fibromyalgia, or malignancy.
- Taking any regular medications
- History of taking proton pump inhibitors or immunosuppressive medications within the past 3 months
- Ever received growth hormone, insulin from cows or clotting factor concentrates
- Received an experimental medicine or vaccine within the past 6 months
- Travelled to a tropical country within the past 6 months

For peer review only

Table 4. Donor screening and eligibility questionnaire

Guy's and St Thomas' NHS
NHS Foundation Trust

Faecal Microbiota Transplant (FMT) –Donor programme

Donor name	
Donor date of birth	
Donor Hospital number	
Donor number	
Donor contact details – telephone / email	
Name of assessor	
Position	
Date of assessment	

Age	Exclude if <18 or >60
Gender	☐ Male ☐ Female
Ethnicity	☐ White - White British<br>☐ White - White Irish<br>☐ White - Other<br>☐ Mixed race – White and Black Caribbean<br>☐ Mixed race – White and Black African<br>☐ Mixed race – White and Asian<br>☐ Mixed race – Other<br>☐ Asian or Asian British – Indian<br>☐ Asian or Asian British – Bangladeshi<br>☐ Asian or Asian British – Pakistani<br>☐ Asian or Asian British – Other<br>☐ Black or Black British – Caribbean<br>☐ Black or Black British – African<br>☐ Black or Black British – Other<br>☐ Chinese<br>☐ Other
Height	cm
Weight	

For peer review only - http://bmjopen.bmj.com/site/about/guidelines.xhtml

	kg
BMI	Exclude if BMI ≤18 or ≥30
Has your weight changed by more than 5lb / 2kg in the past 6 months?	☐ Yes ☐ No<br><br>Detail:
Describe your diet (as many as apply):	☐ Omnivore<br>☐ Vegetarian<br>☐ Vegan<br>☐ Kosher<br>☐ Halal<br>☐ Raw food only<br>☐ Pescatarian<br>☐ No red meat<br>☐ Low carbohydrate<br>☐ Lactose free<br>☐ Gluten free<br>☐ Other
How many portions of fruit and vegetables do you consume per day?	☐ one or less<br>☐ two to three<br>☐ three to four<br>☐ five to six<br>☐ seven or more
How many servings of cow, sheep or goat's milk do you consume per day?	☐ one or less<br>☐ two to three<br>☐ three to four<br>☐ five to six<br>☐ seven or more
Alcohol – units/week	
Smoking/day	
Do you take any illicit drugs?	☐ Yes ☐ No<br><br>Exclude if YES
Normal bowel habit – average Bristol Stool Consistency	☐ 1<br>☐ 2<br>☐ 3<br>☐ 4<br>☐ 5<br>☐ 6<br>☐ 7<br><br>Exclude if 1, 6 or 7

For peer review only - http://bmjopen.bmj.com/site/about/guidelines.xhtml

Normal bowel habit – average frequency	☐ >2/day<br>☐ once to twice daily<br>☐ once / 2 days<br>☐ <once / 2 days<br><br>Exclude if active diarrhoea (>3 UBM/day for at least 2 consecutive days)
Have you ever been rejected as a blood donor/told not to donate?<br>If yes, why?	☐ Yes ☐ No<br><br>Exclude if YES
What is your country of birth?	
Have you ever resided in another country (other than UK) for >5 years?<br>If so which countries and when?	☐ Yes ☐ No
Do you currently have a profession that is associated with an increased risk of blood-borne transmissible diseases (e.g. daily contact with patients/inmates)?<br>If yes, what profession?	☐ Yes ☐ No<br><br>Exclude if health/social care worker with direct patient contact
Have you ever had a needle-stick or injury from a blood contaminated object from someone else?<br><br>If yes, when and how was this follow up?	☐ Yes ☐ No
Have you ever injected yourself or been injected with illegal or non-prescribed drugs including body building drugs or cosmetics (even if this was only once or a long time ago?)	☐ Yes ☐ No<br><br>Exclude if YES
Have you ever had a tattoo?<br>If yes, when and in which country was it performed?	☐ Yes ☐ No<br><br>Exclude if within past 6 months
Have you ever had a piercing?<br>If yes, when and in which country was it performed?	☐ Yes ☐ No<br><br>Exclude if within past 6 months
Have you ever had acupuncture?<br>If yes, when and in which country was it performed?	☐ Yes ☐ No<br><br>Exclude if within past 4 months
Have you ever had an operation or undergone clinical treatment in	☐ Yes ☐ No

For peer review only

For peer review only - http://bmjopen.bmj.com/site/about/guidelines.xhtml

a hospital with poor hygienic conditions?<br>If yes, when and in which country was it performed?	
Have you ever had a rare infectious disease (e.g. tuberculosis, malaria, trypanosomiasis)?<br>If yes, when and which disease?	☐ Yes ☐ No
Have you ever been vaccinated against Hepatitis A or B?<br>If yes, which?	☐ Yes ☐ No
In the last 12 months have you had sex with anyone who is HIV positive?	☐ Yes ☐ No<br>Exclude if Yes
In the last 12 months have you had sex with anyone with hepatitis B, hepatitis C or HTLV?	☐ Yes ☐ No<br>Exclude if Yes
In the last 12 months have you had sex with anyone who has ever been given money or drugs for sex?	☐ Yes ☐ No<br>Exclude if Yes
In the last 12 months have you had sex with anyone who has ever injected drugs?	☐ Yes ☐ No<br>Exclude if Yes
In the last 12 months have you had sex with anyone who may ever have had sex in parts of the world where HIV/AIDS is very common (this includes most countries in Africa)?	☐ Yes ☐ No<br>Exclude if Yes
**Male donors ONLY:**<br>In the last 12 months have you ever had oral or anal sex with a man, with or without a condom?	☐ Yes ☐ No<br>Exclude if Yes
**Female donors ONLY:**<br>In the last 12 months have you had sex with a man who has ever had oral or anal sex with another man, with or without a condom?	☐ Yes ☐ No<br>Exclude if Yes
Have you ever been treated for an intestinal infection?<br>If yes, which one and when?	☐ Yes ☐ No
Do you have any gastro-intestinal conditions:<br>Barretts Oesophagus<br>Coeliac disease<br>Diverticular disease	☐ Yes ☐ No<br>☐ Yes ☐ No<br>☐ Yes ☐ No<br>☐ Yes ☐ No

For peer review only - http://bmjopen.bmj.com/site/about/guidelines.xhtml

Bariatric surgery<br>Gastric ulcer<br>Gasto-oesophageal reflux disease<br>Hepatitis<br>H. pylori infection<br>Crohns disease<br>Ulcerative colitis<br>Other inflammatory bowel diseases<br>Irritable Bowel Syndrome<br>Lactose intolerance<br>Liver disease<br>Pancreatitis<br>Gastrointestinal malignancy or polyps	☐ Yes ☐ No<br>☐ Yes ☐ No<br>☐ Yes ☐ No<br>☐ Yes ☐ No<br>☐ Yes ☐ No<br>☐ Yes ☐ No<br>☐ Yes ☐ No<br>☐ Yes ☐ No<br>☐ Yes ☐ No<br>☐ Yes ☐ No<br>☐ Yes ☐ No<br>☐ Yes ☐ No<br><br>Exclude if Inflammatory Bowel Disease, Irritable Bowel Syndrome, GI malignancy, Hepatitis
Is there any family history of inflammatory Bowel Disease or colorectal cancer?	☐ Yes ☐ No<br><br>Exclude if Yes
Have you taken any antibiotics in the last 3 months?	☐ Yes ☐ No<br><br>Exclude if Yes
Have you had a fever in the last 2 weeks?	☐ Yes ☐ No<br><br>Exclude if Yes
Have you received a live vaccination within the past 6 months?	☐ Yes ☐ No<br><br>Exclude if Yes
Have you ever been incarcerated in prison?	☐ Yes ☐ No<br><br>Exclude if in past 4 months
Have you ever been immunosuppressed (e.g. during treatment for cancer, or for a solid organ transplant)?<br>If yes, when and why?	☐ Yes ☐ No
Have you ever had major gastrointestinal surgery?<br>If yes, when and why?	☐ Yes ☐ No<br><br>Relative exclusion criteria
Have you ever suffered from metabolic syndrome or diabetes?	☐ Yes ☐ No<br><br>Exclude if Yes
Have you ever suffered from any autoimmune condition (e.g. rheumatoid), asthma or eczema?<br>If yes, which, and do you take any treatment?	☐ Yes ☐ No<br><br>Relative exclusion criteria
Have you ever had any chronic pain or fatigue syndromes e.g.	☐ Yes ☐ No

For peer review only - http://bmjopen.bmj.com/site/about/guidelines.xhtml

chronic fatigue syndrome, fibromyalgia?<br>If yes, which?	Exclude if Yes
Have you any history of CJD or other prion disease in your family? If yes, please specify<br>Patients should be considered to be at risk from genetic forms of CJD if they have or have had<br>1. Genetic testing, which has indicated they are at significant risk of developing CJD or other prion disease<br>2. A blood relative known to have a genetic mutation indicative of genetic CJD or other prion disease<br>3. Two or more blood relatives affected by CJD or other prion disease	☐ Yes ☐ No<br><br>Exclude if Yes
Have you ever received growth hormone or gonadotrophic treatment? If yes, please specify;<br>i) Whether the hormone was derived from human pituitary glands<br>ii) The year of the treatment<br>iii) Whether the treatment was received in the UK or in another country<br><br>Recipients of hormone derived from human pituitary glands e.g. growth hormone or gonadotrophin have been identified as potentially at risk of CJD. In the UK, the use of human growth hormone was stopped in 1985 but human-derived products may have been continued to be used in other countries. In the UK, the use of human-derived gonadotrophin was discontinued in 1973 but may have been continued in other countries after this time.	☐ Yes ☐ No<br><br>Exclude if Yes

For peer review only - http://bmjopen.bmj.com/site/about/guidelines.xhtml

Have you ever had surgery on your brain or spinal cord?<br>People who underwent intradural neurosurgical or spinal procedures before August 1992 may have received a graft of human-derived dura mater and should be treated as at increased risk, unless evidence can be provided that human-derived dura mater was not used. Patients who received a graft of human-derived dura mater between 1980 and August 1992 are at increased risk of both sporadic CJD and vCJD.	☐ Yes ☐ No<br><br>Exclude if Yes
Are you normally resident in the UK?<br>If No state country of usual residence	☐ Yes ☐ No
Which countries have you visited in the last 12 months and what was the duration of stay?	Relative exclusions apply to tropical countries
In the past 12 months have you been admitted to a hospital in a country other than the UK?<br><br>If yes state when and which countries	☐ Yes ☐ No
Are you taking any regular medications?<br><br>List all current medications:	☐ Yes ☐ No<br><br>Exclude if any regular prescribed drugs (except OCP)

For peer review only - http://bmjopen.bmj.com/site/about/guidelines.xhtml

For peer review only

For peer review only - http://bmjopen.bmj.com/site/about/guidelines.xhtml

Reporting checklist for protocol of a clinical trial.

Based on the SPIRIT guidelines.

Instructions to authors

Complete this checklist by entering the page numbers from your manuscript where readers will find each of the items listed below.

Your article may not currently address all the items on the checklist. Please modify your text to include the missing information. If you are certain that an item does not apply, please write "n/a" and provide a short explanation.

Upload your completed checklist as an extra file when you submit to a journal.

In your methods section, say that you used the SPIRITreporting guidelines, and cite them as:

Chan A-W, Tetzlaff JM, Altman DG, Laupacis A, Gøtzsche PC, Krleža-Jerić K, Hróbjartsson A, Mann H, Dickersin K, Berlin J, Doré C, Parulekar W, Summerskill W, Groves T, Schulz K, Sox H, Rockhold FW, Rennie D, Moher D. SPIRIT 2013 Statement: Defining standard protocol items for clinical trials. Ann Intern Med. 2013;158(3):200-207

		Reporting Item	Page Number
**Administrative information**			
Title	#1	Descriptive title identifying the study design, population, interventions, and, if applicable, trial acronym	1
Trial registration	#2a	Trial identifier and registry name. If not yet registered, name of intended registry	2
Trial registration: data set	#2b	All items from the World Health Organization Trial Registration Data Set	4
Protocol version	#3	Date and version identifier	2
Funding	#4	Sources and types of financial, material, and other support	13
Roles and responsibilities: contributorship	#5a	Names, affiliations, and roles of protocol contributors	13

For peer review only

Roles and responsibilities: sponsor contact information	#5b	Name and contact information for the trial sponsor	13
Roles and responsibilities: sponsor and funder	#5c	Role of study sponsor and funders, if any, in study design; collection, management, analysis, and interpretation of data; writing of the report; and the decision to submit the report for publication, including whether they will have ultimate authority over any of these activities	13, 14
Roles and responsibilities: committees	#5d	Composition, roles, and responsibilities of the coordinating centre, steering committee, endpoint adjudication committee, data management team, and other individuals or groups overseeing the trial, if applicable (see Item 21a for data monitoring committee)	12
**Introduction**			
Background and rationale	#6a	Description of research question and justification for undertaking the trial, including summary of relevant studies (published and unpublished) examining benefits and harms for each intervention	3
Background and rationale: choice of comparators	#6b	Explanation for choice of comparators	4
Objectives	#7	Specific objectives or hypotheses	4
Trial design	#8	Description of trial design including type of trial (eg, parallel group, crossover, factorial, single group), allocation ratio, and framework (eg, superiority, equivalence, non-inferiority, exploratory)	5
**Methods: Participants, interventions, and outcomes**			
Study setting	#9	Description of study settings (eg, community clinic, academic hospital) and list of countries where data will be collected. Reference to where list of study sites can be obtained	7

For peer review only

Eligibility criteria	#10	Inclusion and exclusion criteria for participants. If applicable, eligibility criteria for study centres and individuals who will perform the interventions (eg, surgeons, psychotherapists)	6
Interventions: description	#11a	Interventions for each group with sufficient detail to allow replication, including how and when they will be administered	8
Interventions: modifications	#11b	Criteria for discontinuing or modifying allocated interventions for a given trial participant (eg, drug dose change in response to harms, participant request, or improving / worsening disease)	8
Interventions: adherance	#11c	Strategies to improve adherence to intervention protocols, and any procedures for monitoring adherence (eg, drug tablet return; laboratory tests)	8
Interventions: concomitant care	#11d	Relevant concomitant care and interventions that are permitted or prohibited during the trial	8
Outcomes	#12	Primary, secondary, and other outcomes, including the specific measurement variable (eg, systolic blood pressure), analysis metric (eg, change from baseline, final value, time to event), method of aggregation (eg, median, proportion), and time point for each outcome. Explanation of the clinical relevance of chosen efficacy and harm outcomes is strongly recommended	9
Participant timeline	#13	Time schedule of enrolment, interventions (including any run-ins and washouts), assessments, and visits for participants. A schematic diagram is highly recommended (see Figure)	9
Sample size	#14	Estimated number of participants needed to achieve study objectives and how it was determined, including clinical and statistical assumptions supporting any sample size calculations	11
Recruitment	#15	Strategies for achieving adequate participant enrolment to reach target sample size	7
**Methods: Assignment of interventions (for controlled trials)**			
Allocation: sequence generation	#16a	Method of generating the allocation sequence (eg, computer-generated random numbers), and list of any factors for stratification. To reduce predictability of a random sequence, details of any planned restriction (eg, blocking) should be	8

For peer review only

		provided in a separate document that is unavailable to those who enrol participants or assign interventions	
Allocation concealment mechanism	#16b	Mechanism of implementing the allocation sequence (eg, central telephone; sequentially numbered, opaque, sealed envelopes), describing any steps to conceal the sequence until interventions are assigned	8
Allocation: implementation	#16c	Who will generate the allocation sequence, who will enrol participants, and who will assign participants to interventions	8
Blinding (masking)	#17a	Who will be blinded after assignment to interventions (eg, trial participants, care providers, outcome assessors, data analysts), and how	8
Blinding (masking): emergency unblinding	#17b	If blinded, circumstances under which unblinding is permissible, and procedure for revealing a participant's allocated intervention during the trial	8
**Methods: Data collection, management, and analysis**			
Data collection plan	#18a	Plans for assessment and collection of outcome, baseline, and other trial data, including any related processes to promote data quality (eg, duplicate measurements, training of assessors) and a description of study instruments (eg, questionnaires, laboratory tests) along with their reliability and validity, if known. Reference to where data collection forms can be found, if not in the protocol	12
Data collection plan: retention	#18b	Plans to promote participant retention and complete follow-up, including list of any outcome data to be collected for participants who discontinue or deviate from intervention protocols	12
Data management	#19	Plans for data entry, coding, security, and storage, including any related processes to promote data quality (eg, double data entry; range checks for data values). Reference to where details of data management procedures can be found, if not in the protocol	12
Statistics: outcomes	#20a	Statistical methods for analysing primary and secondary outcomes. Reference to where other details of the statistical analysis plan can be found, if not in the protocol	12

For peer review only

Statistics: additional analyses	#20b	Methods for any additional analyses (eg, subgroup and adjusted analyses)	12
Statistics: analysis population and missing data	#20c	Definition of analysis population relating to protocol non-adherence (eg, as randomised analysis), and any statistical methods to handle missing data (eg, multiple imputation)	12
**Methods: Monitoring**			
Data monitoring: formal committee	#21a	Composition of data monitoring committee (DMC); summary of its role and reporting structure; statement of whether it is independent from the sponsor and competing interests; and reference to where further details about its charter can be found, if not in the protocol. Alternatively, an explanation of why a DMC is not needed	12
Data monitoring: interim analysis	#21b	Description of any interim analyses and stopping guidelines, including who will have access to these interim results and make the final decision to terminate the trial	12
Harms	#22	Plans for collecting, assessing, reporting, and managing solicited and spontaneously reported adverse events and other unintended effects of trial interventions or trial conduct	12
Auditing	#23	Frequency and procedures for auditing trial conduct, if any, and whether the process will be independent from investigators and the sponsor	12
**Ethics and dissemination**			
Research ethics approval	#24	Plans for seeking research ethics committee / institutional review board (REC / IRB) approval	13
Protocol amendments	#25	Plans for communicating important protocol modifications (eg, changes to eligibility criteria, outcomes, analyses) to relevant parties (eg, investigators, REC / IRBs, trial participants, trial registries, journals, regulators)	13
Consent or assent	#26a	Who will obtain informed consent or assent from potential trial participants or authorised surrogates, and how (see Item 32)	7

For peer review only

Consent or assent: ancillary studies	#26b	Additional consent provisions for collection and use of participant data and biological specimens in ancillary studies, if applicable	7
Confidentiality	#27	How personal information about potential and enrolled participants will be collected, shared, and maintained in order to protect confidentiality before, during, and after the trial	7
Declaration of interests	#28	Financial and other competing interests for principal investigators for the overall trial and each study site	14
Data access	#29	Statement of who will have access to the final trial dataset, and disclosure of contractual agreements that limit such access for investigators	12
Ancillary and post trial care	#30	Provisions, if any, for ancillary and post-trial care, and for compensation to those who suffer harm from trial participation	12
Dissemination policy: trial results	#31a	Plans for investigators and sponsor to communicate trial results to participants, healthcare professionals, the public, and other relevant groups (eg, via publication, reporting in results databases, or other data sharing arrangements), including any publication restrictions	12
Dissemination policy: authorship	#31b	Authorship eligibility guidelines and any intended use of professional writers	12
Dissemination policy: reproducible research	#31c	Plans, if any, for granting public access to the full protocol, participant-level dataset, and statistical code	12
**Appendices**			
Informed consent materials	#32	Model consent form and other related documentation given to participants and authorised surrogates	3
Biological specimens	#33	Plans for collection, laboratory evaluation, and storage of biological specimens for genetic or molecular analysis in the current trial and for future use in ancillary studies, if applicable	9

The SPIRIT checklist is distributed under the terms of the Creative Commons Attribution License CC-BY-ND 3.0. This checklist was completed on 26. March 2020 using https://www.goodreports.org/, a tool made by the EQUATOR Network in collaboration with Penelope.ai

For peer review only
