# Supplementary material for: Faecal microbiota transplant to ERadicate gastrointestinal carriage of Antibiotic Resistant Organisms (FERARO): a prospective, randomised placebo-controlled feasibility trial
Source: BMJ Open. 2020 May 25;10(5):e038847. doi: 10.1136/bmjopen-2020-038847 (PMC7252984; doi:10.1136/bmjopen-2020-038847)
Supplement: Reviewer comments [file bmjopen-2020-038847.reviewer_comments.pdf]

PEER REVIEW HISTORY

BMJ Open publishes all reviews undertaken for accepted manuscripts. Reviewers are asked to complete a checklist review form (**http://bmjopen.bmj.com/site/about/resources/checklist.pdf**) and are provided with free text boxes to elaborate on their assessment. These free text comments are reproduced below.

ARTICLE DETAILS

**TITLE (PROVISIONAL)**	Protocol: A prospective, randomised placebo-controlled feasibility trial of Faecal microbiota Transplant to ERadicate gastrointestinal carriage of Antibiotic Resistant Organisms (FERARO)
**AUTHORS**	Merrick, Blair; Robinson, Emily; Bunce, Catey; Allen, Liz; BIsnauthsing, Karen; Izundu, Chi Chi; Bell, Jordana; Amos, Gregory; Shankar-Hari, Manu; Goodman, Anna; Shawcross, Debbie; Goldenberg, Simon

This article was not externally reviewed at BMJ Open. Protocols that have been independently assessed prior to submission to BMJ Open are usually fast-tracked to publication on the grounds that further substantial changes will not be possible. This independent assessment will usually be external, independent review for both a substantial grant award from a non-commercial or government funder and ethics approval.
